# NEBULA is a fast negative binomial mixed model for differential or co-expression analysis of large-scale multi-subject single-cell data

**DOI:** 10.1038/s42003-021-02146-6

**Published:** 2021-05-26

**Authors:** Liang He, Jose Davila-Velderrain, Tomokazu S. Sumida, David A. Hafler, Manolis Kellis, Alexander M. Kulminski

**Affiliations:** 1 Biodemography of Aging Research Unit, Social Science Research Institute, Duke University, Durham, NC USA; 2 Broad Institute of MIT and Harvard, Cambridge, MA USA; 3 Computer Science and Artificial Intelligence Laboratory, MIT, Cambridge, MA USA; 4 Departments of Neurology and Immunobiology, Yale School of Medicine, New Haven, CT USA; 5 Department of Cardiovascular Medicine, University of Tokyo Graduate School of Medicine, Tokyo, Japan

**Keywords:** Computational models, Transcriptomics, Alzheimer's disease

## Abstract

The increasing availability of single-cell data revolutionizes the understanding of biological mechanisms at cellular resolution. For differential expression analysis in multi-subject single-cell data, negative binomial mixed models account for both subject-level and cell-level overdispersions, but are computationally demanding. Here, we propose an efficient NEgative Binomial mixed model Using a Large-sample Approximation (NEBULA). The speed gain is achieved by analytically solving high-dimensional integrals instead of using the Laplace approximation. We demonstrate that NEBULA is orders of magnitude faster than existing tools and controls false-positive errors in marker gene identification and co-expression analysis. Using NEBULA in Alzheimer’s disease cohort data sets, we found that the cell-level expression of *APOE* correlated with that of other genetic risk factors (including *CLU, CST3, TREM2*, C1q, and *ITM2B*) in a cell-type-specific pattern and an isoform-dependent manner in microglia. NEBULA opens up a new avenue for the broad application of mixed models to large-scale multi-subject single-cell data.

## Abstract (web summary)

The application of negative binomial mixed models (NBMMs) to single-cell data is computationally demanding. To address this issue, Liang He et al. have developed NEBULA, an efficient algorithm that can analyze differential gene expression or co-expression networks in multi-subject single-cell data sets, and validate it on snRNA-seq and scRNA-seq data sets comprising ~200k cells from cohorts of Alzheimer’s disease and multiple sclerosis patients.

## Introduction

Single-cell genomic profiling technology has revolutionized our understanding of biology to an unprecedented resolution. With the recent advances in high-throughput single-cell profiling techniques^1–3^, large-scale multi-subject single-cell RNA-seq (scRNA-seq) and ATAC-seq (scATAC-seq) data are becoming increasingly accessible. Notably, through droplet-based technology, tens to hundreds of thousands of cells from multiple subjects can be sequenced in parallel within a single batch^2,4^. As an example, a recent large-scale population single-nucleus RNA-seq (snRNA-seq) data in the human frontal cortex involving ~80,000 nuclei from a cohort comprising 24 Alzheimer’s disease (AD) patients and 24 healthy controls^5^ provide biological information in much finer granularity compared to bulk RNA-seq data. Projects profiling more than one million single cells from hundreds of subjects are underway.

The drastically increasing magnitude of sample size, however, poses a serious computational challenge when trying to apply conventional transcriptomics analysis for differential expression, expression quantitive trait loci (eQTLs), and co-expression to large-scale scRNA-seq data. This situation is in contrast to that of statistical models in bulk RNA-seq analysis, in which more emphasis is placed upon building a robust estimate under a small sample size (e.g., regularization of standard errors and robust estimation of overdispersion parameters^6–8^). Unlike bulk RNA-seq data, multi-subject scRNA-seq data, particularly using the droplet-based technology, often comprise many cells but are characterized by high sparsity and a hierarchical structure. Owing to the hierarchical nature of multi-subject scRNA-seq data, it is appropriate to decompose the overdispersion into the subject and cell levels. One of the standard approaches to handle hierarchical count responses is a negative binomial mixed-effects model (NBMM), which introduces independent random effects to take into account the overdispersion at both levels. NBMMs increase the statistical power by a more accurate specification of the overdispersion structure, and also helps in eliminating spurious associations to detect marker genes of a cell cluster, as we will show in our simulation. Here, we focus on NBMMs rather than a zero-inflated model because multiple recent studies show that a zero-inflated model might be redundant for unique molecular identifiers (UMI)-based single-cell data^9–11^. This is also consistent with the observations for the vast majority of genes in our analysis of real data.

A plethora of estimation methods have been proposed for NBMMs^12–17^, and it is the invention of these fast algorithms that popularizes its practical use. However, the computational efficiency of existing tools^15,18,19^ is still insufficient for its broad application to large-scale multi-subject scRNA-seq data. Owing to the intractable marginal likelihood in NBMMs, current estimation algorithms generally rely on a two-layer iteration procedure, which often converges slowly. To address the computational burden, we propose a NEgative Binomial mixed model Using Large-sample Approximation (NEBULA), a novel fast algorithm for association analysis of scRNA-seq data using an NBMM. As the core idea in NEBULA, we developed an analytical approximation of the high-dimensional integral for the marginal likelihood of the NBMM, by leveraging the common feature of scRNA-seq data of having many cells per subject. The improvement of computational efficiency is achieved by avoiding the two-layer optimization and converging within fewer steps.

We demonstrated the efficiency and accuracy of NEBULA through an extensive simulation study. As NEBULA uses approximation based on large numbers, we investigated the number of cells and subjects required to accurately estimate the subject-level and cell-level overdispersions. We also investigated the performance of NEBULA in dealing with lowly expressed genes and controlling the false-positive errors when testing fixed-effects predictors. It should be noted that a predictor of interest can be classified as a subject-level or a cell-level variable. The first category includes variables that share the same value across all cells from the same subject, for example, disease or treatment groups, genetic variants, and subject-level covariates such as age and sex. Variables with different values across individual cells such as cell cycle stages, subpopulation memberships, and cell-level gene expression belong to the second category. For testing associations with cell-level variables, it is known that ignoring the subject-level random effects would lead to inflated *p*-values^20^. In contrast, there is much less literature on the performance of testing subject-level predictors in cell-level data. It is well known that the subject-level random effects need to be included in the model when the predictor of interest is also a subject-level variable. Otherwise, the type I error rate would be inflated due to an underestimated standard error of the effect size. A detailed derivation of such underestimation is given in^21,22^. In this study, we found that testing a subject-level variable was highly sensitive to the estimation of the subject-level variance component and the assumption of the distribution of the random effects when the number of subjects is small.

To date, few studies have investigated the relative contributions of the subject-level and cell-level overdispersions to cell-type-specific gene expression. Overdispersion often results from failing to include important explanatory variables or account for a hierarchical structure in the data^23^. To explore these problems in a specific biological context, we used NEBULA to decompose gene-specific overdispersion of large-scale snRNA-seq data in the human frontal cortex from an AD cohort^5^. We further explored what factors might contribute to both overdispersions. We showed that NEBULA reduced false positives in selecting cell-type marker genes, especially when the numbers of cells across subjects were unbalanced. As a direct application of NEBULA, we performed a cell-level transcriptomic co-expression analysis of *APOE*, the strongest genetic risk factor of AD, and investigated its cell-type-specific regulatory mechanisms in microglia and astrocytes, both of which are known to abundantly express *APOE* and to undergo cell activation transitions in the context of AD pathology. This application of NEBULA allowed us to identify both cell-type and isoform-specific coregulatory interactions of *APOE* that might be relevant for mediating its disease-modifying effects at a molecular level.

## Results

### Overview of NEBULA

**Fig. 1:**
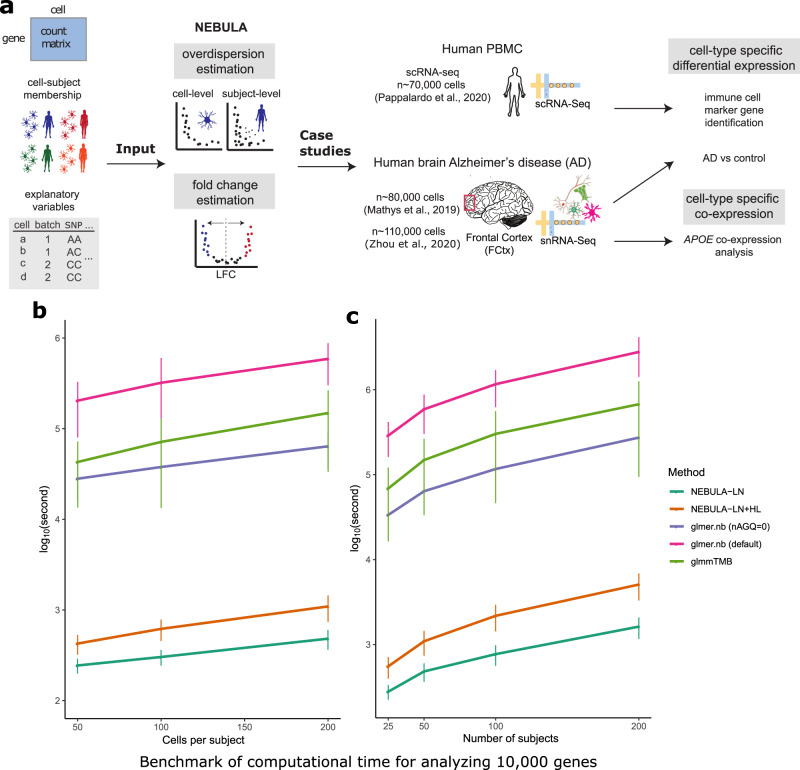
Overview of the input, output, and computational efficiency of NEBULA. **a** A flowchart of NEBULA including the input data, the major estimation steps in NEBULA, and the analyses that were conducted using NEBULA in the application to the real data^5,27,78^. **b** The computational time (measured in log_10_(seconds)) of fitting an NBMM for 10,000 genes with respect to CPS by NEBULA, *glmer.nb*^18^, and *glmmTMB*^19^. The number of subjects was set at 50. **c** The computational time (measured in log_10_(seconds)) of fitting an NBMM for 10,000 genes with respect to the number of subjects. The error bars represent one standard deviation of *n* = 10,000 genes. The CPS value was set at 200. Two fixed-effects predictors were included in the NBMM. The average benchmarks were summarized from scenarios of varying subject-level and cell-level overdispersions and the CPC value of a gene ranging from exp(−4) to 1. CPS: cells per subject. CPC: counts per cell.

NEBULA takes as an input the raw count matrix of a single-cell data set and a mapping between cells and subjects (Fig. 1a). The variation of raw counts in scRNA-seq data comes from two sources, a Poisson sampling process and overdispersion originating from biological heterogeneity and technical or experimental noise. Cells in large-scale single-cell data are often collected from multiple subjects or batches, leading to an apparent hierarchical structure. To model this structure, NEBULA decomposes the total overdispersion into subject-level (i.e., between-subject) and cell-level (i.e., within-subject) components using a random-effects term parametrized by ${\sigma }^{2}$ and the overdispersion parameter $\phi$ in the negative binomial distribution (Fig. 1a). The subject-level overdispersion captures the contributions from, e.g., eQTLs, technical artifacts, batch effects, and other subject-level covariates. The cell-level overdispersion reflects the variation of gene expression due to differences in, e.g., cell cycle, cell-type subpopulation, and other cell-level covariates such as ribosome RNA fraction. Generally, the estimation of an NBMM is computationally intensive because the marginal likelihood is intractable due to high-dimensional integrals of the random effects. A standard estimation method for generalized linear mixed models (GLMMs) using penalized quasi-likelihood (PQL)^12^ involves a two-step iterative procedure in which the variance of the random effects is first estimated and is subsequently used to estimate the coefficients of the variables of interest. In genome-wide association studies (GWAS), the variance component is often estimated only once under the null model. In contrast, in scRNA-seq or scATAC-seq data, such a two-step procedure has to be carried out for each of tens of thousands of genes or peak regions, giving rise to a considerably more challenging requirement. NEBULA achieves its speed gain by introducing an approximate analytical marginal likelihood derived by leveraging the fact that single-cell data often include a large number of cells for each subject. This approximation, referred to as NEBULA-LN, eliminates the computationally demanding two-layer high-dimensional optimization procedure, and therefore substantially reduces the computational time for estimating the overdispersions. For situations in which NEBULA-LN fails to achieve accurate estimates of the subject-level overdispersion, we use NEBULA-LN to estimate only the cell-level overdispersion and implemented a fast estimation procedure, referred to as NEBULA-HL, based on a hierarchical likelihood (h-likelihood) for estimating the subject-level overdispersion. As only one overdispersion is optimized in the h-likelihood, NEBULA-HL converges in much fewer steps than standard estimation methods.

### NEBULA is computationally efficient

We compared NEBULA with existing statistical tools for estimating NBMMs to evaluate its computational efficiency. In addition to NEBULA-LN, we assessed a combination of NEBULA-LN and NEBULA-HL (denoted by NEBULA-LN + HL), in which we first used NEBULA-LN to estimate the cell-level overdispersion, and then fixed this estimate in NEBULA-HL to obtain the subject-level overdispersion. For comparison, we included the *glmer.nb* function from the *lme4* R package^18^, the *glmmTMB* function from the *glmmTMB* R package^19^, and the *inla* function from the *INLA* R package^15^. The *glmmTMB* package utilizes the Template Model Builder automatic differentiation engine, and the *INLA* package uses an efficient Bayesian framework based on integrated nested Laplace approximations (LAs). We did not include methods based on adaptive Gaussian quadrature (AGQ) (*glmer.nb* with nAGQ>1)^24,25^ or Bayesian methods using Markov chain Monte Carlo (MCMC)^26^ because of their computational intensity. The Gaussian variational approximation method^14^ can be promising but has not been implemented for the NBMM.

Given a simulated data set comprising 50 subjects and cells per subject (CPS) = 200 (a total of ~10,000 cells), it took ~400 s for NEBULA-LN and ~1000 s for NEBULA-LN + HL to analyze 10,000 genes using one CPU thread (Fig. 1b). These benchmarks were on average >100-fold and ~50-fold faster than *glmer.nb* with nAGQ = 0, respectively. It took much longer for *glmer.nb* with the default setting and *glmmTMB*. We failed to obtain the full benchmarks for *inla* because it took an extremely long time to finish in some scenarios. Generally, the running time of *inla* was between *glmmTMB* and the default *glmer.nb*. The computational time of all methods increased almost linearly with CPS, which was expected because the random effects are assumed to be independent. We also observed that the computational time scaled in a similar trend across these methods with the increasing number of subjects (Fig. 1c). In all settings, NEBULA-LN + HL was ~2–3x slower than NEBULA-LN but was still much faster than *glmer.nb*. In our following analysis of ~34,000 excitatory neurons from 48 subjects in the snRNA-seq data adopted from ref. ^5^, NEBULA accomplished an association analysis of ~16,000 genes for identifying marker genes in ~40 min, compared to ~67 hours using *glmer.nb* with nAGQ = 0. When the number of fixed-effects predictors increased from two to ten, the computational time of both NEBULA and *glmer.nb* with nAGQ=0 grew modestly, while the default *glmer.nb* increased by ~10x (Supplementary Fig. S1). This substantial increase of the computational time in the default *glmer.nb* is expected because it estimates the fixed effects along with the overdispersions in the outer layer of the iterative procedure using a derivative-free method. The number of iterations of such an optimization method rises drastically when the dimension of the parameter space increases. Altogether, these comparisons demonstrate the superior computational efficiency of NEBULA relative to existing alternatives.

### NEBULA decomposes cell-level and subject-level overdispersions

The approximation proposed in NEBULA-LN provides asymptotically consistent estimates (Supplementary Note A.5). Nevertheless, it is crucial to assess its practical performance under a finite sample size. As the above benchmarks indicated that NEBULA-LN had a huge speed advantage over NEBULA-HL, the basic strategy in NEBULA is to first fit the data using NEBULA-LN and apply NEBULA-HL only in situations where NEBULA-LN performs poorly. We, therefore, conducted a comprehensive simulation study to investigate under which situations NEBULA-LN could produce sufficiently accurate estimates. The estimation accuracy of NEBULA-LN is primarily affected by CPS, the magnitude of the cell-level dispersion, and the coefficient of variation (CV) of the scaling factor (see the Methods section). We thus focus on determining a threshold in terms of these parameters. We used NEBULA-HL, which is based on a standard h-likelihood method, as a reference to assess the accuracy of NEBULA-LN in terms of mean squared error (MSE).

We first evaluated the performance in a well-designed study in which the numbers of cells were highly homogeneous across the subjects and were assumed to follow a Poisson distribution. We considered the CPS value varying between 100 and 800, and the number of subjects ranging from 30 to 100, which are common settings consistent with empirical observations in droplet-based scRNA-seq data. We simulated counts based on an NBMM with the subject-level overdispersion ${\sigma }^{2}$ ranging from 0.01 to 1 and the cell-level overdispersion ${\phi }^{-1}$ ranging from 0.01 to 100 (A larger ${\phi }^{-1}$ corresponds to higher overdispersion). These ranges were selected because they were close to our estimates observed in the real snRNA-seq data set in ref. ^5^ and the scRNA-seq data set in ref. ^27^. We simulated genes of different mean expression by tuning ${\beta }_{0}$ between −4 and 2 (When the subject-level overdispersion ${\sigma }^{2}$ is small, ${{\exp }}({\beta }_{0})$ approximately equals the counts per cell (CPC) defined by the total counts of a gene divided by the total number of cells). We considered the following two situations, (i) all cells share the same scaling factor and (ii) the scaling factor across cells varied substantially with the CV equal to 1.

**Fig. 2:**
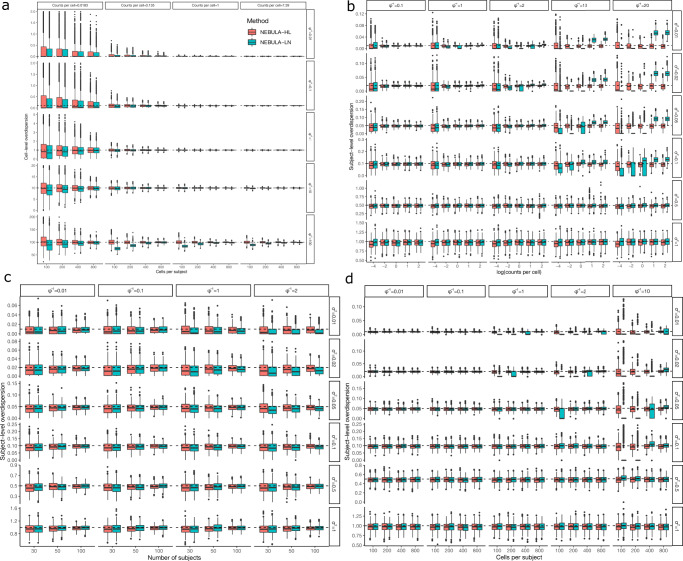
Comparison of estimated cell-level and subject-level overdispersion parameters between NEBULA-LN and NEBULA-HL. **a** The cell-level overdispersion estimated by NEBULA-LN and NEBULA-HL under different combinations of CPS, ${\beta }_{0}$ (a lower ${\beta }_{0}$ corresponding to a lower CPC value), and $\phi$. The number of subjects was set at 50. **b** The subject-level overdispersion estimated by NEBULA-LN and NEBULA-HL under different combinations of ${\beta }_{0}$, ${\sigma }^{2}$, and $\phi$. The number of subjects was set at 50. The CPS value was set at 400. **c** The subject-level overdispersion estimated by NEBULA-LN and NEBULA-HL under different combinations of the number of subjects, ${\sigma }^{2}$, and $\phi$. The CPS value was set at 400, and ${\beta }_{0}$ was set at 0.05. **d** The subject-level overdispersion estimated by NEBULA-LN and NEBULA-HL under different combinations of CPS, ${\sigma }^{2}$, and $\phi$. The number of individuals was set at 50, and ${\beta }_{0}$ was set at 1. The summary statistics were calculated from *n* = 500 simulated replicates in each of the scenarios. The estimates from NEBULA-HL presented in these panels were based on the first-order Laplace approximation, and the results comparing the first-order and higher-order LA methods in NEBULA-HL were presented in Supplementary Fig. S17. CPC: counts per cell. Center line: median. Box limits: upper and lower quartiles.

We found that the MSE of NEBULA-LN for estimating the cell-level overdispersion ${\phi }^{-1}$ decreased uniformly with increasing CPS (Fig. 2a). The convergence of the estimates to their true values supported our theoretical conclusion that NEBULA-LN is asymptotically consistent. Both methods struggled to accurately estimate $\phi$ for low-expression genes as their MSEs increased with the decreasing mean expression, suggesting that genes with a low CPC value could be filtered during the quality control (QC) unless the number of cells is very large. This is because it is difficult to accurately estimate the overdispersions for these genes due to the high sparsity. In most scenarios, NEBULA-LN showed higher MSEs and was asymptotically less efficient than NEBULA-HL for estimating $\phi$. Nevertheless, the MSEs between the two methods were comparable when CPS was >400. When ${\phi }^{-1}$ was very large (=100), NEBULA-LN produced a more biased estimate if CPS is small. A larger CV of the scaling factor showed little impact on the MSE of NEBULA-LN (Supplementary Fig. S2A). Additionally, increasing the number of subjects alone improved the variance, but not the bias of $\hat{\phi }$, suggesting that the bias of $\hat{\phi }$ depended mainly on CPS (Supplementary Fig. S3).

On the other hand, NEBULA-LN showed comparable or even lower MSEs than NEBULA-HL for estimating the subject-level overdispersion ${\sigma }^{2}$ when it is large (${\sigma }^{2}\ge 0.5$) (Fig. 2b). However, when ${\sigma }^{2}$ was small, NEBULA-LN began to produce CPC-dependent biased estimates when ${\phi }^{-1}$ turned larger (Fig. 2b). Increasing CPS alleviated this bias rapidly (Fig. 2d). When the CV of the scaling factor increased from zero to one, an approximately 2-fold increase of ${\rm{CPS}}\cdot \phi$ was required to achieve similar MSEs by comparing Supplementary Figs. S2B, S2D with Fig. 2b, d. These observations suggest that the accuracy of NEBULA-LN for estimating ${\sigma }^{2}$ approximately depends on $\kappa ={\rm{CPS}}\cdot \phi /\left(1+{c}^{2}\right)$ ($c$ is the CV of the scaling factor), which is derived in the Methods section. These empirical results suggest that $\kappa$ determined the lower bound of ${\sigma }^{2}$ for which an accurate estimate could be achieved by NEBULA-LN. Empirically, we found that NEBULA-LN produced good estimates for ${\sigma }^{2}$ as low as 0.02 when $\kappa =200$ (corresponding to the column with ${\phi }^{-1}=2$ in Fig. 2b, c), which was sufficient to reliably test cell-level predictors, as we will show in the next section. Both NEBULA-LN and NEBULA-HL underestimated ${\sigma }^{2}$ particularly when the number of subjects was small (Fig. 2c). We found that this underestimate was not specific to NEBULA, but also present in other tools (e.g., the *glmer.nb* function with nAGQ = 0). Increasing the number of subjects alleviated this bias, and the influence of such bias on testing subject-level preditors will be discussed in the next section.

We further evaluated the performance of NEBULA in unbalanced samples, where the numbers of cells varied substantially across the subjects. An unbalanced sample is common when analyzing a cell subpopulation obtained from clustering. For example, in the snRNA-seq data in ref. ^5^, several individuals had only ~20 inhibitory neurons, although the CPS value among the 48 individuals was ~200. Overall, the results from the unbalanced sample (Supplementary Fig. S4) were comparable to those from the balanced sample (Fig. 2), suggesting the distribution of the number of cells had little impact on estimating ${\sigma }^{2}$ and $\phi$. Overall, NEBULA is able to provide accurate estimates under the parameter regimes relevant for real-data settings and with finite sample sizes.

### NEBULA controls type I error rate

**Fig. 3:**
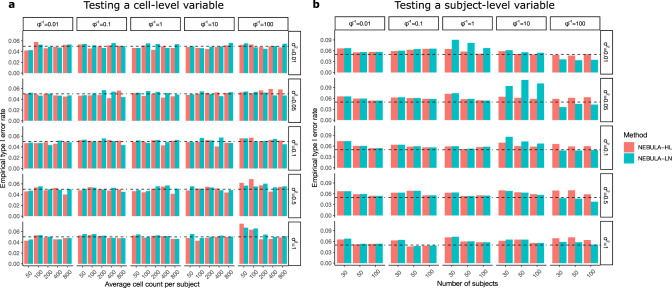
Empirical type I error rate of testing cell-level and subject-level variables using NEBULA-LN and NEBULA-HL. **a** The empirical type I error rate of testing a cell-level variable using NEBULA-LN (blue) and NEBULA-HL (red) under different combinations of CPS, ${\sigma }^{2}$, and $\phi$. The number of subjects was set at 50. **b** The empirical type I error rate of testing a subject-level variable using NEBULA-LN (blue) and NEBULA-HL (red) under different combinations of the number of subjects, ${\sigma }^{2}$, and $\phi$. The CPS value was set at 400. The type I error rate was calculated from *n* = 500 simulated replicates in each of the scenarios and was evaluated at the significance level of 0.05 (the dashed lines). We used the higher-order Laplace approximation in NEBULA-HL for scenarios of low CPC. CPS: cells per subject. CPC: counts per cell.

As shown above, both NEBULA-LN and NEBULA-HL might yield somewhat biased estimates of the overdispersions in specific scenarios. We then assessed the impact of such biases on controlling the type I error rate by testing the fixed-effects predictors under a wide range of settings. As mentioned above, predictors can be classified as either a cell-level or subject-level variable. For a cell-level variable, Fig. 3a shows that both methods controlled the type I error rate well in almost all scenarios. The type I error rate was only slightly inflated when both overdispersions were very large, and the CPS value was as small as 100 (i.e., the bottom-right panels in Fig. 3a). No inflation of the type I error rate was observed for low-expression genes, for which ${\phi }^{-1}$ was more underestimated in NEBULA-LN (Fig. 2a and Supplementary Fig. S5). Besides, the biased estimates of the subject-level overdispersion in NEBULA-LN had little effect on testing a cell-level variable. In a simulation study with very small CPS, we found that noticeable inflation of the type I error rate began to manifest in NEBULA-LN when the CPS value dropped to <30.

In contrast, the type I error rate of testing a subject-level predictor was highly sensitive to a biased estimate of the subject-level overdispersion ${\sigma }^{2}$. The type I error rate in both methods was inflated in the case of 30 subjects and the inflation diminished with the increasing number of subjects (Fig. 3b). This is because both methods underestimated the subject-level overdispersion in this scenario (Fig. 2c). The comparison between Fig. 2c and Fig. 3b suggests that even a small underestimated subject-level overdispersion would lead to an inflated type I error rate of testing a subject-level predictor when the number of subjects is small. Matching Fig. 2b and Supplementary Fig. S6 suggests that NEBULA-LN had inflated or deflated type I error rate of testing a subject-level predictor whenever ${\sigma }^{2}$ was overestimated or underestimated, respectively, even for ${\sigma }^{2}$ being as low as 0.01. Besides, we found that misspecification of the random effects could lead to an inflated type I error rate as well, particularly if the sample size is small. These results suggest that NEBULA or other methods based on a mixed model should be used with caution when testing a subject-level predictor, particularly when the number of subjects is small (<100).

We also evaluated the performance of a Poisson mixed model for testing a subject-level predictor. If we ignore the cell-level overdispersion ${\phi }^{-1}$ in NEBULA, we end up with a Poisson gamma mixed model (PGMM)^28^, the likelihood function of which has a simple analytical form. Therefore, it does not suffer from the problem of underestimating ${\sigma }^{2}$ as in NEBULA-HL and is computationally much faster than the Poisson mixed model estimated by *glmer.nb* with nAGQ=10 adopted in ref. ^5^. As the cell-level overdispersion is ignored in the PGMM, it definitely cannot be used to test a cell-level predictor. However, it is still interesting to check its type I error rate when used for testing a subject-level predictor. Supplementary Fig. S7 shows that overall the PGMM controlled the type I error rate well in scenarios where the number of subjects was 100, except for situations where both the cell-level and subject-level overdispersions were large (${\phi }^{-1}\ge 100$ and ${\sigma }^{2}\ge 0.5$). Such situations account for a very small fraction of genes (usually low-expression genes) in real data and such genes are often excluded during quality control. For instance, we observed <0.1% genes with CPC > 0.1% having ${\phi }^{-1} > 100$ in the major cell types in the snRNA-seq data in ref. ^5^, and the percentage became almost zero when we filtered out lowly expressed genes (CPC < 1%) during quality control. Therefore, the PGMM might be considered as a fast tool for testing subject-level predictors followed by a second-round sensitivity analysis for top signals if the computational intensity is a major concern.

Based on these simulation results, the strategy that we recommend for testing the fixed-effects predictors, as summarized in Supplementary Data 1, is to fit the data first using NEBULA-LN to obtain initial estimates $\hat{\phi }$ and ${\hat{\sigma }}^{2}$. Both overdispersion parameters are re-estimated using NEBULA-HL if ${\rm{CPS}}\cdot \hat{\phi } < 20$ or ${\rm{CPS}} < 30$. Otherwise, we fix $\hat{\phi }$ and only use NEBULA-HL to re-estimate ${\sigma }^{2}$ if $\hat{\phi } \,<\, \kappa \left(1+{\hat{c}}^{2}\right)/{\rm{CPS}}$ and ${\hat{\sigma }}^{2} < \frac{8\left(1+{\hat{c}}^{2}\right)}{{\rm{CPS}}\cdot \hat{\phi }}$, where $\hat{c}$ is the CV estimated from the data and we chose $\kappa =200\;{\rm{and}}\;800$ for testing a cell-level and subject-level predictor, respectively, based on the simulation results. Otherwise, we report the estimates directly from NEBULA-LN because in this case, NEBULA-LN produces a reliable estimate for a sufficiently small ${\sigma }^{2}$. We used this strategy in our following analysis of the real data sets. In the snRNA-seq data set generated by^5^, ~90% and ~80% of genes with CPC > 0.1% were analyzed using NEBULA-LN alone under this strategy in the excitatory neurons (CPS = ~700) and oligodendrocytes (CPS = ~380). We found that ${\hat{c}}^{2}$ of the cell library size was ~0.8 for the neurons and ~0.25 for all other cell types. The larger CV in the neurons was probably because the neurons consisted of highly heterogeneous subpopulations (Supplementary Fig. S8).

### Subpopulation heterogeneity and known covariates contribute to overdispersions

The subject-level and cell-level overdispersion parameters likely reflect some intrinsic biological features of a gene. A gene with a larger subject-level overdispersion might indicate that the gene is strongly regulated by subject-level factors such as age, sex, disease status, exposure, and genetic variation. We used NEBULA to dissect the overdispersions of genes in the snRNA-seq data in ref. ^5^. We carried out the analysis in each of the six major cell types, including excitatory neurons, inhibitory neurons, oligodendrocytes, astrocytes, oligodendrocyte progenitor cells (OPCs), and microglia.

**Fig. 4:**
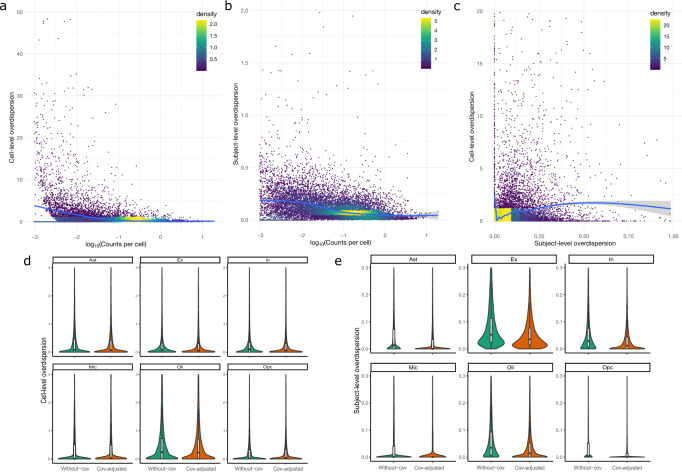
Decomposed cell-level and subject-level overdispersions in major neural cells. **a** The cell-level overdispersion and **b** the subject-level overdispersion of 16,207 genes in the excitatory neurons estimated by NEBULA across different CPC values. No covariates other than the intercept were included in the model. The color indicates the two-dimensional kernel density computed using the *kde2d* R function with *n* = 100. **c** The cell-level overdispersion of 16,207 genes in the excitatory neurons estimated by NEBULA versus their subject-level overdispersion. **d** The estimated cell-level overdispersion and **e** the estimated subject-level overdispersion in each of the six brain cell types before and after adjusting for age, sex, race, AD status, the total number of features of the cell, and the percentage of ribosomal genes. Only genes with CPC > 0.1% in the relevant cell type were included in the above results. The cells in this analysis are from the 48-subject snRNA-seq data set^5^ in the frontal cortex.

We observed that the mean cell-level overdispersion grew steadily with lower mean gene expression in the excitatory neurons (Fig. 4a) and also in the other cell types (Supplementary Fig. S9). This mean-overdispersion trend is reminiscent of that observed in bulk RNA-seq data, in which low-expression genes often show higher variance^6^. Less than 0.1% of genes in the excitatory neurons had an estimated cell-level overdispersion ${\hat{\phi }}^{-1}$ larger than 100, and all of them were low-expression genes. In contrast, the subject-level overdispersion exhibited a similar mean-overdispersion trend, except that the overdispersion leveled off for abundantly expressed genes with CPC>1 in the excitatory neurons (Fig. 4b). Similar patterns were observed in the other cell types (Supplementary Fig. S10). We observed a higher variation of both estimated overdispersions in lower-expression genes, which is due to a larger standard error for those genes as shown in the simulation (Fig. 2). There was no clear correlation between the subject-level and the cell-level overdispersions (Fig. 4c).

We then explored which factors might contribute to the gene-specific overdispersions in each of the cell types. First, we found that the median cell-level overdispersion dropped markedly in the different subpopulations within a given cell type (Supplementary Fig. S11A), suggesting that differential expression between subpopulations was one of the major sources for the cell-level overdispersion. In contrast, the median subject-level overdispersion in the subpopulations dropped only mildly (Supplementary Fig. S11B). We then investigated subject-level covariates, including age, sex, AD diagnosis, race, and cell-level covariates, including the number of total features (i.e., detected genes) of a cell and the percentage of counts mapping to ribosomal protein genes. NEBULA enables readily exploring the role of such covariates by including them in the regression model. We found that the overall subject-level overdispersion dropped substantially in most of the cell types after adjustment for these covariates, but the cell-level overdispersion remained almost at the same level (Fig. 4d, e). These results suggest that the subject-level overdispersion was largely attributed to these factors, while the cell-level overdispersion resulted from subpopulation heterogeneity and other unknown factors, e.g., drop-out rates.

In terms of models, the estimated cell-level overdispersions were highly comparable between NEBULA and *glmer.nb* (Supplementary Fig. S12), except for an outlier in *glmer.nb*. The estimated subject-level overdispersions were also strongly correlated for those genes with a small to moderate subject-level overdispersion (Supplementary Fig. S13), although it is not straightforward to compare the absolute values because the random effects in these two NBMMs are assumed to follow slightly different distributions (see the Methods section). The larger difference between NEBULA and *glmer.nb* observed in larger subject-level overdispersions was expected, as previously shown that the two models are highly consistent when the subject-level overdispersion is small^28^.

### NEBULA accurately identifies cell-type marker genes

**Fig. 5:**
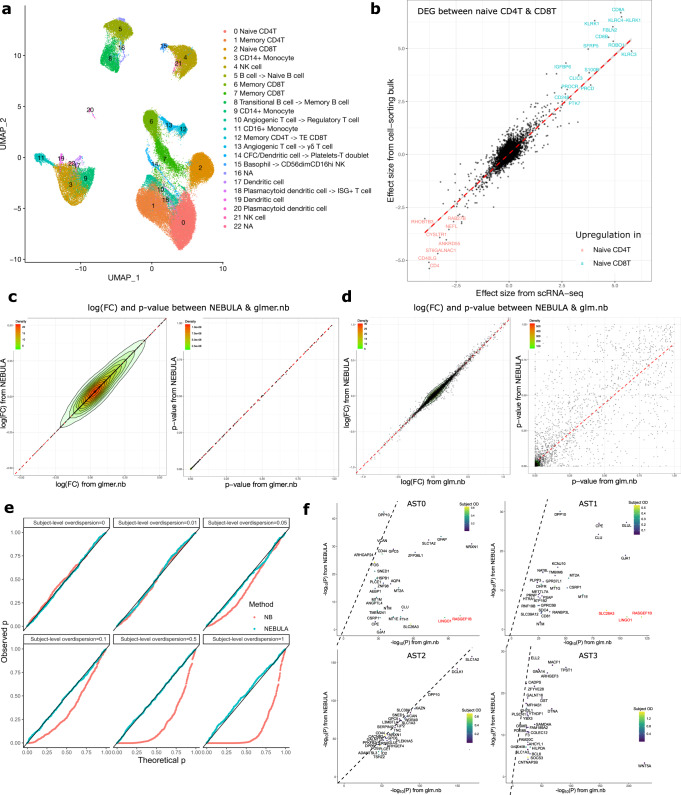
Identification of cell marker genes using NEBULA. **a** A UMAP plot of the cells in the PBMC scRNA-seq data^27^. The automatic cell-type annotation was performed using NEBULA and scCATCH^29^. The corrected or refined cell type by the manual annotation was shown after the arrow. Cluster 16 and 22 were not annotated because of either too few cells or marker genes. **b** Comparison between log(FC) estimated by NEBULA in the scRNA-seq data^27^ and the effect size estimated by *glm.nb* in the cell-sorting bulk RNA-seq data^30^ in the analysis of differentially expressed genes between naive CD8+ and CD4+ T cells. Genes having both log(FC) and an effect size >2.5 were highlighted in blue for naive CD8+ and red for naive CD4+ T cells. Comparison between the log(FC) and *p*-values from **c** NEBULA and *glmer.nb* and **d** NEBULA and *glm.nb* in the differential expression analysis of detecting marker genes for Cluster 1 (memory CD4+ T cells) in the scRNA-seq data^27^. **e** Q–Q plots of *p*-values for testing the association between simulated counts and cell clusters using NEBULA and a simple negative binomial regression (shown as NB in the plot). The counts were simulated under the null model (i.e., no association between the counts and cell clusters) based on an NBMM with the subject-level overdispersion (${\sigma }^{2}$) ranging from 0 to 1 and a cell-level overdispersion fixed at 1. **f** Comparison of the *p*-values of the top 30 genes identified by *glm.nb* associated with each of the four astrocyte subpopulations (AST0-AST3) in the snRNA-seq data^5^ with those reported by NEBULA. The labels of the four astrocyte subpopulations are corresponding to those presented in ref. ^5^. Subject OD: the subject-level overdispersion estimated by NEBULA. Genes highlighted in red showed substantially reduced significance in NEBULA compared to the *p*-values in the simple negative binomial regression.

To demonstrate that NEBULA is an efficient tool to find marker genes for annotating cell clusters, we next analyzed a recent multi-subject peripheral blood mononuclear cell (PBMC) scRNA-seq data set^27^. The PBMC scRNA-seq data contain 69,516 cells from the peripheral blood of 11 subjects. We first classified the cells into 22 clusters using Seurat (Fig. 5a). The cells within each cluster were distributed almost evenly across the subjects. We performed a differential expression analysis using NEBULA for each of the clusters to identify marker genes that showed significantly higher expression in one cluster than the cells of the other clusters. We then used the top marker genes (see the Methods section for the selection of the marker genes) as the input to scCATCH^29^ to annotate these clusters. We found that this automatic pipeline produced consistent results for the major cell types in PBMC compared to our refined manual annotation (Fig. 5a). Discrepancies appeared mainly in the annotation of subcell types in T cells due in large part to the low resolution of the T-cell subpopulations in the reference databases in scCATCH. To validate the differentially expressed genes (DEGs) identified by NEBULA from the scRNA-seq data, we compared the effect sizes from the DEG analysis between the naive CD4+ T cell (cluster 0) and the naive CD8+ T cell (cluster 2) with those obtained from a reference cell-sorting bulk RNA-seq data from the Database of Immune Cell Expression (DICE)^30^. We found that the estimated effect sizes were highly concordant between these two independent data sets and different models (Fig. 5b).

To compare different NBMMs, we found that the estimated effect sizes and p-values in the 11-subject PBMC scRNA-seq data were highly consistent between NEBULA and the NBMM using *glmer.nb* (Fig. 5c) while NEBULA was faster by orders of magnitude. We also compared the performance in the 48-subject snRNA-seq data. Estimated fixed effects of a subject-level variable were also consistent between NEBULA and *glmer.nb* (Supplementary Fig. S14), although the consistency was lower than that of a cell-level variable (Fig. 5c). In contrast, many genes showed very different *p*-values between NEBULA and a naive negative binomial model (NBM) using *glm.nb* (Fig. 5d). The difference can be justified by the fact that using a negative binomial regression without the subject-level random effects could result in substantially inflated false positives^20^. Disproportionate cell frequency among individuals may exacerbate the inflation of spurious associations, similar to Simpson’s Paradox^31^. In this case, subjects can be a confounding factor between gene expression and cell clusters. On the other hand, including subjects as fixed effects would lead to too many parameters in the model, especially when the number of subjects is large. Therefore, an NBMM is an ideal approach for the identification of marker genes for subpopulations in multi-subject scRNA-seq data.

To illustrate this problem, we used the 3386 astrocytes from the snRNA-seq data in the frontal cortex, which were classified into four subclusters (AST0-AST3). Some of the subclusters were distributed across the subjects in a highly unbalanced pattern. For example, the vast majority of the cells in the AST3 cluster came from only four subjects (Supplementary Fig. S15). We simulated the counts of gene expression using an NBMM under the null hypothesis (i.e., not associated with any of the subclusters) with both subject-level and cell-level overdispersions. We assessed the false-positive rates (FPR) under four scenarios with the variance component of the subject-level overdispersion ranging from 0 to 0.1. As we can see in Fig. 5e, even with the subject-level overdispersion ${\sigma }^{2}$ as small as 0.05, the FPR started to show inflation. The inflation rose rapidly with the increased subject-level overdispersion. In contrast, NEBULA controlled the FPR very well.

To evaluate how this inflation of FPR might affect the selection of marker genes, we carried out an association analysis between the gene expression and the four subclusters in the astrocytes in the snRNA-seq data^5^ using a negative binomial regression and NEBULA. Among the top 30 genes identified by a negative binomial regression, most of the p-values were consistent between the two methods in the subclusters AST2 and AST3. However, some of the top genes in the subclusters AST0 and AST1, such as *RASGEF1B* and *LINGO1*, were much less significant in NEBULA than in the negative binomial regression (Fig. 5f). Both genes showed uniformly strong subject-level overdispersion in the astrocytes (Fig. 5f) and the other five cell types, indicating that the significant *p*-values in the negative binomial regression could result from failing to account for the subject-level overdispersion. Furthermore, an independent cell-type-specific bulk RNA-seq data^32^ showed that the expression of *RASGEF1B* and *LINGO1* was much lower in astrocytes than that in microglia and oligodendrocytes, respectively. Given the evidence, care should be taken when classifying these genes as subcell-type marker genes based on the results from the negative binomial regression without the random effects. Besides, *GJA1* was identified by NEBULA as a marker gene of AST1 but not AST0, while a negative binomial regression identified *GJA1* as significant in both subclusters (Fig. 5f). Thus, NEBULA provides a principled way to identify genes with preferential expression patterns in cellular subpopulations that are consistent across individuals, by effectively accounting for subject-level random effects.

### NEBULA robustly estimates cell-level co-expression

Estimating co-expression between genes is crucial for inferring functional associations among genes and defining molecular pathways and coherent gene modules. However, co-expression patterns estimated from bulk RNA-seq data are heavily affected by many hidden confounders such as cell type composition and experimental noise. In contrast, scRNA-seq data opens up new possibilities for investigating co-expression at the cellular level. We interpret the cell-level co-expression as the co-occurrence of two genes in a cell. Nevertheless, we will show that co-expression analysis of scRNA-seq data using common methods poses special challenges. NEBULA has unique advantages to explore cell-level co-expression over common distance metrics such as Pearson or Spearman correlation coefficient because it is more robust and adjusts for overdispersions, sequencing depth, and covariate effects.

To give a demonstration of these advantages and their potential to explore specific biological problems, we compared the performance of estimating cell-level co-expression of *TCF7* and *BCL3*, two of the major transcription factors in adaptive immune cells, with 14,770 genes having CPC > 0.05% in the memory CD4^+^ T cells in the PBMC scRNA-seq data. We evaluated four methods and assessed their difference including (i) NEBULA without adjusting for any confounders, (ii) NEBULA adjusting for total features, percentage of mitochondrial genes, and percentage of ribosomal genes, (iii) Pearson’s correlation of normalized expression, (iv) Spearman’s rank-order correlation of normalized expression. In bulk RNA-seq data, the Spearman correlation is often preferable to the Pearson correlation because it is more robust against outliers. We found that NEBULA and Pearson’s correlation produced more concordant results with each other than the Spearman correlation in both analyses (Supplementary Data 2). We observed many biological related genes such as *LEF1* and *CCR7* with *TCF7* and *NFKBIA* and *NFKB2* with *BCL3* among the top co-expressed genes, respectively. In contrast, the Spearman correlation yielded problematic results. For example, many biologically irrelevant and very low-expression genes such as *AC124068.2* showed very significant p-values associated with *TCF7*. Additionally, in the analysis of *BCL3*, which was less abundantly expressed than *TCF7*, 11,821 out of the 14,770 genes had a significant *p*-value < 1E-7, suggesting strong inflated FPRs using the Spearman correlation. This issue probably resulted from the excessive zeros for the low-expression genes, which led to too many ties in both variables. On the other hand, the Pearson correlation was extremely sensitive to outliers. For example, *CX3CR1* and *LINC02446* showed very significant p-values (p<2E-7) with *BCL3* using the Pearson correlation but were only nominally or even not significant by using NEBULA (Supplementary Data 2). We found that the significant *p*-value from the Pearson correlation was completely driven by only one outlier cell and it became non-significant after removing this cell (Supplementary Fig. S16). In contrast, NEBULA showed very robust and more accurate co-expression estimates. Furthermore, through the adjustment of these confounders, NEBULA eliminated most ribosomal genes from the top list and thus prioritized more biologically relevant genes (Supplementary Data 2).

### Cell-level co-expression analysis of *APOE*

By utilizing NEBULA, we focused on identifying the genes the expression of which were correlated with the expression of *APOE*, the strongest genetic risk factor for AD, at the single-cell level. As *APOE* is abundantly expressed only in astrocytes and microglia cells, we performed the analysis in these two cell types using two large-scale snRNA-seq data sets in the frontal cortex from the ROSMAP, which included 48 and 32 subjects from an elderly non-Hispanic white population, respectively. *APOE* is known to affect multiple cellular functions and molecular processes in an isoform-specific manner^33^. The *APOE* gene is primarily present in the form of one of three main alleles *APOE e3*, the most common allele; *APOE e2*, the less frequent and underrepresented in AD-affected subjects, and *APOE e4*, the relatively frequent and highly overrepresented in AD-affected subjects. Therefore, *APOE e2* and *APOE e4* are usually considered as, respectively, having a protective or a risk-amplifying role in AD. The molecular and cellular mechanism through which such risk effects are mediated is not understood. One way by which *APOE* could contribute to such effects is by exerting a differential influence on molecular pathways relevant to the biology of different cell types. NEBULA enables the empirical exploration of this hypothesis by testing (1) whether *APOE* presents selective functional interaction partners in different cell types, and (2) whether such selection is also influenced by having a given allele. Our results support both possibilities.

**Fig. 6:**
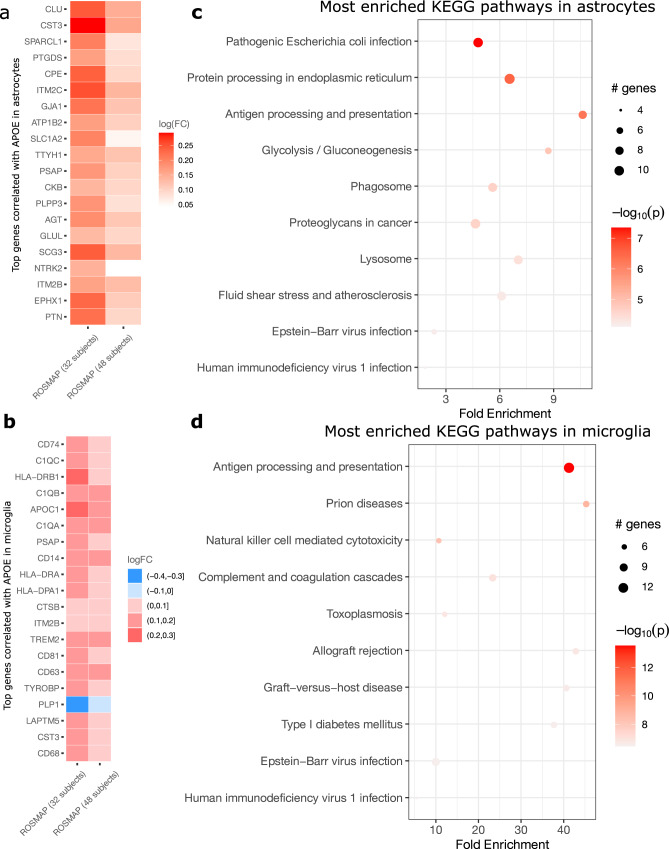
Analysis of cell-level co-expression of *APOE* in astrocytes and microglia. **a** and **b** Top 20 genes whose expression in astrocytes (panel a) and microglia (panel **b**) were correlated with the expression of *APOE* at the single-cell level. The correlation measured by log(FC) was obtained by NEBULA in two snRNA-seq data sets^5,78^ in the frontal cortex from the ROSMAP. **c** and **d** Top 10 KEGG pathways in which the genes most correlated with *APOE* in astrocytes (panel **c**) and microglia (panel **d**) were enriched. The size of the circles indicates how many genes were found in that pathway. The color indicates the *p*-value of the enrichment.

Genes that are highly correlated with the *APOE* expression are reproducible across the subjects and data sets (Supplementary Data 3). In both data sets, the *APOE* expression in astrocytes was most strongly correlated with *CLU*, which is also a genetic risk factor for AD^34^, on top of other AD-related genes such as *CST3*^35^ (Fig. 6a). In contrast, *APOE* was co-expressed in microglia with multiple immune-related genes, particularly *TREM2, TYROBP*, and members of the complement system including *C1QA*, *C1QB*, and *C1QC* (Fig. 6b), all of which have been implicated in microglia response to amyloid pathology^36^. We also found examples of recurrent association across cell types. *ITM2B*, an inhibitor of the amyloid-beta peptide aggregation, was strongly co-expressed with *APOE* in both cell types, consistent with the role of *APOE* in both glial cell types in amyloid-beta clearance^33^. The pathway enrichment analysis revealed that the top co-expressed genes were enriched for pathways involving antigen processing and presentation, prion disease, and the complement system in microglia (Fig. 6d), while *APOE*-associated pathways in astrocytes included protein processing in the endoplasmic reticulum, and antigen processing and presentation (Fig. 6c), again suggesting both recurrent and cell-type-specific processes mediated by distinct *APOE* molecular partners. It should be noted that the identified co-expression genes were based on the within-subject expression, thus ruling out effects from subject-level confounders such as AD status. The pathways identified in such a way could be used in downstream analyses to test whether the presence of AD pathology increases or decreases their activity, thus providing a more directed approach as compared, for example, to the conventional GO enrichment analysis, which often leads to insignificant results due to overtesting.

Finally, to test the possibility of an allele-specific influence on APOE co-expression, in addition to cell-type specificity, we carried out an isoform-specific co-expression analysis by dividing cells into *APOE e3e3*, *APOE e4*^+^, or *APOE e2e3* genotype groups. The co-expression patterns showed substantial differences among the isoform groups in microglia, but not in astrocytes (Supplementary Data 4). Particularly, strong co-expression was observed in the *APOE e2e3* cell population with *CST3*, and the three genes (*C1QA*, *C1QB*, *C1QC*) in the complement system, but not with *TREM2* (Supplementary Data 5), which might suggest different biological mechanisms in microglia behind these two *APOE* isoforms. These preliminary analyses demonstrate how NEBULA can be used to both produce and test hypotheses about gene function in a cell type and isoform-specific fashion, which could complement more in-depth experimental studies.

## Discussion

In this work, we propose a fast NBMM for the association analysis of large-scale multi-subject single-cell data. We evaluated and demonstrated the performance of NEBULA using a comprehensive simulation study and multiple droplet-based multi-subject single-cell data sets in the human frontal cortex and PBMC. Overall, our results show that combining methods based on the approximated marginal likelihood and the h-likelihood, NEBULA managed to achieve considerable speed gain and also practically preserve estimation accuracy for analyzing scRNA-seq data. We expect that NEBULA can also be appropriate for other single-cell data such as scATAC-seq data.

The asymptotic efficiency and estimation accuracy of NEBULA-LN are primarily determined by CPS and the cell-level overdispersion. Specifically, our results suggest that $\phi \cdot {\rm{CPS}} \,> \, 20$ was sufficient to produce a sufficiently accurate estimate of the cell-level overdispersion. This means that genes with, e.g., a cell-level overdispersion <10 in a single-cell data set with ~200 cells per subject can benefit from NEBULA-LN. This criterion covers the vast majority of genes in many large-scale real data. On the other hand, a larger CPS value enables a more accurate estimate for a smaller subject-level overdispersion. Accurate estimation requires a larger $\phi \cdot {\rm{CPS}}$ for the subject-level overdispersion than the cell-level overdispersion and also depends on the CV of the scaling factor and the contribution from the cell-level variables. Compared to a standard h-likelihood method, we see that NEBULA-LN sacrifices some asymptotic efficiency in estimating the cell-level overdispersion, which means that more cells and counts per cell are needed to achieve the same MSEs.

NEBULA is robust in controlling the FPR for testing cell-level variables such as subpopulation marker genes. The simulation study suggests that although the estimated overdispersions for low-expression genes were often less accurate, there was little influence on the FPR. It is possible to treat subjects as batch effects and regress them out in identifying marker genes, but this approach would compromise statistical power if the number of subjects is large. NEBULA is a powerful tool for robustly identifying marker genes. On the other hand, our simulation study suggests that testing a subject-level predictor is sensitive to the estimate of the subject-level overdispersion if the number of subjects is small, which is consistent with a previous simulation study involving only three plates (conceptually equivalent to the subjects)^37^. We further noticed that when the number of subjects is small, misspecification of the distribution of the random effects may also lead to inflated FPR in testing a subject-level predictor. The downward bias of NEBULA-HL in estimating the subject-level overdispersion is in line with theoretical results for binary data^38^ and count responses^39^ for NBLMM. This bias is more severe in the NBGMM and genes having very low expression and a large subject-level overdispersion because the h-likelihood is highly skewed when the response is sparse. The efficient higher-order LA method that we developed substantially alleviated this issue. If the computational efficiency is a major concern, we found that using the PGMM followed by a sensitivity analysis for top findings could be a fast and viable option for testing a subject-level predictor. Another strategy for testing a subject-level variable is to pool all cells of each subject and treat the data as a bulk RNA-seq data set (i.e., the pseudo-bulk method^37,40^). One potential disadvantage of this strategy is that it cannot adjust for cell-level covariates such as mitochondrial and ribosomal gene concentration to increase the statistical power. The pseudo-bulk method might also be underpowered when the CPS values are imbalanced across the subjects^41^.

The trend of higher average cell-level overdispersion in low-expression genes is in line with those reported in bulk RNA-seq data^6,7^. A justification for this observation unique to scRNA-seq data can be that low-expression genes might have a higher drop-out rate or have larger variation across subpopulations. In bulk RNA-seq data, a large proportion of overdispersion results from heterogeneous cell type composition across the individuals. While this heterogeneity is much better controlled in the scRNA-seq data, subpopulation composition still accounts for most of the cell-level overdispersion. The observed smaller cell-level overdispersion in abundant genes might be attributed to a lower drop-out rate and stable expression across subpopulations. In contrast, the subject-level overdispersion is explained by many known covariates. With increased gene expression, the cell-level overdispersion drops while the subject-level overdispersion stabilizes. More work is needed to examine to what extent the subject-level overdispersion is explained by the effects of *cis*-eQTLs and whether eGenes (i.e., genes having ≥1 eQTL) show a larger subject-level overdispersion.

The co-expression of *APOE* in astrocytes and microglia is intriguing. Among the top co-expressed genes, multiple SNPs in *CLU* and *TREM2* are AD GWAS loci^34,42–44^. *CLU, CST3*, and *ITM2B* are involved in regulating beta-amyloid production or its fibril formation^45–49^. *CST3* and *ITM2B* are culprits implicated in hereditary cerebral amyloid angiopathy^50^. The co-expression results in astrocytes suggest that *APOE* might be involved in the same biological pathway of these genes regulating beta-amyloid production. On the other hand, the co-expression results in microglia support a recent finding^51^ that *APOE* is involved in the immune system through the complement system, particularly C1q. We further found that this co-expression pattern is *APOE2*^*+*^ and *APOE4*^*+*^ dependent in microglia, which might provide more insights into the different roles of *APOE* isoforms in AD. These results suggest that the effect of *APOE* on AD is related to regulating both beta-amyloid deposition and the immune system. It should be noted that, because of the data sparsity in the scRNA-seq data, a drawback of this co-expression analysis is that there is little statistical power to identify low-expression genes because the counts of these genes have very small variation. Therefore, no observed correlation does not necessarily mean that the two genes are not co-expressed. Interpretation of the co-expression results for low-expression genes should be cautious. Overall, our analyses of *APOE* co-expression demonstrate how NEBULA can be used as a tool to explore gene function in a cell type and isoform-specific fashion or to identify modules of co-regulated genes while correcting for subject-level cofounders. Both of these approached can complement more in-depth experimental studies to mechanistically dissect the robust observation detected in human tissue.

## Methods

### The model specification in NEBULA

Consider a raw count matrix of $n$ cells from $m$ individuals in a single-cell data set. We choose to use an NBM to take into account the uncertainty originating from the Poisson sampling process, biological effects, and technical noise. Denote by ${y}_{{ij}}$ the raw count of a gene and by ${{\boldsymbol{x}}}_{{ij}}=({x}_{{ij}0},\ldots ,{x}_{{ijk}})\,{{\in }}\,{{\mathbb{R}}}^{1{{\times}}{(}k+1)}$ a row vector of $k$ fixed-effects predictors and the intercept (${x}_{{ij}0}=1$) of cell $j$ ($j\in \{1,\ldots ,{n}_{i}\}$ and ${\sum }_{i}{n}_{i}=n$) in individual $i$ ($i\in \{1,\ldots ,{m\}}$). We model the raw count using the following NBMM

1 $${y}_{{ij}} \sim {{\mathrm{NB}}}({\mu }_{{ij}}={\pi }_{{ij}}{{\exp }}({{\boldsymbol{x}}_{ij}}^{T}{\boldsymbol{\beta }}\,{\boldsymbol{+}}\,{{\log }}({\omega }_{i})),\phi ),$$

where ${{\mathrm{NB}}}$ is a negative binomial distribution parametrized by its mean $\mu$ and cell-level dispersion parameter $\phi$ such that the variance is $\mu +{\mu }^{2}/\varphi$, ${\boldsymbol{\beta }}\,{\boldsymbol{\in }}\,{{\in }}\,{{\mathbb{R}}}^{{\boldsymbol{(}}k{\boldsymbol{+}}{\mathbf{1}}{\boldsymbol{)}}{\boldsymbol{\times }}{\mathbf{1}}}$ is a vector of the coefficients of the predictors, ${\omega }_{i}$ are subject-level random effects capturing the between-subject overdispersion, and ${\pi }_{{ij}}$ is a known cell-specific scaling factor reflecting the sequencing depth, which can be the library size or some global normalizing factor (e.g., ref. ^52^). Note that ${\pi }_{{ij}}$ can also be gene-specific as recent studies show that a single normalizing factor might be insufficent to normalize scRNA-seq data^53^. The negative binomial log-linear mixed model (NBLMM) proposed in ref. ^54^ assumes a log-normal distribution for the random effects ${\omega }_{i}$, i.e.,

2 $${\omega }_{i} \sim {{\mathrm{lognormal}}}\left(0,{\sigma }^{2}\right)\;{\rm{or}}\;{\rm{In}}({\omega }_{i}) \sim N(0,{\sigma }^{2}),$$

where ${\sigma }^{2}$ is the variance component of the between-subject random effects.

The estimation of the NBLMM poses a computational challenge because the marginal likelihood is intractable after integrating out the random effects ${\omega }_{i}$. Booth et al.^54^ originally propose an estimation method based on an Expectation-Maximization (EM) algorithm. As the NBLMM falls in the framework of the GLMM, conventional estimation methods for the GLMM can also be applied, including penalized likelihood-based methods^12,13^, Laplace approximation^16^, AGQ^24,25^. These methods, implemented in the *lme4* R package^18^ through the argument *nAGQ*, differ in how to approximate the high-dimensional integral in the marginal likelihood, among which the penalized likelihood-based method is the fastest but the least accurate while AGQ is more accurate and much slower. We refer to ref. ^55^ for more extensive reviews of these methods. More recently, Zhang et al.^17^ propose an estimation procedure for the NBLMM by transforming the problem into repeatedly fitting a linear mixed model using iteratively reweighted least square. All these methods involve at least a two-layer optimization procedure. Although the time complexity is ${\mathscr{O}}(n)$ under the assumption of the independent random effects, the two-layer procedure often requires hundreds of iterations to converge, which becomes the major computational bottleneck. On the other hand, Bayesian methods such as MCMC^26^ offer better accuracy, but are even slower than the above methods.

The rationale behind NEBULA is to skip the time-consuming two-layer optimization algorithm for as many genes as possible and to substantially reduce the number of iterations if such an algorithm has to be applied. The key idea is to derive an approximate marginal likelihood in a closed-form so that the model can be estimated using a simple Newton-Raphson (NR) or quasi-Newton method. Inspired by ref. ^28^, instead of the log-normal distribution in eq. (2), we assume that the between-subject random effects follow the following gamma distribution

3 $${\omega }_{i} \sim {\mathrm{Gamma}}\left(\alpha ,\lambda \right),$$

$$\alpha =\frac{1}{{\mathrm{exp}}\left(\sigma^{2}\right)-1},$$

$$\lambda =\frac{1}{{\mathrm{exp}}\left(\sigma^{2}/2\right)({\mathrm{exp}}\left(\sigma^{2}\right)-1)}$$

where $\alpha$ and $\lambda$ are the shape and rate parameters, respectively. One advantage of this parametrization is that it matches the first two moments to the NBLMM, so that even in a situation where eq. (2) is the true distribution of the random effects, Eq. (3) can still provide an accurate estimate of ${\sigma }^{2}$ when it is not large^28^. We call the NBMM with the random effects defined in Eq. (3) as a negative binomial gamma mixed model (NBGMM). Under the NBGMM, the marginal mean and variance of ${y}_{{ij}}$ are given by

$$E\left(y_{ij}\right)= \pi_{ij} {\mathrm{exp}} \left({{\boldsymbol{x}}_{ij}}^{T} {\boldsymbol{\beta }} + \frac{\sigma^{2}}{2}\right),$$

4 $${\mathrm{Var}}\left(y_{ij}\right) = E\left(y_{ij}\right) + E^{2}\left(y_{ij}\right)\left({\mathrm{exp}}\left(\sigma ^{2}\right)-1\right) + E^{2}\left(y_{ij}\right)\frac{{\mathrm{exp}}\left(\sigma^{2}\right)}{\phi },$$

from which we see that the first overdispersion term in Eq. (4) is controlled by the between-subject variance component ${\sigma }^{2}$ alone, and the other includes both ${\sigma }^{2}$ and $\phi$ (The derivation can be found in Supplementary Note A.1). Previous studies^20,56,57^ indicate that it has practically little influence on the estimate of fixed-effects predictors, particularly at the cellular level, to assume a log-normal, gamma, or even misspecified distributions for the random effects. Nevertheless, misspecified random-effects distribution has a larger impact on the estimate of subject-level predictors if the number of subjects is small. Our real data analysis (Fig. 5c and Supplementary Fig. S14) showed concordant results with these studies. In general, the marginal likelihood of the NBGMM after integrating out ${\omega }_{i}$ cannot be obtained analytically. However, we show in the next section how to achieve an approximated closed form of the marginal likelihood when ${n}_{i}$ is large. We first focus on the derivation in an asymptotic situation, and then investigate its practical performance in a finite sample.

### Approximation of marginal likelihood in NEBULA-LN

We first decompose the NBGMM defined by Eq. (1) and Eq. (3) into a mixture of Poisson distributions as follows

5 $$y_{ij} \sim {\mathrm{Poisson}}\left(\omega_{i} \upsilon_{ij} {\mathrm{exp}} \left({\boldsymbol{x}}_{ij}^{T} {\boldsymbol{\beta }}+ {\mathrm{log}} (\pi_{ij})\right)\right),$$

$$\omega_{i} \sim {\mathrm{Gamma}}\left(\alpha ,\lambda \right),\quad$$

$$\upsilon_{ij} \sim {\mathrm{Gamma}}\left(\phi ,\phi \right),\quad$$

where ${\upsilon }_{{ij}}$ has the mixing gamma distribution. We then switch the order of the integral in the marginal likelihood to first integrate out ${\omega }_{i}$. The PGMM proposed in ref. ^28^, which has a closed-form likelihood, is a special case of the NBGMM if we assume ${\upsilon }_{{ij}}=1$ in Eq. (5). As ${\omega }_{i}$ is a conjugate random effect for the Poisson model^58^, a partial marginal likelihood after integrating out ${\omega }_{i}$ for the cells in individual $i$ can be expressed explicitly as

$$L_{i}\left({\boldsymbol{\beta }}, \sigma^{2}, \phi \right) = \iint_{0}^{+\infty}\mathop{\prod }\limits_{j=1}^{{n_{i}}}f\left(y_{ij}, |, \omega_{i}, {\boldsymbol{\upsilon}}_{i}\right)f\left(\omega_{i}\right)f\left({\boldsymbol{\upsilon}}_{i}\right)d\omega _{i} d {\boldsymbol{\upsilon}}_{i}$$

6 $$= \, \frac{\lambda^{\alpha }}{\mathop{\prod}\limits_{j}y_{ij}}\frac{{\boldsymbol{\Gamma }}\left(\mathop{\sum}\limits_{j}{y}_{ij} + \alpha \right)}{{\boldsymbol{\Gamma}}\left(\alpha\right)}{\mathrm{exp}}\left(\mathop{\sum}\limits_{j=1}^{{n_{i}}}{y}_{ij}\left({x_{ij}}^{T}{\boldsymbol{\beta }} + {\mathrm{log}}\left(\pi_{ij}\right)\right)\right)\int_{0}^{+\infty }\left(\mathop{\prod}\limits_{j}{\upsilon_{ij}}^{{y_{ij}}}\right)\\ \, \underbrace{\left(\lambda +\mathop{\sum}\limits_{j}\left(\upsilon_{ij}{\mathrm{exp}}\left({{{\boldsymbol{x}}}_{{ij}}}^{T}{\boldsymbol{\beta}}+{{\log}}\left({\pi }_{{ij}}\right)\right)\right)\right)^{-\left({\sum}_{j}{y}_{{ij}}+\alpha \right)}}_{(\Theta)}\mathop{\prod}\limits_{j}f\left(\upsilon_{ij}\right)d{{\boldsymbol{\upsilon}}}_{i},$$

where ${\boldsymbol{\Gamma }}\left(\bullet \right)$ is the gamma function, ${{\boldsymbol{\upsilon }}}_{i}{\boldsymbol{=}}{{\boldsymbol{(}}{\upsilon }_{i1}{\boldsymbol{,}}{\boldsymbol{\ldots }}{\boldsymbol{,}}{\upsilon }_{i{n}_{i}}{\boldsymbol{)}}}^{T}$, and $f\left({\upsilon }_{{ij}}\right)=\frac{{\phi }^{\phi }}{{\boldsymbol{\Gamma }}\left(\phi \right)}{{\upsilon }_{{ij}}}^{\phi -1}{{\exp }}\left(-\phi {\upsilon }_{{ij}}\right)$ is the gamma density function defined in the NBGMM. It is not trivial to solve the high-dimensional integral in Eq. (6) because of the entanglement of ${\upsilon }_{{ij}}$ in$\Theta$, but when ${n}_{i}\to \infty$, we expect to obtain a simple approximation of the summation in $\Theta$ asymptotically by the law of large numbers (LLN). With this notion in mind, we rewrite $\Theta$ in Eq. (7) as

7 $$\Theta ={{\exp }}\left(-\left({\sum }_{j}{y}_{{ij}}+\alpha \right){{\log }}\left(\left(\lambda +{\sum }_{j}{\mu }_{{ij}}^{\ast }\right)\left(1+\frac{{\sum }_{j}\left(\left({\upsilon }_{{ij}}-1\right){\mu }_{{ij}}^{\ast }\right)}{\lambda +{\sum }_{j}{\mu }_{{ij}}^{\ast }}\right)\right)\right),$$

where ${\mu }_{{ij}}^{\ast }={{\exp }}\left({{{\boldsymbol{x}}}_{{ij}}}^{T}{\boldsymbol{\beta }}{\boldsymbol{+}}{{\log }}\left({\pi }_{{ij}}\right)\right)$. Now consider a Monte Carlo method to approximate the integral in Eq. (6) by sampling a large number of ${{\boldsymbol{\upsilon }}}_{i}$ from $f\left({{\boldsymbol{\upsilon }}}_{i}\right)$ and summing up the integrand evaluated at these ${{\boldsymbol{\upsilon }}}_{i}$. We show in Supplementary materials A.2 that under very mild conditions, given any $0 \,<\, \varepsilon \,\ll\, 1$, we have $\left|\frac{{\sum }_{j}\left(\left({\upsilon }_{{ij}}-1\right){\mu }_{{ij}}^{\ast }\right)}{\lambda +{\sum }_{j}{\mu }_{{ij}}^{\ast }}\right| \,<\, \varepsilon$ for almost all Monte Carlo samples when ${n}_{i}\to \infty$, that is, $\frac{{\sum }_{j}\left(\left({\upsilon }_{{ij}}-1\right){\mu }_{{ij}}^{\ast }\right)}{\lambda +{\sum }_{j}{\mu }_{{ij}}^{\ast }}\to 0$ in probability. Given this observation, applying a first-order Taylor expansion to the logarithm in Eq. (7), we can approximate the integral in Eq. (6) (See Supplementary Note A.3 for the derivation) by

8 $${\left(\lambda +{\sum }_{j}{\mu }_{{ij}}^{\ast }\right)}^{-\left({\sum }_{j}{y}_{{ij}}+\alpha \right)}\mathop{\prod }\limits_{j=1}^{{n}_{i}}{\int }_{0}^{1}{{\upsilon }_{{ij}}}^{{y}_{{ij}}}{{\exp }}\left(-{\check{\omega }}_{i}\left({\upsilon }_{{ij}}-1\right){\mu }_{{ij}}^{\ast }\right)\frac{{\phi }^{\phi }}{{\boldsymbol{\Gamma }}\left(\phi \right)}{{\upsilon }_{{ij}}}^{\phi -1}{{\exp }}\left(-\phi {\upsilon }_{{ij}}\right)d{\upsilon }_{{ij}},$$

where ${\check{\omega }}_{i}=\frac{{\sum }_{j}{y}_{{ij}}+\alpha }{\lambda +{\sum }_{j}{\mu }_{{ij}}^{\ast }}$. The integrand in Eq. (8) is now recognized as the kernel of a gamma distribution with respect to ${\upsilon }_{{ij}}$. Calculating this integral explicitly and substituting it into Eq. (6) give an approximated marginal log-likelihood

$${l}_{i}\left({\boldsymbol{\beta }}{\boldsymbol{,}}{\sigma }^{2},\phi \right)\approx {\widetilde{l}}_{i}\left({\boldsymbol{\beta }}{\boldsymbol{,}}{\sigma }^{2},\phi \right)$$

9 $$	=\alpha {{\log }}\lambda +{{\log }}\frac{{\boldsymbol{\Gamma }}\left({\sum }_{j}{y}_{{ij}}+\alpha \right)}{{\boldsymbol{\Gamma }}\left(\alpha \right)}+{\sum }_{j=1}^{{n}_{i}}{y}_{{ij}}{{\log }}\left({\mu }_{{ij}}^{\ast }\right)-\left({\sum }_{j}{y}_{{ij}}+\alpha \right){{\log }}\left(\lambda +{\sum }_{j}{\mu }_{{ij}}^{\ast }\right)\\ 	\quad+{\sum }_{j}\left(\phi {{\log }}\phi +{{\log }}\frac{{\boldsymbol{\Gamma }}\left({y}_{{ij}}+\phi \right)}{{\boldsymbol{\Gamma }}\left(\phi \right)}-\left({y}_{{ij}}+\phi \right){{\log }}\left(\phi +{\check{\omega }}_{i}{\mu }_{{ij}}^{\ast }\right)+{\check{\omega }}_{i}{\mu }_{{ij}}^{\ast }\right).$$

Thus, we may estimate $\left({\boldsymbol{\beta }}{\boldsymbol{,}}{\sigma }^{2},\phi \right)$ by optimizing ${\widetilde{l}}_{i}\left({\boldsymbol{\beta }}{\boldsymbol{,}}{\sigma }^{2},\phi \right)$, leading to an M-estimator^59–61^. Compared to the algorithms based on the LA, the optimization procedure of ${\widetilde{l}}_{i}\left({\boldsymbol{\beta }}{\boldsymbol{,}}{\sigma }^{2},\phi \right)$, described in a later section, becomes straightforward because the analytical form is available. As zero counts are prevalent in single-cell data, the evaluation of the log-gamma function ${{\log }}\frac{{\boldsymbol{\Gamma }}\left({y}_{{ij}}+\phi \right)}{{\boldsymbol{\Gamma }}\left(\phi \right)}$ in ${\widetilde{l}}_{i}\left({\boldsymbol{\beta }}{\boldsymbol{,}}{\sigma }^{2},\phi \right)$ can be saved for most cells. We obtain estimating equations by taking the first derivative of ${\widetilde{l}}_{i}\left({\boldsymbol{\beta }}{\boldsymbol{,}}{\sigma }^{2},\phi \right)$ (see Supplementary Note A.4), through which we show in Supplementary Note A.5 that ${\widetilde{l}}_{i}\left({\boldsymbol{\beta }}{\boldsymbol{,}}{\sigma }^{2},\phi \right)$ provides an asymptotically consistent estimate of $\left({\boldsymbol{\beta }}{\boldsymbol{,}}{\sigma }^{2},\phi \right)$ when ${n}_{i}\to \infty$ and $m\to \infty$. The consistency is also supported by the results in our simulation study described in the Results section.

As ${n}_{i}\to \infty$ is never achieved in real data, we then investigate the performance of using ${\widetilde{l}}_{i}\left({\boldsymbol{\beta }}{\boldsymbol{,}}{\sigma }^{2},\phi \right)$ in single-cell data with finite ${n}_{i}$. The major question is under which conditions the method works well and how large ${n}_{i}$ is required to produce an accurate estimate of ${\sigma }^{2}$ and $\phi$. We see from Eq. (7) that the accuracy of ${\widetilde{l}}_{i}\left({\boldsymbol{\beta }}{\boldsymbol{,}}{\sigma }^{2},\phi \right)$ depends on how close $\frac{{\sum }_{j}\left(\left({\upsilon }_{{ij}}-1\right){\mu }_{{ij}}^{\ast }\right)}{\lambda +{\sum }_{j}{\mu }_{{ij}}^{\ast }}$ is to zero, and it becomes an exact maximum likelihood estimate (MLE) if this term equals zero. By the Lindeberg central limit theorem, under mild conditions, $\frac{{\sum }_{j}\left(\left({\upsilon }_{{ij}}-1\right){\mu }_{{ij}}^{\ast }\right)}{\lambda +{\sum }_{j}{\mu }_{{ij}}^{\ast }}$ follows a zero-mean normal distribution with a variance at the rate of

$$\left(1+{c}^{2}\right)\Bigg/\left({n}_{i}\phi +\frac{2\phi \lambda }{E\left({\mu }_{{ij}}^{\ast }\right)}{\mathscr{+}}{\mathscr{O}}\left(\frac{1}{{n}_{i}}\right)\right),$$

where $c$ is the CV of the scaling factor ${\pi }_{{ij}}$ under the null hypothesis that cell-level variables have no fixed effects. Under an alternative hypothesis, the CV should also include the contribution from cell-level variables (see Supplementary Note A.6 for the detailed derivation). Intuitively, the estimation accuracy of NEBULA-LN should depend on the dominant term $(1+{c}^{2})/{n}_{i}\phi$ when ${n}_{i}$ is large, and this relationship was supported by the simulation study presented in the Results section by comparing the estimates between NEBULA-LN and NEBULA-HL, which uses an h-likelihood method (described in the next section). Interestingly, we find that a good estimate of $\phi$ in terms of MSE can be achieved even if ${\bar{n}}_{i}\phi$ is as small as 20, where ${\bar{n}}_{i}$ is the CPS value. However, the estimate of ${\sigma }^{2}$ requires a larger ${\bar{n}}_{i}\phi$, and $\kappa =\frac{{\bar{n}}_{i}\phi }{1+{c}^{2}}$ determines the resolution of estimating small ${\sigma }^{2}$. The simulation study in the Results section shows that $\kappa \, > \,200 \,\, {\rm{and}} \,\,800$ can empirically provide a good estimate of ${\sigma }^{2}$ as small as ~0.02 and ~0.005, respectively. As shown in the analysis of the droplet-based scRNA-seq data^5^, only ~1% of genes, all of which are very low-expression genes, have very large cell-level overdispersion (${\phi }^{-1} > 10$) in the exitatory neurons. Consequently, NEBULA-LN alone is sufficient for most genes when CPS > 200. The standard error (SE) of $\left({\boldsymbol{\beta }}{\boldsymbol{,}}{\sigma }^{2},\phi \right)$ can be obtained by the sandwich covariance estimator^62^. We find that the SE based on the Hessian matrix of ${\widetilde{l}}_{i}\left({\boldsymbol{\beta }}{\boldsymbol{,}}{\sigma }^{2},\phi \right)$ alone is practically accurate when ${\bar{n}}_{i}\phi$ is large. Instead, for better accuracy and efficiency, we choose to use the h-likelihood for computing its SE.

### Estimation using h-likelihood in NEBULA-HL

We use NEBULA-LN as a first-line solution to estimate ${\sigma }^{2}$ and $\phi$. If ${\bar{n}}_{i}\hat{\phi }$ or $\kappa$ is smaller than a predefined threshold, we then resort to NEBULA-HL to obtain a more accurate estimate. NEBULA-HL is based on an h-likelihood method^63^ requiring a two-layer iterative procedure. To derive the h-likelihood, we switch the integral order in ${L}_{i}\left({\boldsymbol{\beta }}{\boldsymbol{,}}{\sigma }^{2},\phi \right)$ and first integrate out ${\upsilon }_{{ij}}$, which leads to

10 $${L}_{i}\left({\boldsymbol{\beta }}{\boldsymbol{,}}{\sigma }^{2},\phi \right)\propto {\int }_{0}^{+{{\infty }}}{\prod }_{{\boldsymbol{j}}}\Bigg({\boldsymbol{\Gamma }}\left({y}_{{ij}}+\phi \right){{\exp }}\left({y}_{{ij}}\left({{{\boldsymbol{x}}}_{{ij}}}^{T}{\boldsymbol{\beta }}{\boldsymbol{+}}{{\log }}\left({\pi }_{{ij}}\right){\boldsymbol{+}}{{\log }}\left({\omega }_{i}\right)\right)\right) \\ {\left(\phi +{{\exp }}\left({{{\boldsymbol{x}}}_{{ij}}}^{T}{\boldsymbol{\beta }}{\boldsymbol{+}}{{\log }}\left({\pi }_{{ij}}\right){\boldsymbol{+}}{{\log }}\left({\omega }_{i}\right)\right)\right)}^{-\left({y}_{{ij}}+\phi \right)}\Bigg)f\left({\omega }_{i}\right)d{\omega }_{i}.$$

The h-likelihood method first optimizes the integrand in Eq. (10) with respect to $\left({\boldsymbol{\beta }}{\boldsymbol{,}}{\omega }_{i}\right)$ in the inner loop. Note that ${\omega }_{i}$ cannot be optimized with ${\boldsymbol{\beta }}$ directly because they are not in the canonical scale^63^. We thus convert the integrand in Eq. (10) to an h-likelihood by re-parametrizing the integral using ${\eta }_{i}={{\log }}\left({\omega }_{i}\right)$, which leads to the h-likelihood for individual $i$

11 $${{hl}}_{i}\left({\boldsymbol{\beta }}{\boldsymbol{,}}{\eta }_{i}{\rm{|}}{\sigma }^{2},\phi \right	)= \, {\sum }_{j}{y}_{{ij}}\left({{{\boldsymbol{x}}}_{{ij}}}^{T}{\boldsymbol{\beta }}{\boldsymbol{+}}{{\log }}\left({\pi }_{{ij}}\right){\boldsymbol{+}}{\eta }_{i}\right)\\ 	\quad-\left({y}_{{ij}}+\phi \right){{\log }}\left(\phi +{{\exp }}\left({{{\boldsymbol{x}}}_{{ij}}}^{T}{\boldsymbol{\beta }}{\boldsymbol{+}}{\log }\left({\pi }_{{ij}}\right){\boldsymbol{+}}{\eta }_{i}\right)\right)+\alpha {\eta }_{i}-\lambda {{\exp }}({\eta }_{i}).$$

We optimize ${\sum }_{i}{{hl}}_{i}\left({\boldsymbol{\beta }}{\boldsymbol{,}}{\eta }_{i}|{\sigma }^{2},\phi \right)$ by calculating its first and second derivatives of Eq. (11) (see Supplementary materials A.7 for more detail). As the log-h-likelihood is concave (both the negative binomial term and the penalizing term are concave), an NR algorithm practically converges fast and properly. The submatrix corresponding to ${\eta }_{i}$ in the Hessian matrix is diagonal, so the computational time for solving the inverse Hessian matrix is ${\mathscr{O}}$(*m*), which is ignorable. We also use this step to compute the SE of $\hat{{\boldsymbol{\beta }}}$ in NEBULA-LN by plugging in $({\hat{\sigma }}^{2},\hat{\phi })$ estimated from ${\widetilde{l}}_{i}\left({\boldsymbol{\beta }}{\boldsymbol{,}}{\sigma }^{2},\phi \right)$.

Given the current estimate $\left({{\boldsymbol{\beta }}}^{{\boldsymbol{\ast }}}{\boldsymbol{,}}{{\eta }_{i}}^{{\boldsymbol{\ast }}}\right)$, we then optimize $({\sigma }^{2},\phi )$ using the following marginal log-likelihood

12 $$\mathop{\sum}\limits_{i} l_{i}\left(\sigma^{2}, \phi | {\boldsymbol{\beta}}^{\ast },{\eta_{i}}^{\ast}\right)= \mathop{\sum}\limits_{i} hl_{i}\left({\boldsymbol{\beta}}^{\ast} ,{\eta_{i}}^{\ast} | \sigma^{2}, \phi \right) - \frac{1}{2}{\mathrm{log}}\left(\left| \frac{\partial ^{2} hl_{i}\left({\boldsymbol{\beta }} ,\eta_{i} | \sigma^{2},\phi \right)}{\partial {\eta_{i}}^{\ast 2}}\right|\right),$$

where $|\frac{\partial^{2}hl_{i}\left({\boldsymbol{\beta }}, \eta_{i} | \sigma^{2},\phi \right)}{\partial {\eta_{i}}^{\ast 2}}|$ is the logarithm of the absolute value of the determinant of the second derivative with respect to ${\eta }_{i}$ evaluated at ${\eta_{i}}^{\ast}$. The second term in Eq. (12) comes from the first-order LA and replacing $\frac{\partial^{2} hl_{i}\left({\boldsymbol{\beta }},\eta_{i}|\sigma^{2},\phi \right)}{\partial {\eta_{i}}^{2}}$ with $\frac{\partial^{2} hl_{i}\left({\boldsymbol{\beta }}, \eta_{i} | \sigma^{2},\phi \right)}{\partial (\eta_{i},{\boldsymbol{\beta }})^{2}}$ gives a restricted maximum likelihood (REML) estimate of $({\sigma }^{2},\phi )$. Similar to *lme4*^18^ and *coxmeg*^64^, we use the derivative-free algorithm bobyqa^65^ implemented in the *nloptr* R package^66^ for the optimization of ${\sum }_{i}{l}_{i}\left({\sigma }^{2},\phi |{{\boldsymbol{\beta }}}^{{\boldsymbol{\ast }}}{\boldsymbol{,}}{{\eta }_{i}}^{{\boldsymbol{\ast }}}\right)$. The number of iterations in this step grows rapidly with the dimension of the parameters. To reduce the iterations, we plug in $\hat{\phi }$ in ${\sum }_{i}{l}_{i}\left({\sigma }^{2},\phi |{{\boldsymbol{\beta }}}^{{\boldsymbol{\ast }}}{\boldsymbol{,}}{{\eta }_{i}}^{{\boldsymbol{\ast }}}\right)$ as we find that $\hat{\phi }$ from NEBULA-LN is accurate under mild conditions (e.g., ${\bar{n}}_{i}\phi > 20$). Thus, for most genes estimated by NEBULA-HL, we only perform the optimization for ${\sigma }^{2}$, which substantially reduces the number of iterations. In most cases, the algorithm can reach convergence within ~10 iterations.

The first-order LA proposed in Eq. (12) generally produces accurate estimates (as shown in Fig. 2). It, however, underestimates the subject-level overdispersion unignorably when the average count per subject is ≤2 (Supplementary Fig. S17). We find that this problem arises because the h-likelihood of the NBGMM becomes highly skewed if the counts among the cells are very sparse. This issue is unique to the NBGMM because the penalizing term $\lambda {{\exp}}({\eta }_{i})$ in the h-likelihood of the NBGMM is exponential while it is quadratic in the NBLMM (Supplementary Fig. S18). We, therefore, developed an efficient higher-order LA method^67,68^ to correct for this skewness for analyzing low-expression genes (See Supplementary Note A.9). This correction greatly improved the accuracy of the estimated subject-level overdispersion for low-expression genes (Supplementary Fig. S17).

### Computational implementation of NEBULA-LN

We assessed three algorithms for optimizing ${\widetilde{l}}_{i}\left({\boldsymbol{\beta }}{\boldsymbol{,}}{\sigma }^{2},\phi \right)$ in Eq. (8), including (i) the L-BFGS algorithm^69^ with box constraints implemented in *nloptr*^66^, (ii) the NR algorithm implemented in the *nlm* R function^70^, and (iii) a trust-region algorithm^71^ implemented in the *trust* R package (https://cran.r-project.org/web/packages/trust/index.html). The NR and the trust region algorithms require the Hessian matrix of ${\widetilde{l}}_{i}\left({\boldsymbol{\beta }}{\boldsymbol{,}}{\sigma }^{2},\phi \right)$. Inspired by^72^, we derive an approximate Hessian matrix (see Supplementary Note A.8) by taking the expectation for some elements to simplify the computation. As ${\sigma }^{2},\phi$ are restricted to be positive values, we reparameterize them using logarithm for the unconstrained optimization. In contrast, the L-BFGS algorithm requires only the estimating equations. We find that the NR algorithm using *nlm* is generally the fastest, and often converges within 10 iterations. However, the NR algorithm may not converge well for low-expression genes with a small CPC value, and occasionally the convergence depends on the initial values. In contrast, the L-BFGS and trust region algorithms are often slower, but much more robust, and both algorithms produce highly consistent estimates. The trust region algorithm takes fewer iterations, but can be slower than the L-BFGS algorithm when the dimension of ${\boldsymbol{\beta }}$ becomes larger. This is because the computation of the Hessian matrix is more computationally expensive with an increasing number of the parameters. As most genes in single-cell data have low-expression or zero counts, the evaluation of most log-gamma, and digamma functions in the marginal likelihood and the estimating equations can be saved or replaced using a recursive formula.

### Processing of the scRNA-seq data in PBMC

The scRNA-seq UMI raw count data in PBMC from the peripheral blood of 11 subjects (six healthy subjects and five patients with multiple sclerosis (MS) from a multiethnic population ranging from age 20 to 40) were obtained from the authors of ref. ^27^. The original count matrix contained 33,538 genes and 72,600 cells. In the QC step, we excluded outlier cells that satisfied any of the following criteria: total counts >5 median absolute deviations (MADs) away from the median, total features <100, total features >5 MADs, the percentage of counts from mitochondrial genes >5 MADs, and the percentage of counts from ribosomal genes <10%. We included 69,516 cells that passed the QC step in the downstream analysis. Clustering was performed using a routine provided by the Seurat package^73^. Specifically, the raw count matrix was normalized, highly variable genes were identified, and the normalized data were then scaled. Principal component (PC) analysis was performed on the scaled data and the top 20 PCs were selected based on an elbow plot of the eigenvalues for clustering and visualizing the cells using UMAP^74^.

### Analysis of marker genes and cell-type annotation

For each of the clusters in the PBMC scRNA-seq data, we built a binary variable of whether the cell belongs to this cluster. We performed a differential expression analysis of this binary variable using NEBULA, in which we included the number of features, the percentage of mitochondrial and ribosomal genes as covariates, and subjects as random effects. In each cluster, we defined marker genes as those with CPC > 1, the logarithm of fold change (log(FC)) > 0.2, and *p* < 1e-50 in the differential expression analysis. These criteria ensured that these marker genes had abundant expression and showed significantly higher expression in that cluster than in the remaining clusters. We then ranked them according to their log(FC) and used the top 40 genes (Supplementary Data 6) as an input to scCATCH^29^ to annotate these clusters (Some clusters had <40 marker genes based on the above criteria). In the first-round annotation, we used “Blood” as the tissue-specific cell taxonomy reference in scCATCH. The results showed that this reference database did not have refined cell types within T cells (e.g., naive or memory). Therefore we performed a second-round annotation using “Peripheral blood” as the reference for those clusters annotated as T cells in the previous round to further classify naive and memory T cells.

### Analysis of cell-sorting bulk RNA-seq data

The cell-sorting bulk RNA-seq data for naive CD4^+^ and CD8^+^ T cells were downloaded from the DICE (https://dice-database.org/downloads). The expression data sets for the two cell types included 103 and 104 healthy subjects, respectively, and were given as transcripts per million (TPM). We added a constant of 0.01 and used the log transformation of TPM as the response variable. As no information was available to match the subjects between the two cell types for each gene, we performed a differential expression analysis using simple linear regression with the cell type as the explanatory variable.

### Processing of the snRNA-seq data in the frontal cortex

The 48-subject snRNA-seq UMI raw count data from the ROSMAP, including 17,926 genes and 70,634 cells in the human frontal cortex were downloaded from Synapse (https://www.synapse.org/#!Synapse:syn21261143). All cells and genes included in the data passed the QC steps performed in the original study, more details of which are described in ref. ^5^. To better compare the marker genes identified by NEBULA with those from the original study, we adopted the clustering and cell-type annotation results provided by^5^, which included 34,976 excitatory neurons, 9196 inhibitory neurons, 18,235 oligodendrocytes, 3392 astrocytes, 1920 microglia, and 2627 OPCs. We did not include pericytes or endothelial cells due to their small sample sizes. To be more stringent, we performed a further QC step using scater^75^ to remove outlier cells whose total counts or total features were >3 MADs away from the median, which resulted in 69,458 cells. We also removed genes with CPC < 0.1% from the analysis of each cell type (CPC for a gene was calculated as its total UMI counts divided by the number of cells in the cell type). The subcell-type clustering annotation provided in ref. ^5^ was used in the analysis of marker genes for subclusters. There were 11, 12, 5, 4, 4, and 3 subclusters in the excitatory neurons, inhibitory neurons, oligodendrocytes, astrocytes, microglia, and OPCs, respectively. The percentage of ribosomal genes (RPS and RPL genes) was computed using the *PercentageFeatureSet* function in the *Seurat* R package^73^. Subject-level covariates, including age, sex, AD status, race, and *APOE* genotype in the ROSMAP project^76,77^, were downloaded from Synapse (https://www.synapse.org/#!Synapse:syn3157322).

The 32-subject snRNA-seq UMI raw count data from the ROSMAP and its cell type annotation were downloaded from Synapse (https://www.synapse.org/#!Synapse:syn21670836). The original count matrix comprised 33,694 genes and 114,972 cells in the human frontal cortex. We selected the 66,311 cells (14,675 excitatory neurons, 4256 inhibitory neurons, 29,478 oligodendrocytes, 9019 astrocytes, 3986 microglia, 841 endothelial cells, and 3243 OPCs) that passed the original QC step and had cell-type annotation as described in ref. ^78^. We further excluded 6259 very low-expression genes that showed positive counts in <3 cells.

### Co-expression analysis of the scRNA-seq and snRNA-seq data

The cell-level co-expression analysis of *BCL3* using 10,476 cells in the memory CD4+ T-cell population (Cluster 1) was performed for 14,770 genes (CPC > 0.05%) using four methods, including NEBULA without adjustment for confounders, NEBULA adjusting for total features, percentage of mitochondrial genes, and percentage of ribosomal genes, the Pearson correlation, and the Spearman correlation. In the two analyses using NEBULA, we first normalized the raw UMI count of *BCL3* by the library size of each cell. We then computed the mean of the normalized expression for each subject and subtracted it from the normalized expression. We included this centered expression of *BCL3* as the explanatory variable, the raw count of the other gene as the response variable, and 11 subjects as random effects. For the Pearson correlation and the Spearman correlation, we generated the centered expression of both genes and computed the correlation coefficient and its *p*-value using the *cor.test* R function.

In the co-expression analysis of *APOE* using NEBULA, we used the library size as the normalizing factor and included total features and percentage of ribosomal protein genes as the covariates. This is because genes in those cells with larger sequencing depth, more captured genes, or lower ribosomal protein gene expression had a higher co-occurrence rate. We did not include the percentage of mitochondrial genes because their values were very low in the snRNA-seq data and were not associated with the expression of most genes. The subjects were treated as random effects. To build the explanatory variable for the *APOE* expression, we first normalized *APOE* expression by dividing the raw count of *APOE* by its library size of each cell. We then subtracted from it the mean value of the normalized *APOE* expression across all cells of each subject so that the centered normalized *APOE* expression did not correlate with subject-level covariates. We only included genes with CPC ≥ 0.1% within each of the cell types. In the isoform-specific analysis, we separated all cells into three categories (e2e3, e3e3, or e4^+^) based on the *APOE* genotypes. We then applied the same normalizing and centering procedure as in the co-expression analysis of *APOE*. The meta-analysis of the summary statistics from the two snRNA-seq data sets was performed using the following fixed-effects model, $\beta ={\sum }_{i}{\beta }_{i}{w}_{i}/{\sum }_{i}{w}_{i}$ and ${\rm{sd}}\left(\beta \right)=1/\sqrt{{\sum }_{i}{w}_{i}}$, where $\beta$ and ${\rm{sd}}\left(\beta \right)$ are the log(FC) and its standard error of the combined effect, and ${w}_{i}=1/{\rm{var}}\left({\beta }_{i}\right)$ is the weight for study $i={\mathrm{1,2}}$.

The KEGG pathway enrichment analysis of the top genes correlated with *APOE* was performed using pathfindR^79^ with its default setting. In microglia, we used the genes having an FDR *p* < 0.05 as an input to pathfindR. In astrocytes, we found that no enriched pathway was identified when using all genes with FDR *p* < 0.05. This is probably because too many significant genes (>700) were included based on this cutoff, which failed to prioritize the top signals. Hence, we instead used the top 200 most significant genes as the input.

### Simulation study

We generated the number of CPS ${n}_{i}$ using a Poisson distribution for the balanced design and a negative binomial distribution with size = 3 for the unbalanced design. We evaluated scenarios with the number of subjects $m={\mathrm{30,50,100}}$ and the CPS value ${\bar{n}}_{i}={\mathrm{50,100,200,400,800}}$. The count data were generated based on the following generative model

$${y}_{{ij}} \sim {{\mathrm{Poisson}}}\left({\pi }_{{ij}}{{\exp }}\left({\beta }_{0}+{X}_{1}{\beta }_{1}+{X}_{2}{\beta }_{2}+{{\log }}\left({\upsilon }_{{ij}}\right)+{{\log }}({\omega }_{i})\right)\right),$$

$${\omega }_{i} \sim {{\mathrm{Gamma}}}\left(\frac{1}{{{\exp }}\left({\sigma }^{2}\right)-1},\frac{1}{{{\exp }}\left({\sigma }^{2}/2\right)({{\exp }}\left({\sigma }^{2}\right)-1)}\right),$$

$${\upsilon }_{{ij}} \sim {{\mathrm{Gamma}}}\left(\phi ,\phi \right),$$

where we considered ${\beta }_{0}$ ranging from −5 to 2, $\phi$ ranging from 0.01 to 100, and ${\sigma }^{2}$ ranging from 0.01 to 1. Under the scenarios of a constant scaling factor, we set ${\pi }_{{ij}}=1$, and otherwise we sampled ${\pi }_{{ij}}$ from ${{\mathrm{Gamma}}}({\mathrm{1,1}})$. We simulated a cell-level variable ${X}_{1}$ and a subject-level variable ${X}_{2}$ using a standard normal distribution. Under each of the settings, we generated 500 data sets to calculate the summary statistics. The MSE of an estimate (e.g., $\hat{\phi }$) was computed by $\frac{\sum {(\phi -\hat{\phi })}^{2}}{500}$ across the 500 data sets.

### Statistics and reproducibility

We compared the computational performance or summary statistics of NEBULA with four commonly used R packages (*lme4*^18^, *glmmTMB*^19^, *MASS*, and *INLA*^15^) for estimating the NBMMs and NBMs. We downloaded the *lme4* and *glmmTMB* R packages via https://cran.r-project.org/ and installed the *INLA* R package via http://www.r-inla.org/download. In *glmer.nb*, we assessed the default setting (nAGQ = 1), which is based on the LA^16^, and a faster but less accurate setting (nAGQ = 0), which is based on a penalized likelihood method^13^. The difference is that the LA method estimates the fixed effects in the marginal likelihood together with the variance components, while the latter estimates the fixed effects with the random effects in the penalized likelihood. In *glmmTMB*, we set family=nbinom2. In *INLA*, we set family  = “nbinomial”, and control.predictor = list(compute = FALSE). We set the other arguments as default. We adopted the L-BFGS optimization algorithm in NEBULA in all comparisons because we found that an NR algorithm^70^ was generally faster but less robust in terms of convergence. All benchmarks were run in R 3.5 on Windows 10 and Linux, separately. In the comparison with an NBM, we used the *glm.nb* function in the *MASS* R package.

### Reporting summary

Further information on research design is available in the Nature Research Reporting Summary linked to this article.

## Supplementary information

Supplementary Information

Description of Additional Supplementary Files

Supplementary Data 1

Supplementary Data 2

Supplementary Data 3

Supplementary Data 4

Supplementary Data 5

Supplementary Data 6

Reporting Summary

## Data Availability

This manuscript was prepared using limited access data sets obtained through Synapse (https://www.synapse.org/#!Synapse:syn3219045, https://www.synapse.org/#!Synapse:syn21670836, https://www.synapse.org/#!Synapse:syn18485175) and dbGaP (accession numbers: phs002222.v1.p1 (MS PBMC), phs001703.v3.p1 (DICE)).

## Code availability

The code of NEBULA (v1.1.7) can be downloaded and installed via https://github.com/lhe17/nebula, and archived in Zenodo^80^. The computational tools used for the data analysis include scCATCH v2.1 (https://github.com/ZJUFanLab/scCATCH), lme4 v1.1-26 (https://cran.r-project.org/web/packages/lme4/index.html), glmmTMB v1.0.2.1 (https://cran.r-project.org/web/packages/glmmTMB/index.html), MASS 7.3-53.1 (https://cran.r-project.org/web/packages/MASS/index.html), INLA v20.04.18 (https://www.r-inla.org/download-install).
