# Supplementary material for: NEBULA is a fast negative binomial mixed model for differential or co-expression analysis of large-scale multi-subject single-cell data
Source: Commun Biol. 2021 May 26;4:629. doi: 10.1038/s42003-021-02146-6 (PMC8155058; doi:10.1038/s42003-021-02146-6)
Supplement: Supplementary file 1 — Supplementary Information [file 42003_2021_2146_MOESM1_ESM.pdf]

Supplementary Note of "NEBULA is a fast negative binomial mixed model for differential or co-expression analysis of large-scale multi-subject single-cell data"

Table of Contents

A.1. Marginal expectation and variance of the NBGMM 1

A.2. Proof of convergence in probability 2

A.3. Approximation of the marginal likelihood using the WLLN 3

A.4. The estimating equations in NEBULA-LN 4

A.5. Asymptotic consistency of NEBULA-LN 5

A.6. Proof of asymptotic normality 9

A.7. The Newton-Raphson algorithm for optimizing the h-likelihood 11

A.8. The Hessian matrix for the Newton-Raphson algorithm in NEBULA-LN 12

A.9. The higher-order Laplace approximation in NEBULA-HL 13

References 17

A.1. Marginal expectation and variance of the NBGMM

We derive the marginal expectation and variance of y_{ij} under the NBGMM defined in eq. (5) in the main text. Here, by marginal expectation, we mean the expectation conditional only on $\boldsymbol{x}_{ij}$ and π_{ij}, but not ω_i or v_{ij}. Based on the model specification, the first and second moments of y_{ij} conditional on ω_i and v_{ij} are

$$E(y_{ij}|\omega_i, v_{ij}) = \pi_{ij}\exp(\boldsymbol{x}_{ij}\boldsymbol{\beta} + \log(\omega_i) + \log(v_{ij}))$$

$$E({y_{ij}}^2|\omega_i, v_{ij}) = E(y_{ij}|\omega_i, v_{ij}) + E^2(y_{ij}|\omega_i, v_{ij}),$$

where $\boldsymbol{x}_{ij} = (x_{ij1}, \dots, x_{ijk})$ and $\boldsymbol{\beta} = (\beta_1, \dots, \beta_k)^T$. As ω_i follows a gamma distribution defined by eq. (3), we have its first and second moments

$$E(\omega_i) = \frac{\alpha}{\lambda} = \exp(\sigma^2/2)$$

$$E({\omega_i}^2) = E^2(\omega_i) + Var(\omega_i) = \frac{\alpha^2 + \alpha}{\lambda^2} = \exp(2\sigma^2).$$

Thus, after taking the expectation over v_{ij} and ω_i and substituting $E(\omega_i)$, it follows that

$$E(y_{ij}) = E_{\omega_i}\left(E_{v_{ij}}\left(E(y_{ij}|\omega_i, v_{ij})\right)\right)$$

$$= E_{\omega_i}\left(E_{\upsilon_{ij}}(\upsilon_{ij})\pi_{ij}\exp\left(\boldsymbol{x}_{ij}\boldsymbol{\beta} + \log(\omega_i)\right)\right)$$

$$= E_{\omega_i}\left(\omega_i\pi_{ij}\exp\left(\boldsymbol{x}_{ij}\boldsymbol{\beta}\right)\right) = \pi_{ij}\exp\left(\boldsymbol{x}_{ij}\boldsymbol{\beta} + \frac{\sigma^2}{2}\right),$$

and

$$E\left(y_{ij}{}^2\right) = E_{\omega_i}\left(E_{\upsilon_{ij}}\left(E\left(y_{ij}{}^2|\omega_i, \upsilon_{ij}\right)\right)\right)$$

$$= E\left(y_{ij}\right) + E_{\omega_i}\left(E_{\upsilon_{ij}}\left(E^2\left(y_{ij}|\omega_i, \upsilon_{ij}\right)\right)\right)$$

$$= E\left(y_{ij}\right) + E_{\omega_i}\left(E_{\upsilon_{ij}}\left(\upsilon_{ij}{}^2\right)\pi_{ij}{}^2\exp\left(2\left(\boldsymbol{x}_{ij}\boldsymbol{\beta} + \log(\omega_i)\right)\right)\right)$$

$$= E\left(y_{ij}\right) + \left(1 + \frac{1}{\phi}\right)E_{\omega_i}\left(\omega_i{}^2\pi_{ij}{}^2\exp\left(2\boldsymbol{x}_{ij}\boldsymbol{\beta}\right)\right)$$

$$= E\left(y_{ij}\right) + \left(1 + \frac{1}{\phi}\right)\exp(2\sigma^2)\,\pi_{ij}{}^2\exp\left(2\boldsymbol{x}_{ij}\boldsymbol{\beta}\right)$$

$$= E\left(y_{ij}\right) + \left(1 + \frac{1}{\phi}\right)\exp(\sigma^2)\,E^2\left(y_{ij}\right)$$

Therefore, the marginal variance of y_{ij} is

$$Var\left(y_{ij}\right) = E\left(y_{ij}{}^2\right) - E^2\left(y_{ij}\right) = E\left(y_{ij}\right) + \left(\exp(\sigma^2) - 1\right)E^2\left(y_{ij}\right) + \frac{1}{\phi}\exp(\sigma^2)\,E^2\left(y_{ij}\right).$$

A.2. Proof of convergence in probability

Denote by $\underset{n_i\to\infty}{\text{plim}}$ convergence in probability of a sequence to a random variable when $n_i \to \infty$. Here, we show that under the condition that $\mu_{ij}^* = \exp\left(\boldsymbol{x}_{ij}\boldsymbol{\beta} + \log\left(\pi_{ij}\right)\right)$ has a finite non-zero first moment, we have

$$\underset{n_i\to\infty}{\text{plim}}\left(X_{n_i} = \frac{\sum_{j=1}^{n_i}\left(\upsilon_{ij} - 1\right)\mu_{ij}^*}{\lambda + \sum_j \mu_{ij}^*}\right) = 0,$$

that is, given any $\varepsilon > 0$, we have $\lim_{n_i\to\infty}\Pr\left(|X_{n_i}| > \varepsilon\right) = 0$. Rewrite the sequence X_{n_i} as

$$X_{n_i} = \frac{\frac{\sum_{j=1}^{n_i}\left(\upsilon_{ij}-1\right)\mu_{ij}^*}{n_i}}{\frac{\lambda+\sum_j \mu_{ij}^*}{n_i}}.$$

In fact, as υ_{ij} are *i.i.d.* gamma random variables defined by eq. (5), and μ_{ij}^* is assumed to be *i.i.d* with a finite first moment, by the weak law of large numbers (WLLN), it follows that

$$\underset{n_i\to\infty}{\text{plim}} \frac{\sum_{j=1}^{n_i}(v_{ij}-1)\mu_{ij}^*}{n_i} = E\left((v_{ij}-1)\mu_{ij}^*\right) = E(\mu_{ij}^*)E(v_{ij}) - E(\mu_{ij}^*) = 0,$$

in which we also use the assumption that the random effects v_{ij} and the fixed effects μ_{ij}^* are independent. Applying the WLLN again to the denominator gives

$$\underset{n_i\to\infty}{\text{plim}} \frac{\lambda+\sum_j \mu_{ij}^*}{n_i} = E(\mu_{ij}^*),$$

in which we use $\underset{n_i\to\infty}{\text{plim}} \frac{\lambda}{n_i} = 0$. Thus, it follows by Slutsky's theorem and the assumption $E(\mu_{ij}^*) > 0$ that

$$\underset{n_i\to\infty}{\text{plim}}(X_{n_i}) = 0.$$

A.3. Approximation of the marginal likelihood using the WLLN

Here we show that the integral for subject i in eq. (6)

$$I_i = \int_0^{+\infty} \left(\prod_j v_{ij}{}^{y_{ij}}\right) \underbrace{\left(\lambda + \sum_j \left(v_{ij} \exp\left(\boldsymbol{x}_{ij}\boldsymbol{\beta} + \log(\pi_{ij})\right)\right)\right)^{-(\sum_j y_{ij}+\alpha)}}_{(\Theta)} \prod_j f(v_{ij})\, d\boldsymbol{v}_i$$

can be approximated by eq. (8)

$$I_i \approx \left(\lambda + \sum_j \mu_{ij}^*\right)^{-(\sum_j y_{ij}+\alpha)} \times \prod_{j=1}^{n_i} \int_0^{+\infty} v_{ij}{}^{y_{ij}} \exp\left(-\frac{(\sum_j y_{ij}+\alpha)(v_{ij}-1)\mu_{ij}^*}{\lambda + \sum_j \mu_{ij}^*}\right) \frac{\phi^\phi}{\boldsymbol{\Gamma}(\phi)} v_{ij}{}^{\phi-1} \exp(-\phi v_{ij})\, dv_{ij}$$

under the condition $n_i \to \infty$. Rewrite Θ in eq.(6) as

$$\Theta = \exp\left(-(\textstyle\sum_j y_{ij} + \alpha) \log\left((\lambda + \sum_j \mu_{ij}^*)\left(1 + \frac{\sum_j((v_{ij}-1)\mu_{ij}^*)}{\lambda+\sum_j \mu_{ij}^*}\right)\right)\right).$$

As proved in A.2 that $\underset{n_i\to\infty}{\text{plim}}\left(\frac{\sum_j((v_{ij}-1)\mu_{ij}^*)}{\lambda+\sum_j \mu_{ij}^*}\right) = 0$, we ignore the contribution to the integral from those $\boldsymbol{v}_i \notin \mathcal{S}$ where $\mathcal{S} := \left(\boldsymbol{v}_i \middle| \left|\frac{\sum_j((v_{ij}-1)\mu_{ij}^*)}{\lambda+\sum_j \mu_{ij}^*}\right| < \varepsilon \ll 1\right)$ for a small positive ε because $P(\boldsymbol{v}_i \notin \mathcal{S})$ is very small when n_i is large. Thus, plugging $f(v_{ij})$ with the gamma density function defined in eq. (5), we can end up with eq. (8) as follows

$$\int_0^{+\infty} \left(\prod_j {v_{ij}}^{y_{ij}}\right) \exp\left(-\left(\sum_j y_{ij} + \alpha\right) \log\left(\left(\lambda + \sum_j \mu_{ij}^*\right)\left(1 + \frac{\sum_j\left((v_{ij}-1)\mu_{ij}^*\right)}{\lambda+\sum_j \mu_{ij}^*}\right)\right)\right) \times$$
$$\prod_{j=1}^{n_i} \frac{\phi^\phi}{\Gamma(\phi)} {v_{ij}}^{\phi-1} \exp(-\phi v_{ij})\, d\boldsymbol{v}_i$$

$$\approx \int_{\boldsymbol{v}_i \in \mathcal{S}} \left(\prod_j {v_{ij}}^{y_{ij}}\right) \exp\left(-\left(\sum_j y_{ij} + \alpha\right)\left(\log\left(\lambda + \sum_j \mu_{ij}^*\right) + \log\left(1 + \frac{\sum_j\left((v_{ij}-1)\mu_{ij}^*\right)}{\lambda+\sum_j \mu_{ij}^*}\right)\right)\right) \times$$
$$\prod_{j=1}^{n_i} \frac{\phi^\phi}{\Gamma(\phi)} {v_{ij}}^{\phi-1} \exp(-\phi v_{ij})\, d\boldsymbol{v}_i$$

$$\approx \int_{\boldsymbol{v}_i \in \mathcal{S}} \left(\prod_j {v_{ij}}^{y_{ij}}\right) \exp\left(-\left(\sum_j y_{ij} + \alpha\right)\left(\log\left(\lambda + \sum_j \mu_{ij}^*\right) + \frac{\sum_j\left((v_{ij}-1)\mu_{ij}^*\right)}{\lambda+\sum_j \mu_{ij}^*}\right)\right) \times$$
$$\prod_{j=1}^{n_i} \frac{\phi^\phi}{\Gamma(\phi)} {v_{ij}}^{\phi-1} \exp(-\phi v_{ij})\, d\boldsymbol{v}_i$$

$$\approx \exp\left(-\left(\sum_j y_{ij} + \alpha\right) \log\left(\lambda + \sum_j \mu_{ij}^*\right)\right) \int_0^{+\infty} \left(\prod_j {v_{ij}}^{y_{ij}}\right) \exp\left(-\left(\sum_j y_{ij} + \alpha\right) \frac{\sum_j\left((v_{ij}-1)\mu_{ij}^*\right)}{\lambda+\sum_j \mu_{ij}^*}\right) \times$$
$$\prod_{j=1}^{n_i} \frac{\phi^\phi}{\Gamma(\phi)} {v_{ij}}^{\phi-1} \exp(-\phi v_{ij})\, d\boldsymbol{v}_i$$

$$= \left(\lambda + \sum_j \mu_{ij}^*\right)^{-\left(\sum_j y_{ij}+\alpha\right)} \prod_{j=1}^{n_i} \int_0^{+\infty} {v_{ij}}^{y_{ij}} \exp\left(\frac{-\left(\sum_j y_{ij}+\alpha\right)}{\lambda+\sum_j \mu_{ij}^*}(v_{ij}-1)\mu_{ij}^*\right) \frac{\phi^\phi}{\Gamma(\phi)} {v_{ij}}^{\phi-1} \exp(-\phi v_{ij})\, dv_{ij}$$

where the approximations in the second and fourth lines are due to ignoring and adding those Monte Carlo samples $\boldsymbol{v}_i \notin \mathcal{S}$, respectively. The approximation in the third line uses the first-order Taylor expansion of a logarithm function, i.e., $\log(1+x) \approx x$ for x close to zero. In the last equation, we change the order between the integral and the product because v_{ij} are now completely factorized in the integrand. After moving the terms $\exp\left(\frac{\left(\sum_j y_{ij}+\alpha\right)\mu_{ij}^*}{\lambda+\sum_j \mu_{ij}^*}\right)$ out of the integral, the integrand is now recognized as a kernel of a gamma density function with respect to v_{ij}. Calculating this integral explicitly gives eq. (9).

A.4. The estimating equations in NEBULA-LN

The estimating equations in NEBULA-LN are obtained by taking the first derivative of the approximated marginal likelihood $\sum_i \tilde{l}_i(\boldsymbol{\beta}, \sigma^2, \phi)$ with respect to the parameters $(\boldsymbol{\beta}, \sigma^2, \phi)$, where

$$\tilde{l}_i(\boldsymbol{\beta}, \sigma^2, \phi) = \alpha\log\lambda + \log\frac{\Gamma\left(\sum_j y_{ij}+\alpha\right)}{\Gamma(\alpha)} + \sum_{j=1}^{n_i} y_{ij}\log\left(\mu_{ij}^*\right) - \left(\sum_j y_{ij} + \alpha\right)\log\left(\lambda + \sum_j \mu_{ij}^*\right) +$$
$$\sum_j \left(\phi\log\phi + \log\frac{\Gamma(y_{ij}+\phi)}{\Gamma(\phi)} - (y_{ij}+\phi)\log\left(\phi + \frac{\left(\sum_j y_{ij}+\alpha\right)\mu_{ij}^*}{\lambda+\sum_j \mu_{ij}^*}\right) + \frac{\left(\sum_j y_{ij}+\alpha\right)\mu_{ij}^*}{\lambda+\sum_j \mu_{ij}^*}\right).$$

To simplify the notations, we define

$$\check{\mu}_i := \lambda + \sum_j \mu_{ij}^*$$

$$\breve{\omega}_i := \frac{\sum_j y_{ij} + \alpha}{\breve{\mu}_i}$$

$$\breve{\upsilon}_{ij} := \frac{\phi + y_{ij}}{\phi + \breve{\omega}_i \mu_{ij}^*}.$$

We denote by α' and λ' the first derivatives of α and λ with respect to σ^2, respectively (i.e., $\alpha' = \frac{d\alpha}{d\sigma^2}$ and $\lambda' = \frac{d\lambda}{d\sigma^2}$). Through tedious but relatively straightforward calculation, the first derivatives are

$$D_{n_i,m}^{\boldsymbol{\beta}} = \sum_i \frac{\partial \tilde{l}_i(\boldsymbol{\beta}, \sigma^2, \phi)}{\partial \boldsymbol{\beta}} = \sum_i \boldsymbol{X}_i{}^T \boldsymbol{y}_i - \frac{\breve{\omega}_i}{\breve{\mu}_i}\left(\sum_j (1 - \breve{\upsilon}_{ij})\mu_{ij}^*\right) \boldsymbol{X}_i{}^T \boldsymbol{\mu}_i^* - \breve{\omega}_i \boldsymbol{X}_i{}^T (\boldsymbol{\mu}_i^* \odot \breve{\boldsymbol{\upsilon}}_i)$$

$$D_{n_i,m}^{\phi} = \sum_i \frac{\partial \tilde{l}_i(\boldsymbol{\beta}, \sigma^2, \phi)}{\partial \phi} = \sum_i \sum_j \left(\log\phi + \Psi(y_{ij} + \phi) - \Psi(\phi) - \log(\phi + \breve{\omega}_i \mu_{ij}^*) + 1 - \breve{\upsilon}_{ij}\right)$$

$$\begin{aligned} D_{n_i,m}^{\sigma^2} &= \sum_i \frac{\partial \tilde{l}_i(\boldsymbol{\beta}, \sigma^2, \phi)}{\partial \sigma^2} \\ &= \sum_i \alpha' \left(\log\lambda + \Psi\left(\sum_j y_{ij} + \alpha\right) - \Psi(\alpha) - \log(\breve{\mu}_i)\right) + \lambda' \left(\frac{\alpha}{\lambda} - \breve{\omega}_i\right) \\ &\quad + \frac{\alpha' - \lambda' \breve{\omega}_i}{\breve{\mu}_i} \sum_j (1 - \breve{\upsilon}_{ij}) \mu_{ij}^* \end{aligned}$$

where $\Psi(x) = \frac{d\log(\Gamma(x))}{dx}$ is the digamma function, $\odot$ stands for the Hadamard product, $\boldsymbol{y}_i = (y_{i1}, \dots, y_{in_i})^T$, $\boldsymbol{X}_i = (\boldsymbol{x}_{i1}{}^T, \dots, \boldsymbol{x}_{in_i}{}^T)^T$, $\boldsymbol{\mu}_i^* = (\mu_{i1}^*, \dots, \mu_{in_i}^*)^T$, and $\breve{\boldsymbol{\upsilon}}_i = (\breve{\upsilon}_{i1}, \dots, \breve{\upsilon}_{in_i})^T$. Setting $D_{n_i,m}^{\boldsymbol{\beta}}$, $D_{n_i,m}^{\phi}$ and $D_{n_i,m}^{\sigma^2}$ to zero gives a group of estimating equations for $(\boldsymbol{\beta}, \sigma^2, \phi)$.

A.5. Asymptotic consistency of NEBULA-LN

We prove that the estimating equations $D_{n_i,m}$ given in A.4 lead to a consistent estimator $T_{n_i,m}$ for $(\boldsymbol{\beta}, \sigma^2, \phi)$ when $n_i \to \infty$ and $m \to \infty$, that is, we need to show that $T_{n_i,m}$ asymptotically converges in probability to its true value

$$\underset{\substack{n_i \to \infty \\ m \to \infty}}{\text{plim}} T_{n_i,m} = (\boldsymbol{\beta}, \sigma^2, \phi)$$

The estimator $T_{n_i,m}$ defined implicitly by the sequence of solutions to the estimation equations $D_{n_i,m}^{\boldsymbol{\beta},\sigma^2,\phi} = 0$ is an M-estimator [1] (also known as a Z-estimator [2]). Asymptotically, the empirical equations $D_{n_i,m}^{\boldsymbol{\beta},\sigma^2,\phi}$ under suitable normalization (that is, divided by n_i and/or m as described below) will converge to its deterministic equation $D^{\boldsymbol{\beta},\sigma^2,\phi}$ by the WLLN (see e.g., [3] section 7.2).

We assume that the following regularity conditions are satisfied. First, the model is identifiable within the parameter space. This requires that, for example, the columns of the design matrix for $\boldsymbol{\beta}$ should be linearly independent. Additionally, we assume that the parameter space that we are interested in lies in

a compact subset and contains the true values that are a unique zero of $D^{\boldsymbol{\beta},\sigma^2,\phi}$. As $D^{\boldsymbol{\beta},\sigma^2,\phi}_{n_i,m}$ is continuous, it is also bounded in the compact subset. Provided that these conditions are met, the asymptotic consistency of $T_{n_i,m}$ amounts to proving that the true values of $(\boldsymbol{\beta},\sigma^2,\phi)$ are the solutions to $D^{\boldsymbol{\beta},\sigma^2,\phi}=0$ (see e.g., [3] section 7.2 or [2] section 5.2).

First, we notice that $D^{\boldsymbol{\beta}}_{n_i,m}$ and $D^{\phi}_{n_i,m}$ are $o_p(mn_i)$ and $D^{\sigma^2}_{n_i,m}$ is only $o_p(m)$. Therefore, $m\to\infty$ is necessary in order to have an asymptotically consistent $\hat{\sigma}^2$. This means that we need a large number of subjects to obtain an accurate subject-level overdispersion σ^2. On the other hand, $n_i\to\infty$ is required by all three equations to converge.

We begin by examining the term $1-\breve{v}_{ij}$ present in $D^{\phi}_{n_i,m}$. By the WLLN and $E\left(y_{ij}|\mu^*_{ij},\omega_i\right)=\omega_i\mu^*_{ij}$ in A.1, it follows that

$$\operatorname*{plim}_{n_i\to\infty}\breve{\omega}_i=\operatorname*{plim}_{n_i\to\infty}\frac{\frac{\sum_j y_{ij}+\alpha}{n_i}}{\frac{\lambda+\sum_j\mu^*_{ij}}{n_i}}=\frac{\omega_i E\left(\mu^*_{ij}\right)}{E\left(\mu^*_{ij}\right)}=\omega_i.$$

Then because $\breve{v}_{ij}$ is continuous at ω_i uniformly in y_{ij} and x_{ij}, and its first moment is finite, by the conditional expectation and Lemma 7.2.2A in [3], we have

$$\operatorname*{plim}_{n_i\to\infty}\frac{\sum_j\breve{v}_{ij}}{n_i}=E_{x_{ij},y_{ij}}\left(\frac{\phi+y_{ij}}{\phi+\omega_i\mu^*_{ij}}\right)=E_{x_{ij}}\left(\frac{\phi+E\left(y_{ij}|\mu^*_{ij},\omega_i\right)}{\phi+\omega_i\mu^*_{ij}}\right)=E_{x_{ij}}\left(\frac{\phi+\omega_i\mu^*_{ij}}{\phi+\omega_i\mu^*_{ij}}\right)=1.$$

Hence, the terms in $D^{\phi}_{n_i,m}$ involving $1-\breve{v}_{ij}$ cancel out. By this argument, we can also see that the terms involving $\frac{\sum_j(1-\breve{v}_{ij})\mu^*_{ij}}{\breve{\mu}_i}$ in $D^{\boldsymbol{\beta}}_{n_i,m}$ and $D^{\sigma^2}_{n_i,m}$ are all $o_p(1)$ under $n_i\to\infty$. This is because under the assumption given in A.2 that μ^*_{ij} has a finite non-zero first moment, we have

$$\operatorname*{plim}_{n_i\to\infty}\frac{\sum_j(1-\breve{v}_{ij})\mu^*_{ij}}{\breve{\mu}_i}=\operatorname*{plim}_{n_i\to\infty}\frac{\sum_j(1-\breve{v}_{ij})\mu^*_{ij}/n_i}{\breve{\mu}_i/n_i}=\frac{o_p(1)}{E(\mu^*_{ij})}=0$$

Following [4],

$$\operatorname*{plim}_{m\to\infty}\frac{\sum_i\breve{\omega}_i}{m}=E(\breve{\omega}_i)=E\left(E(\breve{\omega}_i|\omega_i)\right)$$

$$=E\left(\frac{\sum_j E\left(y_{ij}|\omega_i\right)+\alpha}{\lambda+\sum_j\mu^*_{ij}}\right)=E\left(\frac{\omega_i\sum_j\mu^*_{ij}+\alpha}{\lambda+\sum_j\mu^*_{ij}}\right)=\frac{\frac{\alpha}{\lambda}\sum_j\mu^*_{ij}+\alpha}{\lambda+\sum_j\mu^*_{ij}}=\frac{\alpha}{\lambda}.$$

Hence, the term $\frac{\alpha}{\lambda}-\breve{\omega}_i$ in $D^{\sigma^2}_{n_i,m}$ cancels out. In $D^{\boldsymbol{\beta}}_{n_i,m}$, it remains to show that under suitable normalization,

$$\operatorname*{plim}_{n_i\to\infty}\frac{\boldsymbol{X}_i{}^T\boldsymbol{y}_i-\breve{\omega}_i\boldsymbol{X}_i{}^T(\boldsymbol{\mu}^*_i\odot\breve{\boldsymbol{v}}_i)}{n_i}=0.$$

In fact, for the kth component in $\boldsymbol{\beta}$, by the WLLN and conditional expectation, we have for the first term

$$\underset{n_i\to\infty}{\text{plim}}\frac{\boldsymbol{X}_i{}^T\boldsymbol{y}_i}{n_i}=\underset{n_i\to\infty}{\text{plim}}\frac{\sum_j x_{ijk}y_{ij}}{n_i}=E_x\left(x_{ijk}E_y\big(y_{ij}|x_{ijk}\big)\right)=\omega_i E_x\big(x_{ijk}\mu_{ij}^*\big)$$

and for the second term by the continuous mapping theorem, Slutsky's theorem and Lemma 7.2.2A in [3],

$$\underset{n_i\to\infty}{\text{plim}}\frac{\boldsymbol{X}_i{}^T\breve{\omega}_i(\boldsymbol{\mu}_i^*\odot\breve{\boldsymbol{v}}_i)}{n_i}=\underset{n_i\to\infty}{\text{plim}}\breve{\omega}_i\cdot\underset{n_i\to\infty}{\text{plim}}\frac{\sum_j x_{ijk}\mu_{ij}^*\breve{v}_{ij}}{n_i}=\omega_i E_x\left(\frac{x_{ijk}\mu_{ij}^*\left(\phi+E_y\big(y_{ij}|\mu_{ij}^*,\omega_i\big)\right)}{\phi+\omega_i\mu_{ij}^*}\right)$$
$$=\omega_i E_x\big(x_{ijk}\mu_{ij}^*\big)$$

which proves that $\underset{\substack{n_i\to\infty\\ m\to\infty}}{\text{plim}}\frac{D_{n_i,m}^{\boldsymbol{\beta}}}{n_i}=0$.

In $D_{n_i,m}^{\sigma^2}$, it remains to show that $\underset{\substack{n_i\to\infty\\ m\to\infty}}{\text{plim}}\frac{1}{m}\big(\log\lambda-\Psi(\alpha)+\Psi\big(\sum_j y_{ij}+\alpha\big)-\log(\breve{\mu}_i)\big)=0$. As ω_i follows a gamma distribution defined by eq. (3), it can be verified based on the density function of $\log(\omega_i)$ (also see [4]) that its expectation is

$$E(\log(\omega_i))=\Psi(\alpha)-\log\lambda.$$

Now consider the posterior distribution of ω_i conditional on $\boldsymbol{y}_i$ and $\boldsymbol{v}_i$, which can be shown as

$$f(\omega_i|\boldsymbol{y}_i,\boldsymbol{v}_i)$$
$$\propto f(\boldsymbol{y}_i|\omega_i,\boldsymbol{v}_i)f(\omega_i)$$
$$=\prod_j Pois\big(y_{ij}|\omega_i v_{ij}\mu_{ij}^*\big)\,Gamma(\omega_i|\alpha,\lambda)$$
$$=Gamma\big(\alpha+\textstyle\sum_j y_{ij},\lambda+\sum_j v_{ij}\mu_{ij}^*\big).$$

Therefore, it follows that

$$E(\log(\omega_i)\,|\boldsymbol{y}_i,\boldsymbol{v}_i)=\Psi\left(\sum_j y_{ij}+\alpha\right)-\log\left(\lambda+\sum_j v_{ij}\mu_{ij}^*\right)$$

and

$$E(\log(\omega_i)\,|\boldsymbol{y}_i)=E_{\boldsymbol{v}_i}\big(E(\log(\omega_i)\,|\boldsymbol{y}_i,\boldsymbol{v}_i)\big)=\Psi\left(\sum_j y_{ij}+\alpha\right)-E_{\boldsymbol{v}_i}\log\left(\lambda+\sum_j v_{ij}\mu_{ij}^*\right)$$

Under the limit $m\to\infty$, by substituting $E(\log(\omega_i)\,|\boldsymbol{y}_i)$, we have

$$\underset{m\to\infty}{\text{plim}}\frac{\sum_i\Psi\big(\sum_j y_{ij}+\alpha\big)-\log\breve{\mu}_i}{m}$$

$$= \underset{m\to\infty}{\text{plim}} \frac{\sum_i \Psi\left(\sum_j y_{ij} + \alpha\right) - E_{\boldsymbol{v}_i} \log\left(\lambda + \sum_j v_{ij}\mu_{ij}^*\right) + E_{\boldsymbol{v}_i} \log\left(\lambda + \sum_j v_{ij}\mu_{ij}^*\right) - \log \breve{\mu}_i}{m}$$

$$= \underset{m\to\infty}{\text{plim}} \frac{\sum_i \Psi\left(\sum_j y_{ij} + \alpha\right) - E_{\boldsymbol{v}_i} \log\left(\lambda + \sum_j v_{ij}\mu_{ij}^*\right)}{m} + \lim_{m\to\infty} \frac{\sum_i E_{\boldsymbol{v}_i} \log\left(\lambda + \sum_j v_{ij}\mu_{ij}^*\right) - \log \breve{\mu}_i}{m}$$

$$= E_{\boldsymbol{y}_i}\left(E(\log(\omega_i) \mid \boldsymbol{y}_i)\right) + \lim_{m\to\infty} \frac{\sum_i E_{\boldsymbol{v}_i} \log\left(\frac{\lambda + \sum_j v_{ij}\mu_{ij}^*}{\breve{\mu}_i}\right)}{m} = E(\log(\omega_i)).$$

The second term in the above equation converges to zero under $n_i \to \infty$, that is,

$$\lim_{n_i\to\infty} E_{\boldsymbol{v}_i} \log\left(\frac{\lambda + \sum_j v_{ij}\mu_{ij}^*}{\breve{\mu}_i}\right) = 0.$$

This is because by WLLN, Slutsky's theorem, and the continuous mapping theorem, we have

$$\underset{n_i\to\infty}{\text{plim}} \log\left(\frac{\lambda + \sum_j v_{ij}\mu_{ij}^*}{\breve{\mu}_i}\right) = \underset{n_i\to\infty}{\text{plim}} \log\left(\frac{\lambda + \sum_j v_{ij}\mu_{ij}^*}{\lambda + \sum_j \mu_{ij}^*}\right) = \underset{n_i\to\infty}{\text{plim}} \log\left(\frac{\frac{\lambda + \sum_j v_{ij}\mu_{ij}^*}{n_i}}{\frac{\lambda + \sum_j \mu_{ij}^*}{n_i}}\right) = 0.$$

Under the reasonable and mild assumption that $\frac{\lambda+\sum_j v_{ij}\mu_{ij}^*}{\breve{\mu}_i}$ has a finite second moment, $\frac{\lambda+\sum_j v_{ij}\mu_{ij}^*}{\breve{\mu}_i}$ is uniformly integrable. By Theorem 1.4A in [3], convergence in probability implies convergence in mean in this case. Hence, we end up with $\underset{\substack{n_i\to\infty \\ m\to\infty}}{\text{plim}} \frac{D_{n_i,m}^{\sigma^2}}{m} = 0$.

Finally, we show that $\underset{\substack{n_i\to\infty \\ m\to\infty}}{\text{plim}} \frac{1}{m}\sum_{i=1}^{m} \frac{1}{n_i}\sum_{j=1}^{n_i} \log\phi + \Psi\left(y_{ij} + \phi\right) - \Psi(\phi) - \log\left(\phi + \breve{\omega}_i\mu_{ij}^*\right) = 0$ in $D_{n_i,m}^{\phi}$. As v_{ij} follows a gamma distribution defined by eq. (3), it can be verified that

$$E\left(\log(v_{ij})\right) = \Psi(\phi) - \log\phi.$$

Again, consider the conditional posterior distribution

$$f\left(v_{ij} \middle| y_{ij}, \omega_i\right)$$

$$\propto f\left(y_{ij} \middle| \omega_i, v_{ij}\right) f\left(v_{ij}\right)$$

$$= Pois\left(y_{ij} \mid \omega_i v_{ij}\mu_{ij}^*\right) Gamma\left(v_{ij} \mid \phi, \phi\right)$$

$$= Gamma\left(y_{ij} + \phi, \omega_i\mu_{ij}^* + \phi\right).$$

Therefore, conditional on ω_i, we have

$$E\left(\log(v_{ij}) \mid y_{ij}, \omega_i\right) = \Psi\left(y_{ij} + \phi\right) - \log\left(\phi + \omega_i\mu_{ij}^*\right),$$

and

$$E\left(\log\left(v_{ij}\right)|y_{ij}\right) = E_{\omega_i}\left(E\left(\log\left(v_{ij}\right)|y_{ij},\omega_i\right)\right) = \Psi\left(y_{ij}+\phi\right) - E_{\omega_i}\left(\log\left(\phi+\omega_i\mu_{ij}^*\right)\right)$$

Now by the continuous mapping theorem and plugging in the result $\operatorname*{plim}_{n_i\to\infty}\breve{\omega}_i = \omega_i$, it follows that

$$\operatorname*{plim}_{\substack{n_i\to\infty\\ m\to\infty}}\frac{1}{m}\sum_i\frac{\sum_j\Psi\left(y_{ij}+\phi\right)-\log\left(\phi+\breve{\omega}_i\mu_{ij}^*\right)}{n_i}$$

$$=\operatorname*{plim}_{\substack{n_i\to\infty\\ m\to\infty}}\frac{1}{m}\sum_i\frac{\sum_j\Psi\left(y_{ij}+\phi\right)-\log\left(\phi+\omega_i\mu_{ij}^*\right)}{n_i}.$$

Applying the WLLN to the first summation over ω_i and then to the second summation over y_{ij}, it follows that

$$\operatorname*{plim}_{\substack{n_i\to\infty\\ m\to\infty}}\frac{1}{m}\sum_i\frac{\sum_j\Psi\left(y_{ij}+\phi\right)-\log\left(\phi+\omega_i\mu_{ij}^*\right)}{n_i}$$

$$=\operatorname*{plim}_{n_i\to\infty}E_{\omega_i}\frac{\sum_j\Psi\left(y_{ij}+\phi\right)-\log\left(\phi+\omega_i\mu_{ij}^*\right)}{n_i}$$

$$=E_{\omega_i}\left(E_{y_{ij}}\left(\Psi\left(y_{ij}+\phi\right)-\log\left(\phi+\omega_i\mu_{ij}^*\right)\right)\right)$$

$$=E_{\omega_i}\left(E_{y_{ij}}\left(E\left(\log\left(v_{ij}\right)|y_{ij},\omega_i\right)\right)\right)=E\left(\log\left(v_{ij}\right)\right)$$

This completes the proof of the asymptotical consistency of the estimator $T_{n_i,m}$.

A.6. Proof of asymptotic normality

We show that under mild conditions and the null model, $X_{n_i} = \frac{\sum_j\left(\left(v_{ij}-1\right)\mu_{ij}^*\right)}{\lambda+\sum_j\mu_{ij}^*}$ asymptotically follows a zero-mean normal distribution with a variance at the rate of $1/\left(n_i\phi+\frac{2\phi\lambda}{E\left(\mu_{ij}^*\right)}+O\left(\frac{1}{n_i}\right)\right)$. In A.1., we have shown that

$$\operatorname*{plim}_{n_i\to\infty}\left(X_{n_i}=\frac{\sum_{j=1}^{n_i}\left(v_{ij}-1\right)\mu_{ij}^*}{\lambda+\sum_j\mu_{ij}^*}\right)=0.$$

Hence, it remains to prove the asymptotical normality of X_{n_i} and show that the variance of X_{n_i} is $1/\left(n_i\phi+\frac{2\phi\lambda}{E\left(\mu_{ij}^*\right)}+O\left(\frac{1}{n_i}\right)\right)$. As v_{ij} is *i.i.d* gamma random variable, by the Lindeberg central limit theorem, when $n_i\to\infty$ and the Lindeberg's condition is satisfied, we have

$$\frac{\sum_j\left((v_{ij}-1)\mu_{ij}^*\right)}{\sqrt{\sum_j\left(Var\left((v_{ij}-1)\mu_{ij}^*\right)\right)}}\xrightarrow{d}\mathcal{N}(0,1),$$

where $\xrightarrow{d}$ denotes convergence in distribution. Then, plugging in $Var(v_{ij})=1/\phi$, it follows that

$$X_{n_i}=\frac{\sum_j\left((v_{ij}-1)\mu_{ij}^*\right)}{\lambda+\sum_j\mu_{ij}^*}$$

$$=\frac{\sqrt{\sum_j\left(Var\left((v_{ij}-1)\mu_{ij}^*\right)\right)}}{\lambda+\sum_j\mu_{ij}^*}\frac{\sum_j\left((v_{ij}-1)\mu_{ij}^*\right)}{\sqrt{\sum_j\left(Var\left((v_{ij}-1)\mu_{ij}^*\right)\right)}}$$

$$\xrightarrow{d}N\left(0,\tau_{X_{n_i}}^2=\frac{\sum_j\left(Var\left((v_{ij}-1)\mu_{ij}^*\right)\right)}{\left(\lambda+\sum_j\mu_{ij}^*\right)^2}\right)$$

$$=N\left(0,\tau_{X_{n_i}}^2=\frac{\sum_j{\mu_{ij}^*}^2}{\phi\left(\lambda+\sum_j\mu_{ij}^*\right)^2}\right).$$

where $\tau_{X_{n_i}}^2$ is the variance of the normal distribution. For simplicity but without loss of generality, we first consider the null model where $\mu_{ij}^*=\pi_{ij}\exp(\beta_0)$ has only the intercept term and all variation of y_{ij} is included in ϕ and σ^2. In this case, $\tau_{X_{n_i}}^2$ can be expressed as

$$\tau_{X_{n_i}}^2=\frac{\exp(2\beta_0)\sum_j{\pi_{ij}}^2}{\phi\left(\lambda^2+2\lambda\exp(\beta_0)\sum_j\pi_{ij}+\exp(2\beta_0)(\sum_j\pi_{ij})^2\right)}.$$

Denote by $\pi=E(\pi_{ij})$ the mean of the scaling factor, and by $c=\sqrt{Var(\pi_{ij})}\big/\pi$ the coefficient of variation of the scaling factor. We found that c^2 is often small (~0.25) in most cell types (microglia, oligodendrocytes, astrocytes, and OPCs) in e.g., the snRNA-seq data in [5] when the total library size of a cell is used as the scaling factor. In excitatory and inhibitory neurons, we observed $c^2\approx 0.8$, probably because different types of neurons varied significantly in terms of morphology. Then, when n_i is large, we can approximately rewrite $\tau_{X_{n_i}}^2$ by plugging in π and c as

$$\tau_{X_{n_i}}^2=\frac{\exp(2\beta_0)\sum_j{\pi_{ij}}^2\big/{n_i}^2}{\phi\left(\lambda^2+2\lambda\exp(\beta_0)\sum_j\pi_{ij}+\exp(2\beta_0)\left(\sum_j\pi_{ij}\right)^2\right)\big/{n_i}^2}$$

$$\approx \frac{\exp(2\beta_0)\left(Var(\pi_{ij}) + \pi^2\right)\Big/_{n_i}}{\phi\left(\frac{\lambda^2}{{n_i}^2} + \frac{2\lambda\exp(\beta_0)\,\pi}{n_i} + \exp(2\beta_0)\pi^2\right)}$$

$$= \frac{1}{\frac{\phi n_i}{1+c^2} + \frac{2\lambda\phi}{(1+c^2)\exp(\beta_0)\,\pi} + \frac{\phi\lambda^2}{(1+c^2)\exp(2\beta_0)\,\pi^2 n_i}}$$

$$= \frac{(1+c^2)}{\phi n_i + \frac{2\lambda\phi}{\exp(\beta_0)\,\pi} + \frac{\phi\lambda^2}{\exp(2\beta_0)\,\pi^2 n_i}}.$$

Thus, the leading term in $\tau^2_{X_{n_i}}$ is $\frac{\phi n_i}{1+c^2}$ when $n_i \to \infty$. It is also clear that the second term can be large for low-expressed genes because of $\exp(\beta_0)\,\pi \ll 1$. Compared to the brain snRNA-seq data, some covariates such as ribosomal and mitochondrial mRNA abundance in scRNA-seq data in e.g., peripheral blood mononuclear cells can have very large effect sizes. In this case, we should take into account the contribution of these covariates as well when deriving and using the asymptotic variance $\tau^2_{X_{n_i}}$. Consider an alternative model $\mu^*_{ij} = \pi_{ij}\exp\left(\beta_0 + \sum_k \boldsymbol{x}_{ij}\beta_k\right) = \pi_{ij}\exp\left(\sum_k \boldsymbol{x}_{ij}\beta_k\right)\exp(\beta_0)$. After replacing with $\pi = E\left(\pi_{ij}\exp\left(\sum_k \boldsymbol{x}_{ij}\beta_k\right)\right)$ and $c = \sqrt{Var\left(\pi_{ij}\exp\left(\sum_k \boldsymbol{x}_{ij}\beta_k\right)\right)}\Big/_{\pi}$ in the above derivation, we end up with the same formula for $\tau^2_{X_{n_i}}$.

A.7. The Newton-Raphson algorithm for optimizing the h-likelihood

Here, we derive the NR algorithm for optimization of the h-likelihood in NEBULA-HL, which requires calculating the first and second derivatives of the h-likelihood. After parametrizing using $\eta_i = \log(\omega_i)$ in eq. (10), we obtain the following h-likelihood

$$\begin{aligned} hl(\boldsymbol{\beta},\boldsymbol{\eta}|\sigma^2,\phi) &= \sum_i hl_i(\boldsymbol{\beta},\eta_i|\sigma^2,\phi) \\ &= \sum_i\sum_j y_{ij}\left(\boldsymbol{x}_{ij}\boldsymbol{\beta} + \log(\pi_{ij}) + \eta_i\right) - (y_{ij}+\phi)\log\left(\phi + \exp\left(\boldsymbol{x}_{ij}\boldsymbol{\beta} + \log(\pi_{ij}) + \eta_i\right)\right) \\ &+ \alpha\eta_i - \lambda\exp(\eta_i), \end{aligned}$$

in which $\boldsymbol{\beta}$ and $\boldsymbol{\eta}$ are now on the canonical scale, and thus can be optimized simultaneously. For readability, we introduce the definition $\mu^{**}_{ij} = \exp\left(\boldsymbol{x}_{ij}\boldsymbol{\beta} + \log(\pi_{ij}) + \eta_i\right)$. Taking the first derivative with respect to $\boldsymbol{\beta}$ and η_i gives

$$\frac{\partial hl(\boldsymbol{\beta},\boldsymbol{\eta}|\sigma^2,\phi)}{\partial\boldsymbol{\beta}} = \sum_i\sum_j\left(y_{ij} - \frac{(y_{ij}+\phi)\mu^{**}_{ij}}{\phi+\mu^{**}_{ij}}\right)\boldsymbol{x}_{ij}$$

$$\frac{\partial hl(\boldsymbol{\beta},\boldsymbol{\eta}|\sigma^2,\phi)}{\partial\eta_i} = \sum_j\left(y_{ij} - \frac{(y_{ij}+\phi)\mu^{**}_{ij}}{\phi+\mu^{**}_{ij}}\right) + \alpha - \lambda\exp(\eta_i).$$

Taking the derivative of $\frac{\partial hl_i(\boldsymbol{\beta},\eta_i|\sigma^2,\phi)}{\partial \boldsymbol{\beta}}$ and $\frac{\partial hl_i(\boldsymbol{\beta},\eta_i|\sigma^2,\phi)}{\partial \eta_i}$ again with respect to $\boldsymbol{\beta}$ and η_i gives the Hessian matrix $\boldsymbol{H}$ with the following entries

$$\frac{\partial^2 hl(\boldsymbol{\beta},\boldsymbol{\eta}|\sigma^2,\phi)}{\partial\beta_s\partial\beta_t} = \sum_i\sum_j - z_{ij}x_{ijs}x_{ijt}$$

$$\frac{\partial^2 hl(\boldsymbol{\beta},\boldsymbol{\eta}|\sigma^2,\phi)}{\partial{\eta_i}^2} = -\lambda\exp(\eta_i) - \sum_j z_{ij}$$

$$\frac{\partial^2 hl(\boldsymbol{\beta},\boldsymbol{\eta}|\sigma^2,\phi)}{\partial\beta_s\partial\eta_i} = \sum_j - z_{ij}x_{ijs}\eta_i$$

where $z_{ij} = \frac{\phi(y_{ij}+\phi)\mu_{ij}^{**}}{\left(\phi+\mu_{ij}^{**}\right)^2}$. And all second derivatives $\frac{\partial^2 hl_i(\boldsymbol{\beta},\eta_i|\sigma^2,\phi)}{\partial\eta_i\partial\eta_j}$ equal zero. Therefore, $\boldsymbol{H}$ is highly sparse and the bottom-right block matrix is diagonal. Then, an NR algorithm updates $(\boldsymbol{\beta},\boldsymbol{\eta})$ at each iteration using

$$(\boldsymbol{\beta}^{new},\boldsymbol{\eta}^{new})^T = (\boldsymbol{\beta}^{old},\boldsymbol{\eta}^{old})^T - \boldsymbol{H}^{-1}\left(\frac{\partial hl(\boldsymbol{\beta},\boldsymbol{\eta}|\sigma^2,\phi)}{\partial\boldsymbol{\beta}},\frac{\partial hl(\boldsymbol{\beta},\boldsymbol{\eta}|\sigma^2,\phi)}{\partial\eta_i}\right)^T$$

until the increase of the h-likelihood is smaller than a pre-defined tolerance parameter and reaches convergence.

A.8. The Hessian matrix for the Newton-Raphson algorithm in NEBULA-LN

We derive an NR algorithm for optimizing $\tilde{l}_i(\boldsymbol{\beta},\sigma^2,\phi)$ in NEBULA-LN. The first derivatives of $\tilde{l}_i(\boldsymbol{\beta},\sigma^2,\phi)$ are given in A.4. It remains to calculate the Hessian matrix by taking the second derivatives. We use an approximation for some terms by taking the expectation over y_{ij} to simplify the results. For the sake of readability, it is convenient to introduce the following definitions: $\phi_{ij}^* := \phi + \breve{\omega}_i\mu_{ij}^*$, $\bar{\boldsymbol{x}}_i := {\boldsymbol{\mu}_i^*}^T\boldsymbol{X}_i$, and $\bar{\boldsymbol{X}}_i := {\boldsymbol{X}_i}^T\text{diag}(\boldsymbol{\mu}_i^*)\boldsymbol{X}_i$. We denote by α'' and λ'' the second derivative of α and λ with respect to σ^2. Through some calculations, it follows that

$$\begin{aligned}\frac{\partial^2\tilde{l}_i(\boldsymbol{\beta},\sigma^2,\phi)}{\partial\beta_s\partial\beta_t} = &-\frac{{\breve{\omega}_i}^2\bar{x}_{is}}{\check{\mu}_i}\sum_j\frac{\breve{v}_{ij}{\mu_{ij}^*}^2x_{ijt}}{\phi_{ij}^*} - \frac{{\breve{\omega}_i}^2\bar{x}_{it}}{\check{\mu}_i}\sum_j\frac{\breve{v}_{ij}{\mu_{ij}^*}^2x_{ijs}}{\phi_{ij}^*} + \frac{{\breve{\omega}_i}^2\bar{x}_{is}\bar{x}_{it}}{{\check{\mu}_i}^2}\sum_j\frac{\breve{v}_{ij}{\mu_{ij}^*}^2}{\phi_{ij}^*}\\ &-\breve{\omega}_i\sum_j\breve{v}_{ij}\mu_{ij}^*x_{ijs}\,x_{ijt} + \frac{\breve{\omega}_i\bar{x}_{it}}{\check{\mu}_i}\sum_j\breve{v}_{ij}\mu_{ij}^*x_{ijs} + \frac{\breve{\omega}_i\bar{x}_{is}}{\check{\mu}_i}\sum_j\breve{v}_{ij}\mu_{ij}^*x_{ijt}\\ &+{\breve{\omega}_i}^2\sum_j\frac{\breve{v}_{ij}{\mu_{ij}^*}^2x_{ijs}x_{ijt}}{\phi_{ij}^*} - \frac{\breve{\omega}_i\bar{x}_{is}\bar{x}_{it}}{\check{\mu}_i} + \frac{\breve{\omega}_i\bar{\boldsymbol{X}}_{ist}}{\check{\mu}_i}\sum_j(1-\breve{v}_{ij})\mu_{ij}^*\\ &+\frac{2\breve{\omega}_i\bar{x}_{is}\bar{x}_{it}}{{\check{\mu}_i}^2}\sum_j(1-\breve{v}_{ij})\mu_{ij}^*\end{aligned}$$

$$\frac{\partial^2 \tilde{l}_i(\boldsymbol{\beta}, \sigma^2, \phi)}{\partial \phi^2} = \sum_j \frac{1}{\phi} + \Psi'(y_{ij} + \phi) - \Psi'(\phi) - \frac{2 - \breve{v}_{ij}}{\phi_{ij}^*}$$

$$\frac{\partial^2 \tilde{l}_i(\boldsymbol{\beta}, \sigma^2, \phi)}{\partial \sigma^4} = \alpha'' \log \lambda + \frac{2\alpha'\lambda'}{\lambda} - \frac{\alpha\lambda'^2}{\lambda^2} + \frac{\alpha\lambda''}{\lambda} + \alpha''\left(\Psi\left(\sum_j y_{ij} + \alpha\right) - \Psi(\alpha)\right)$$
$$+ \alpha'\left(\Psi'\left(\sum_j y_{ij} + \alpha\right) - \Psi'(\alpha)\right) - \frac{2\alpha'\lambda'}{\breve{\mu}_i} - \alpha'' \log \breve{\mu}_i - \lambda''\breve{\omega}_i + \frac{\lambda'^2 \breve{\omega}_i}{\breve{\mu}_i}$$
$$+ \left(\frac{\alpha' - \lambda'\breve{\omega}_i}{\breve{\mu}_i}\right)^2 \sum_j \frac{\breve{v}_{ij}\mu_{ij}^{*\,2}}{\phi_{ij}^*} + \left(\frac{\alpha'' - \lambda''\breve{\omega}_i}{\breve{\mu}_i} - \frac{2\alpha'\lambda' - 2\lambda'^2\breve{\omega}_i}{\breve{\mu}_i^{\,2}}\right) \sum_j (1 - \breve{v}_{ij})\mu_{ij}^*$$

$$\frac{\partial^2 \tilde{l}_i(\boldsymbol{\beta}, \sigma^2, \phi)}{\partial \phi \partial \sigma^2} = -\frac{\alpha' - \lambda'\breve{\omega}_i}{\breve{\mu}_i} \sum_j \frac{(1 - \breve{v}_{ij})\mu_{ij}^*}{\phi_{ij}^*}$$

$$\frac{\partial^2 \tilde{l}_i(\boldsymbol{\beta}, \sigma^2, \phi)}{\partial \phi \partial \beta_s} = -\breve{\omega}_i \sum_j \frac{(1 - \breve{v}_{ij})\mu_{ij}^* x_{ijs}}{\phi_{ij}^*} - \frac{\breve{\omega}_i \bar{x}_{is}}{\breve{\mu}_i} \sum_j \frac{(1 - \breve{v}_{ij})\mu_{ij}^*}{\phi_{ij}^*}$$

$$\frac{\partial^2 \tilde{l}_i(\boldsymbol{\beta}, \sigma^2, \phi)}{\partial \sigma^2 \partial \beta_s} \approx -\frac{\alpha'}{\breve{\mu}_i} \sum_j \breve{v}_{ij}\mu_{ij}^* x_{ijs} + \frac{\lambda'\breve{\omega}_i}{\breve{\mu}_i} \sum_j \breve{v}_{ij}\mu_{ij}^* x_{ijs}$$
$$+ \left(\frac{\alpha' - \lambda'\breve{\omega}_i}{\breve{\mu}_i}\right) \breve{\omega}_i \left(\sum_j \frac{\breve{v}_{ij}\mu_{ij}^{*\,2} x_{ijs}}{\phi_{ij}^*} - \frac{\bar{x}_{is}}{\breve{\mu}_i} \sum_j \frac{\breve{v}_{ij}\mu_{ij}^{*\,2}}{\phi_{ij}^*}\right),$$

where $\Psi'(x) = \frac{d^2 \log(\Gamma(x))}{dx^2}$ is the trigamma function, and $\frac{\partial^2 \tilde{l}_i(\boldsymbol{\beta}, \sigma^2, \phi)}{\partial \sigma^2 \partial \beta_s}$ is approximated by plugging in $\frac{1}{\breve{\mu}_i}\sum_j (1 - \breve{v}_{ij})\mu_{ij}^* \approx 0$.

A.9. The higher-order Laplace approximation in NEBULA-HL

We find that the first-order LA is not sufficient for the NBGMM to obtain an accurate estimate of the subject-level overdispersion σ^2 when the count variable is highly sparse or σ^2 is relatively large (Supplementary Fig. S17). For example, when the count per subject of a gene is ≤2, NEBULA-HL using the first-order LA underestimates σ^2 with a substantial bias. This issue arises because the h-likelihood of the NBGMM becomes highly skewed when almost all of the counts are zero (Supplementary Fig. S18). The skewness results from the penalizing term in the h-likelihood, which, unlike the NBLMM, is an exponential function rather than quadratic. Therefore, the first-order LA, which relies on a Gaussian distribution to approximate the integral, produces a large bias under this setting. In contrast, this issue is less serious in the NBLMM because its distribution of the random effects is quadratic (See Supplementary Fig. S18).

To improve the performance of estimating σ^2 for low-expressed genes, we develop an efficient higher-order LA method for NEBULA-HL when the non-zero count per subject is <4. This method includes second-order and higher-order terms in the multivariate Taylor expansion to correct for the skewness. Following the notation used in [6], we modify the marginal log-likelihood in eq. (12) into

$$l^h(\sigma^2, \phi|\boldsymbol{\beta}^*, \boldsymbol{\eta}^*) = hl(\boldsymbol{\beta}^*, \boldsymbol{\eta}^*|\sigma^2, \phi) - \frac{1}{2}\log\left(\left|\frac{\partial^2 hl(\boldsymbol{\beta}, \boldsymbol{\eta}|\sigma^2, \phi)}{\partial \boldsymbol{\eta}^{*2}}\right|\right) + \log(\boldsymbol{\Lambda}),$$

where $\boldsymbol{\Lambda} = 1 + E(T_4) + E(T_3T_5) + E(T_3{}^2)/2$ is a correction including three higher-order terms in the Taylor expansion of the exponential of the Taylor expansion of the h-likelihood in eq. (11). The evaluation of $E(T_4)$, $E(T_3T_5)$, and $E(T_3{}^2)$ is given in the next paragraph. The optimization algorithm in NEBULA-HL for low-expressed genes thus iterates between the h-likelihood in eq. (11) and the modified marginal log-likelihood $l^h(\sigma^2, \phi|\boldsymbol{\beta}^*, \boldsymbol{\eta}^*)$. In this algorithm, we use the h-likelihood to estimate $\boldsymbol{\beta}$ and $\boldsymbol{\eta}$, and then use $l^h(\sigma^2, \phi|\boldsymbol{\beta}^*, \boldsymbol{\eta}^*)$ to estimate σ^2 and ϕ. This is conceptually equivalent to the method named as $HL(0,2)$ in [7] except for a minor modification. The difference is that $HL(0,2)$ evaluates the derivative with respect to both $\boldsymbol{\beta}$ and $\boldsymbol{\eta}$ in $l^h(\sigma^2, \phi|\boldsymbol{\beta}^*, \boldsymbol{\eta}^*)$, while our method computes the derivative with respect to η_i only. This modification can be justified by two facts. First, as the sample size of single-cell data is generally large, ignoring the uncertainty of the estimate of $\boldsymbol{\beta}$ practically has little effect on estimating σ^2 and ϕ. Second, omitting the derivative with respect to $\boldsymbol{\beta}$ will dramatically simplify the calculation of those higher-order terms in $\boldsymbol{\Lambda}$ (i.e., $E(T_4)$, $E(T_3{}^2)$, etc) as we show in the following derivation.

Briefly, the higher-order LA method first rewrites the h-likelihood $hl(\boldsymbol{\beta}, \boldsymbol{\eta}|\sigma^2, \phi)$ in eq. (11) using the Taylor expansion. If we view the second-order term $T_2 = \frac{\partial^2 hl(\boldsymbol{\beta}, \boldsymbol{\eta}|\sigma^2, \phi)}{\partial \boldsymbol{\eta}^{*2}} \frac{(\boldsymbol{\eta}-\boldsymbol{\eta}^*)^2}{2}$ in the Taylor expansion as a kernel of the normal distribution, the marginal likelihood in eq. (10) is proportional to the expectation of the exponential of the third- and higher-order terms, denoted by T_3, T_4, and so on, under the normal distribution. By applying the Taylor expansion to the expectation again, we can rewrite the marginal likelihood as

$$L(\sigma^2, \phi|\boldsymbol{\beta}^*, \boldsymbol{\eta}^*) = \exp\left(hl(\boldsymbol{\beta}^*, \boldsymbol{\eta}^*|\sigma^2, \phi) - \frac{1}{2}\log\left(\left|\frac{\partial^2 hl(\boldsymbol{\beta}, \boldsymbol{\eta}|\sigma^2, \phi)}{\partial \boldsymbol{\eta}^{*2}}\right|\right)\right) E(1 + R + \frac{R^2}{2!} + \cdots),$$

where $R = \sum_{i\geq 3}^{\infty} T_i$ and $E(\cdot)$ is the expectation over $\boldsymbol{\eta}$. The detailed derivation of the higher-order multivariate LA can be found in e.g., [6], the first equation on p. 89 in [9], or Chapter 6 in [8]. Hence the marginal log-likelihood becomes

$$\log\big(L(\sigma^2, \phi|\boldsymbol{\beta}^*, \boldsymbol{\eta}^*)\big) = hl(\boldsymbol{\beta}^*, \boldsymbol{\eta}^*|\sigma^2, \phi) - \frac{1}{2}\log\left(\left|\frac{\partial^2 hl(\boldsymbol{\beta}, \boldsymbol{\eta}|\sigma^2, \phi)}{\partial \boldsymbol{\eta}^{*2}}\right|\right) + \log\left(1 + E(\mathrm{R}) + E\left(\frac{R^2}{2!}\right) + \cdots\right),$$

and keeping the first few items gives $l^h(\sigma^2, \phi|\boldsymbol{\beta}^*, \boldsymbol{\eta}^*)$. In principle, we can achieve any accuracy as much as we want by adding more higher-order terms T_i. On the other hand, including more terms requires the additional computation of higher-order multivariate derivatives. We find that including $E(T_4)$, $E(T_3{}^2)/2$, and $E(T_3T_5) = \frac{E(T_3T_5)+E(T_5T_3)}{2}$ works practically well as a trade-off between accuracy and efficiency, and already achieves a substantial improvement in reducing the bias of estimating σ^2 for the NBGMM (Supplementary Fig. S17). Taking the logarithm of $L(\sigma^2, \phi|\boldsymbol{\beta}^*, \boldsymbol{\eta}^*)$ by including $E(T_4)$, $E(T_3{}^2)/2$, and $E(T_3T_5)$ leads to $\sum_i {l^h}_i(\sigma^2, \phi|\boldsymbol{\beta}^*, \boldsymbol{\eta}^*)$.

Next, we turn to the calculation of the terms $E(T_4)$, $E\left({T_3}^2\right)$, and $E(T_3T_5)$ in $\boldsymbol{\Lambda}$. As $\frac{\partial^k hl(\boldsymbol{\beta},\boldsymbol{\eta}|\sigma^2,\phi)}{\partial \boldsymbol{\eta}^{*k}}$ $(k = 3,4,5)$ are all constants, the expectation is equivalent to the higher-order joint moments of a zero-mean normal distribution, the calculation of which follows from Isserlis' theorem (see e.g, [9], p. 85). More specifically, we have the following formulae in general

$$E(T_4) = -\frac{h_{ijkl}h^{ij}h^{kl}}{8}$$

$$E\left({T_3}^2\right) = \frac{9h_{ijk}h_{lmn}h^{ij}h^{kl}h^{mn} + 6h_{ijk}h_{lmn}h^{il}h^{jm}h^{kn}}{36}$$

$$E(T_3T_5) = \frac{45h_{ijk}h_{lmnop}h^{ij}h^{kl}h^{mn}h^{op}+60h_{ijk}h_{lmnop}h^{il}h^{jm}h^{kn}h^{op}}{720},$$

where $h_{ijkl}h^{ij}h^{kl} = \sum_{i=1}^{m}\sum_{j=1}^{m}\sum_{k=1}^{m}\sum_{l=1}^{m} h_{ijkl}h^{ij}h^{kl}$ (m is the number of subjects) uses the Einstein summation notation described in [9], $h_{ijk}, h_{ijkl}, h_{lmnop}$ are the corresponding elements in the third, fourth, and fifth derivatives of the negative h-likelihood, and h^{ij} is the corresponding element in the inverse of $-\frac{\partial^2 hl(\boldsymbol{\beta},\boldsymbol{\eta}|\sigma^2,\phi)}{\partial \boldsymbol{\eta}^{*2}}$. The coefficients in the numerator of these formulae can be derived using Table 1 of the Appendix of [9]. Note that another option can be further expressing the log of the expectation $\log\left(E\left(1 + R + \frac{R^2}{2!} + \cdots\right)\right)$ in terms of the cumulant generating function as proposed in [9,10]. This method requires counting complementary partitions (that is, only those complementary partitions should be included in the numerator, although there is no difference for the three terms that we consider here). Because the Hessian matrix is diagonal ($h_{ij} = 0$ if $i \neq j$) in our model, these formulae then simplify to

$$E(T_4) = -\frac{h_{iiii}h^{ii}h^{ii}}{8}$$

$$E\left({T_3}^2\right) = \frac{5h_{iii}h_{iii}h^{ii}h^{ii}h^{ii}}{24}$$

$$E(T_3T_5) = \frac{7h_{iii}h_{iiiii}h^{ii}h^{ii}h^{ii}h^{ii}}{48}.$$

The same results can also be reached by using Theorem 2 in [6]. Hence, the major additional computational burden in this higher-order LA is to calculate the higher-order derivatives h_{iii}, h_{iiii}, and h_{iiiii}, which takes $O(n)$ time. It follows through a straightforward calculation based on $\frac{\partial^2 hl(\boldsymbol{\beta},\boldsymbol{\eta}|\sigma^2,\phi)}{\partial {\eta_i}^2}$ in A.7 that

$$h_{iii} = \frac{\partial^3 hl(\boldsymbol{\beta},\boldsymbol{\eta}|\sigma^2,\phi)}{\partial {\eta_i}^3} = -\lambda\exp(\eta_i) - \sum_j \frac{\phi\left(y_{ij}+\phi\right)\mu_{ij}^{**}\left(\phi - \mu_{ij}^{**}\right)}{\left(\phi + \mu_{ij}^{**}\right)^3}$$

$$h_{iiii} = \frac{\partial^4 hl(\boldsymbol{\beta}, \boldsymbol{\eta} | \sigma^2, \phi)}{\partial {\eta_i}^4} = -\lambda \exp(\eta_i) - \sum_j \frac{\phi(y_{ij} + \phi)\mu_{ij}^{**}(\phi^2 - 4\phi\mu_{ij}^{**} + {\mu_{ij}^{**}}^2)}{(\phi + \mu_{ij}^{**})^4}$$

$$h_{iiiii} = \frac{\partial^5 hl(\boldsymbol{\beta}, \boldsymbol{\eta} | \sigma^2, \phi)}{\partial {\eta_i}^5} = -\lambda \exp(\eta_i) - \sum_j \frac{\phi(y_{ij} + \phi)\mu_{ij}^{**}(\phi^3 - 11\phi^2\mu_{ij}^{**} + 11\phi{\mu_{ij}^{**}}^2 - {\mu_{ij}^{**}}^3)}{(\phi + \mu_{ij}^{**})^5}.$$

References

1. Huber, P. J. *Robust Statistics*. (John Wiley & Sons, 2004).
2. Vaart, A. W. van der. *Asymptotic Statistics*. (Cambridge University Press, 1998). doi:10.1017/CBO9780511802256.
3. Serfling, R. J. *Approximation Theorems of Mathematical Statistics*. (John Wiley & Sons, 2009).
4. Sutradhar, B. C. & Qu, Z. On approximate likelihood inference in a poisson mixed model. *Can. J. Stat.* **26**, 169–186 (1998).
5. Mathys, H. *et al.* Single-cell transcriptomic analysis of Alzheimer's disease. *Nature* **570**, 332–337 (2019).
6. Raudenbush, S. W., Yang, M.-L. & Yosef, M. Maximum Likelihood for Generalized Linear Models with Nested Random Effects via High-Order, Multivariate Laplace Approximation. *J. Comput. Graph. Stat.* **9**, 141–157 (2000).
7. Noh, M. & Lee, Y. REML estimation for binary data in GLMMs. *J. Multivar. Anal.* **98**, 896–915 (2007).
8. Barndorff-Nielsen, O. E., Cox, D. R. & Cox, H. F. D. R. *Asymptotic Techniques for Use in Statistics*. (Springer US, 1989).
9. McCullagh, P. *Tensor Methods in Statistics*. (Courier Dover Publications, 2018).
10. Shun, Z. & McCullagh, P. Laplace approximation of high dimensional integrals. *J. R. Stat. Soc. Ser. B Methodol.* **57**, 749–760 (1995).

Supplementary Figures

Supplementary Figure S1. The computational time (measured in $\log_{10}$(seconds)) of fitting an NBMM by NEBULA, *glmer.nb*, and *glmmTMB* for 10,000 genes with respect to the number of fixed-effects variables included in the model. The number of subjects was set at 50, and CPS was set at 100. The average benchmarks are summarized from scenarios of varying subject-level and cell-level overdispersions and the mean count per cell ranging from exp(-4) to 1.

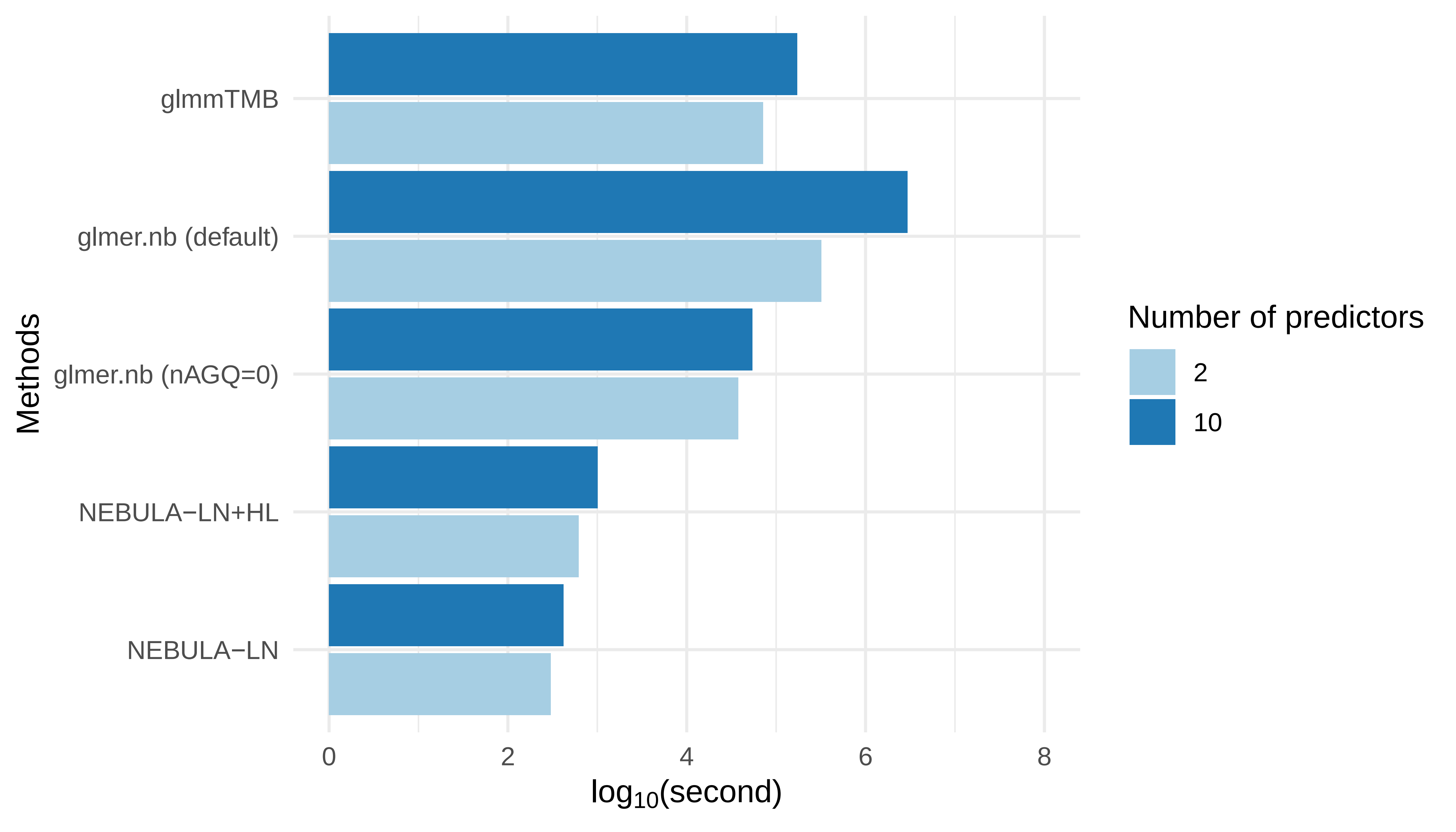
glmmTMB
glmer.nb (default)
glmer.nb (nAGQ=0)
NEBULA−LN+HL
NEBULA−LN
Methods
0
2
4
6
8
log10(second)
Number of predictors
2
10

Supplementary Figure S2. Comparison of estimated cell-level and subject-level overdispersions between NEBULA-LN and NEBULA-HL under a situation in which the CV of the scaling factor (π_{ij}) is one. The summary statistics were calculated from n=500 simulated replicates in each of the scenarios. (A) The cell-level overdispersion estimated by NEBULA-LN and NEBULA-HL under different combinations of CPS, β_0 (a lower β_0 corresponding to a lower CPC value), and ϕ. The number of subjects was set at 50. (B) The subject-level overdispersion estimated by NEBULA-LN and NEBULA-HL under different combinations of β_0, σ^2, and ϕ. The number of subjects was set at 50. The CPS value was set at 400. (C) The subject-level overdispersion estimated by NEBULA-LN and NEBULA-HL under different combinations of the number of subjects, σ^2, and ϕ. The CPS value was set at 400, and β_0 was set at 0.05. (D) The subject-level overdispersion estimated by NEBULA-LN and NEBULA-HL under different combinations of CPS, σ^2, and ϕ. The number of subjects was set at 50, and β_0 was set at 1.

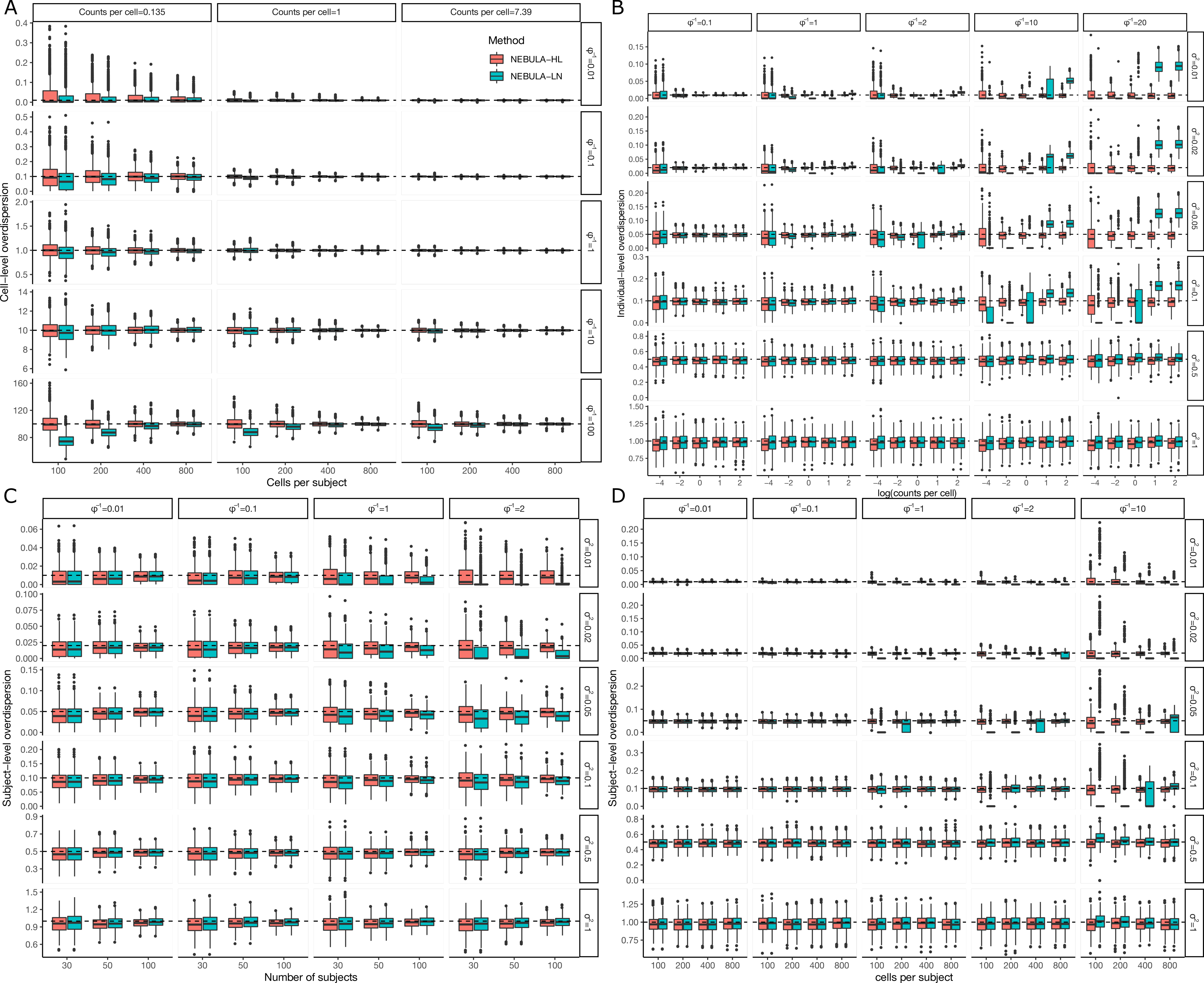
A
Counts per cell=0.135
Counts per cell=1
Counts per cell=7.39
Method
NEBULA-HL
NEBULA-LN
Cell-level overdispersion
Cells per subject
B
Individual-level overdispersion
log(counts per cell)
C
Subject-level overdispersion
Number of subjects
D
Subject-level overdispersion
cells per subject

Supplementary Figure S3. Comparison of estimated cell-level overdispersion between NEBULA-LN and NEBULA-HL under different combinations of the number of subjects, β_0, and ϕ. The CPS value was set at 200. The summary statistics were calculated from n=500 simulated replicates in each of the scenarios.

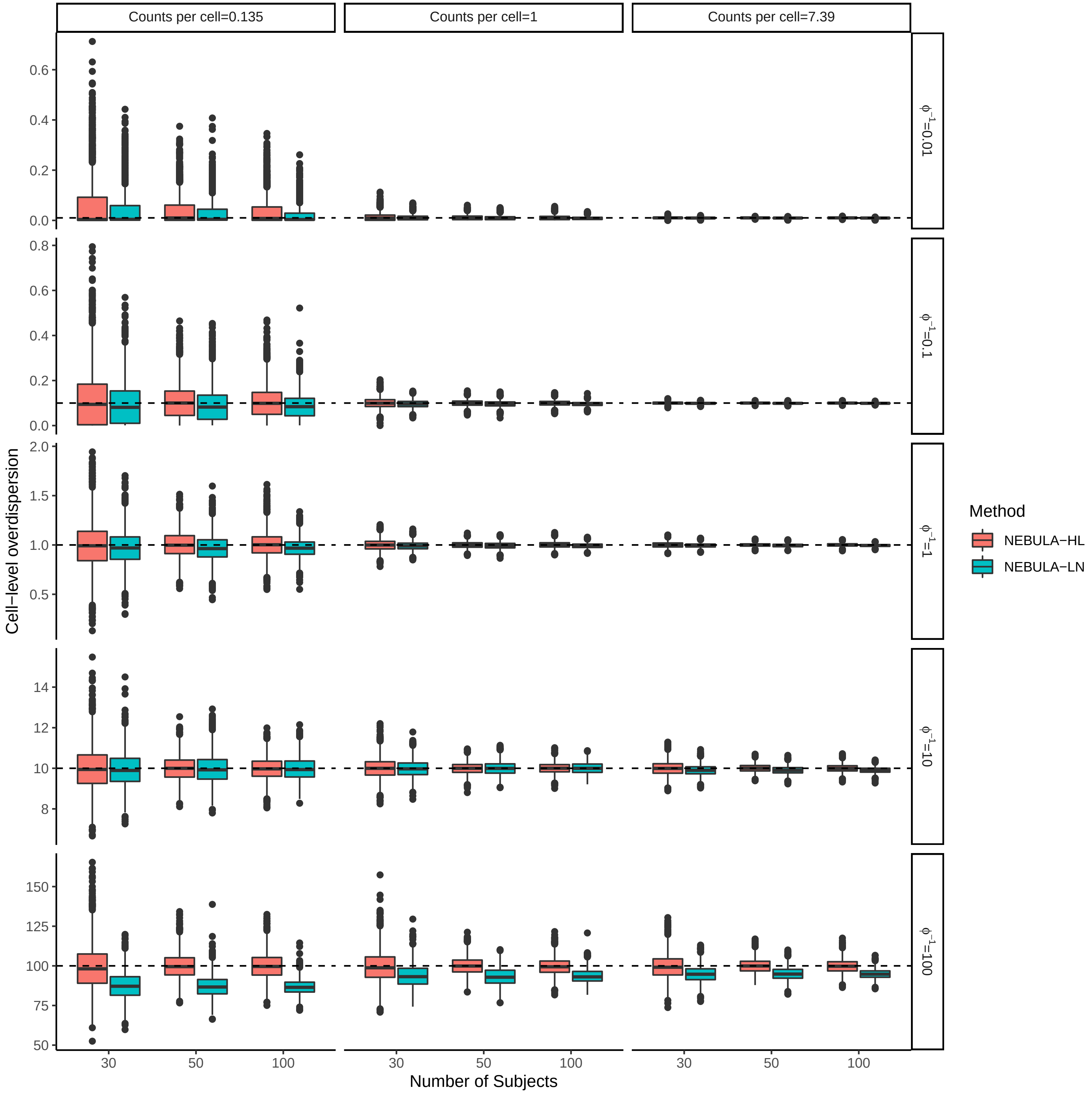
Counts per cell=0.135
Counts per cell=1
Counts per cell=7.39
ϕ^{-1}=0.01
ϕ^{-1}=0.1
ϕ^{-1}=1
ϕ^{-1}=10
ϕ^{-1}=100
Cell−level overdispersion
Number of Subjects
Method
NEBULA−HL
NEBULA−LN

Supplementary Figure S4. Comparison of estimated cell-level and subject-level overdispersions between NEBULA-LN and NEBULA-HL under an unbalanced design in which the cell counts across the subjects were sampled from a negative binomial distribution with size=3. The summary statistics were calculated from n=500 simulated replicates in each of the scenarios. (A) The cell-level overdispersion estimated by NEBULA-LN and NEBULA-HL under different combinations of CPS, β_0 (a lower β_0 corresponding to a lower CPC value), and ϕ. The number of subjects was set at 50. (B) The subject-level overdispersion estimated by NEBULA-LN and NEBULA-HL under different combinations of β_0, σ^2, and ϕ. The number of subjects was set at 50. The CPS value was set at 400. (C) The subject-level overdispersion estimated by NEBULA-LN and NEBULA-HL under different combinations of the number of subjects, σ^2, and ϕ. The CPS value was set at 400, and β_0 was set at 0.05. (D) The subject-level overdispersion estimated by NEBULA-LN and NEBULA-HL under different combinations of CPS, σ^2, and ϕ. The number of subjects was set at 50, and β_0 was set at 1.

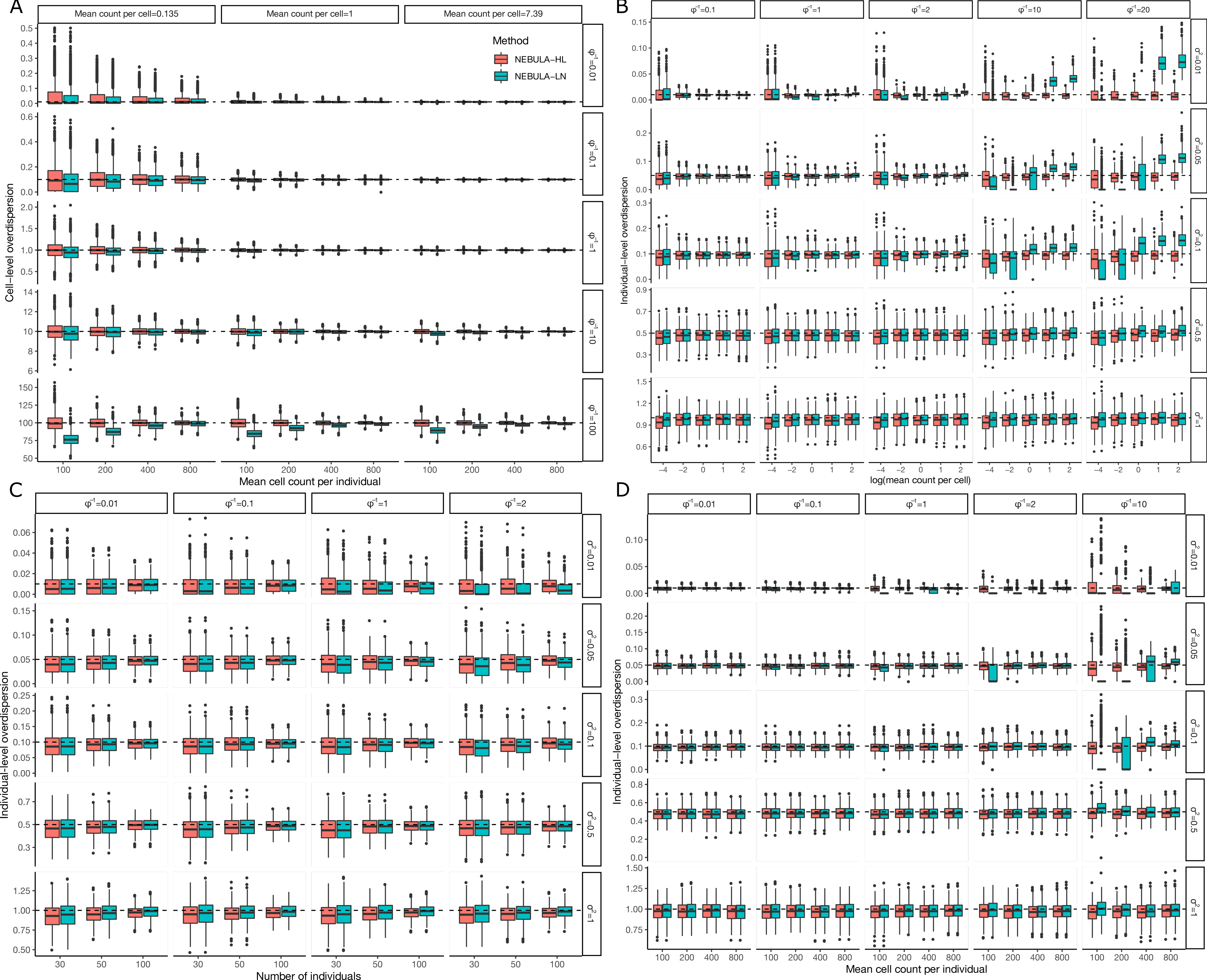
A
Mean count per cell=0.135
Mean count per cell=1
Mean count per cell=7.39
Method
NEBULA-HL
NEBULA-LN
φ⁻¹=0.01
φ⁻¹=0.1
φ⁻¹=1
φ⁻¹=10
φ⁻¹=100
Cell-level overdispersion
Mean cell count per individual
B
φ⁻¹=0.1
φ⁻¹=1
φ⁻¹=2
φ⁻¹=10
φ⁻¹=20
σ²=0.01
σ²=0.05
σ²=0.1
σ²=0.5
σ²=1
Individual-level overdispersion
log(mean count per cell)
C
φ⁻¹=0.01
φ⁻¹=0.1
φ⁻¹=1
φ⁻¹=2
Individual-level overdispersion
Number of individuals
D
φ⁻¹=0.01
φ⁻¹=0.1
φ⁻¹=1
φ⁻¹=2
φ⁻¹=10
Individual-level overdispersion
Mean cell count per individual

Supplementary Figure S5. Empirical type I error rate of testing a subject-level variable using NEBULA-LN under different combinations of CPC, σ^2, and ϕ. The CPS value was set at 400 and the number of subjects was set at 50. The empirical type I error rate was calculated from n=500 simulated replicates in each of the scenarios and was evaluated at the significance level of 0.05 (the dashed lines).

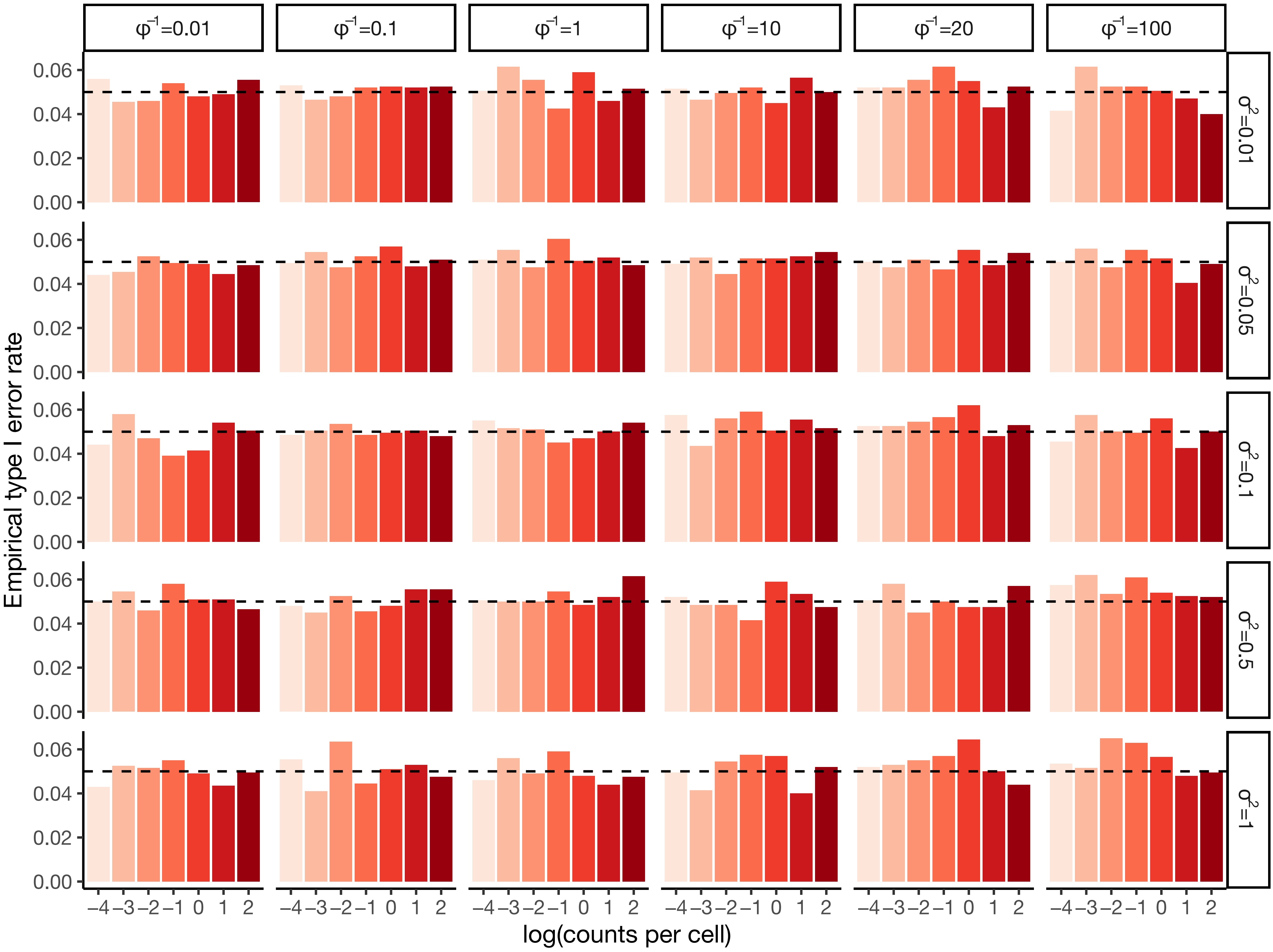
φ⁻¹=0.01
φ⁻¹=0.1
φ⁻¹=1
φ⁻¹=10
φ⁻¹=20
φ⁻¹=100
σ²=0.01
σ²=0.05
σ²=0.1
σ²=0.5
σ²=1
Empirical type I error rate
0.00
0.02
0.04
0.06
−4 −3 −2 −1 0 1 2
log(counts per cell)

Supplementary Figure S6. Empirical type I error rate of testing a cell-level variable using NEBULA-LN under different combinations of CPC, σ^2, and ϕ. The number of subjects was set at 50 and the CPS value was set at 400. The empirical type I error rate was calculated from n=500 simulated replicates in each of the scenarios and was evaluated at the significance level of 0.05 (the dashed lines).

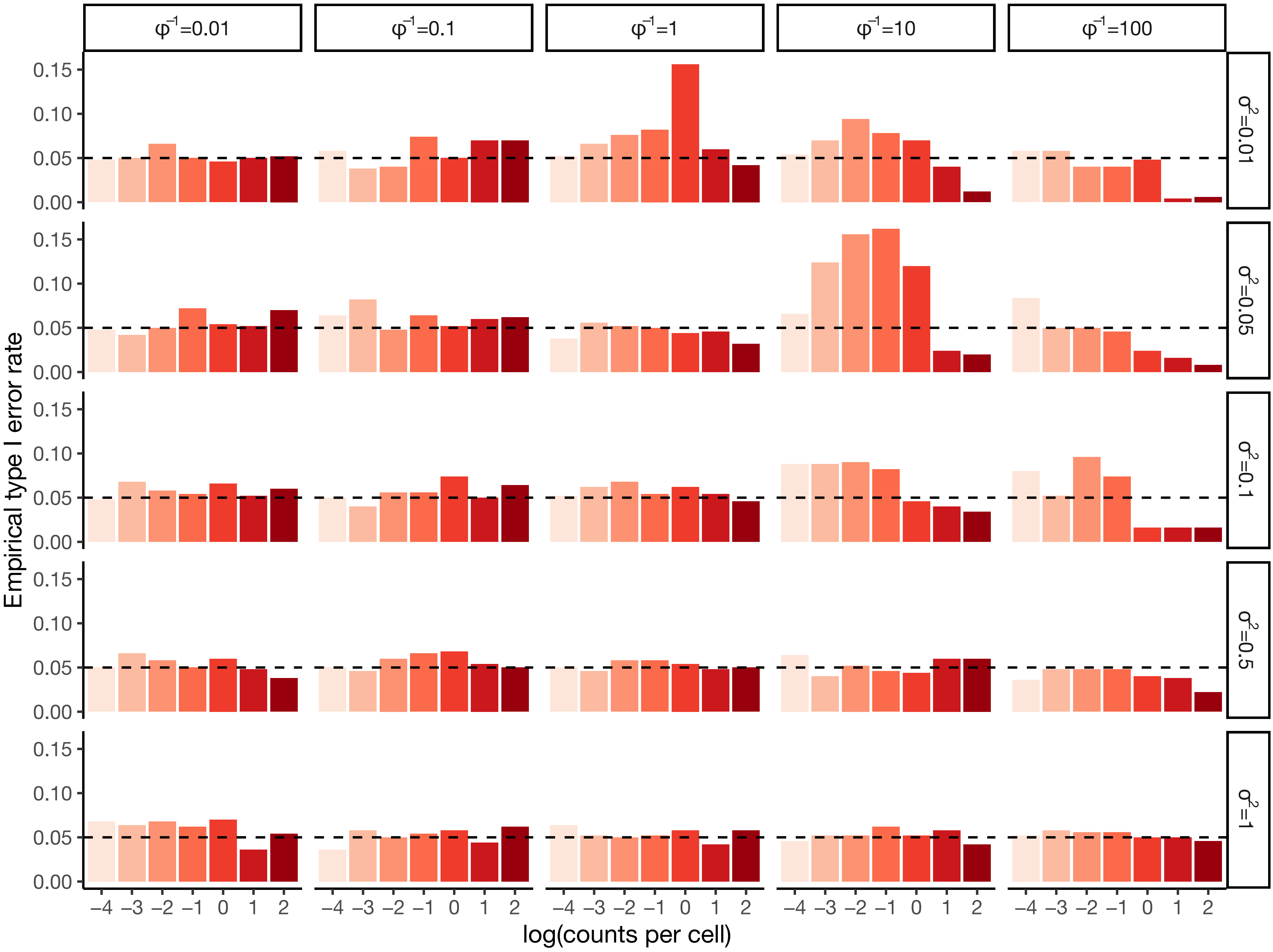

φ^-1=0.01
φ^-1=0.1
φ^-1=1
φ^-1=10
φ^-1=100
σ^2=0.01
σ^2=0.05
σ^2=0.1
σ^2=0.5
σ^2=1
0.15
0.10
0.05
0.00
Empirical type I error rate
−4 −3 −2 −1 0 1 2
log(counts per cell)

Supplementary Figure S7. Empirical type I error rate of testing a subject-level variable using PGMM under different combinations of the number of individuals, σ^2, and ϕ. The CPS value was set at 200. The empirical type I error rate was calculated from n=500 simulated replicates in each of the scenarios and was evaluated at the significance level of 0.05 (the dashed lines).

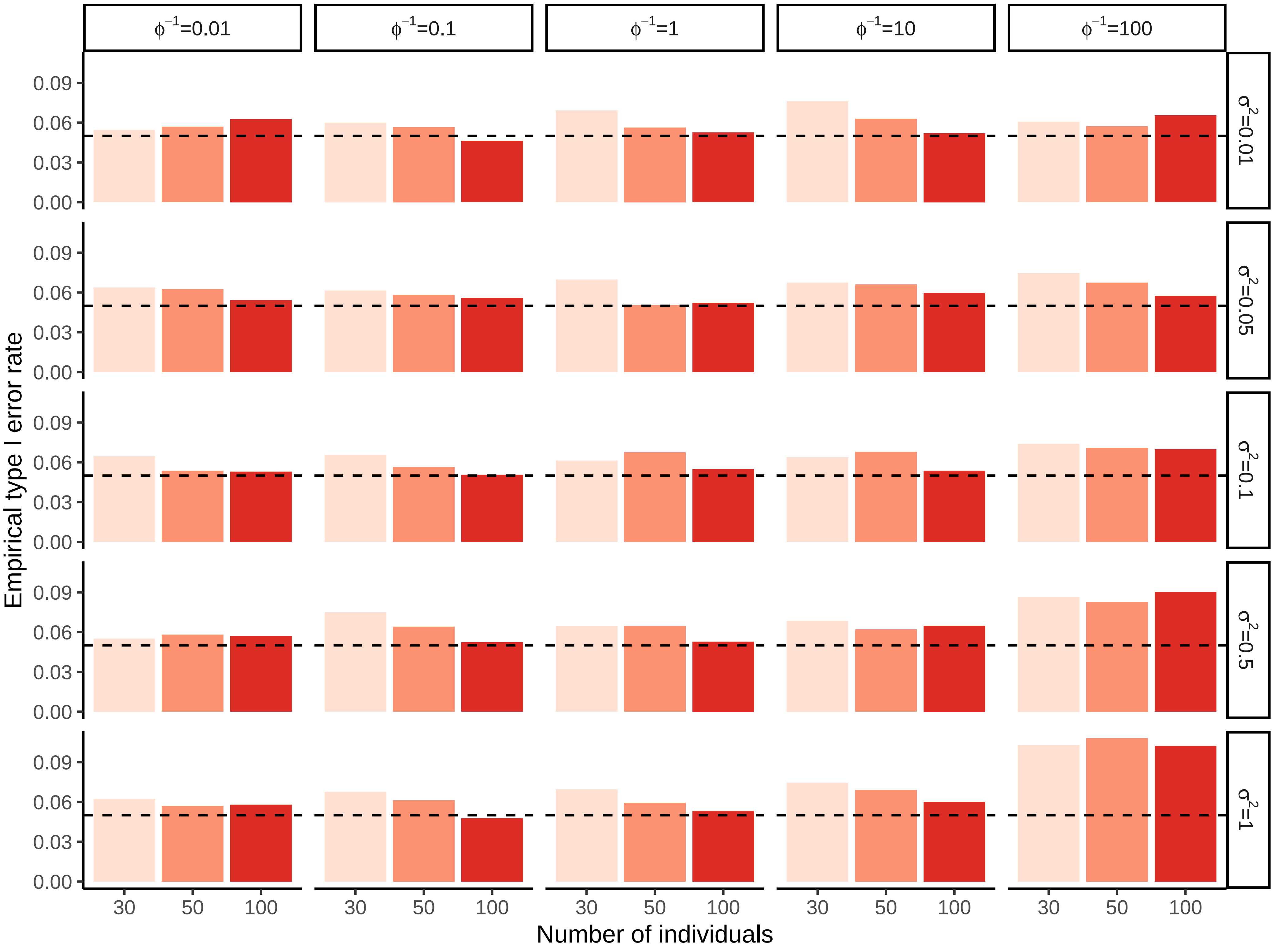

ϕ^−1=0.01
ϕ^−1=0.1
ϕ^−1=1
ϕ^−1=10
ϕ^−1=100
σ^2=0.01
σ^2=0.05
σ^2=0.1
σ^2=0.5
σ^2=1
Empirical type I error rate
0.00
0.03
0.06
0.09
30
50
100
Number of individuals

Supplementary Figure S8. Distribution of the library size (the total number of reads) of each cell grouped by the seven major cell types in the snRNA-seq data in the human frontal cortex.

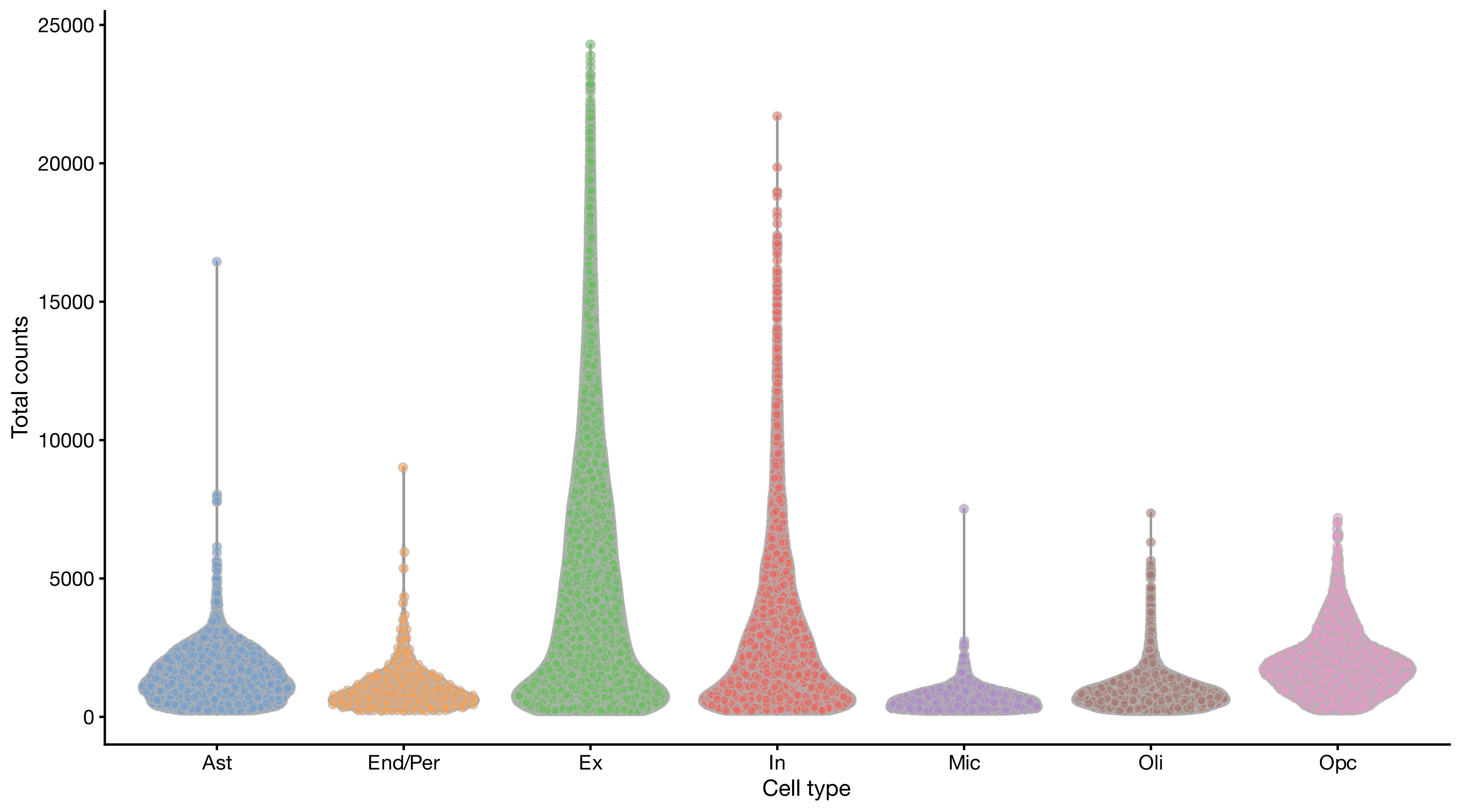

25000
20000
15000
10000
5000
0
Total counts
Ast
End/Per
Ex
In
Mic
Oli
Opc
Cell type

Supplementary Figure S9. The cell-level overdispersion of genes with CPC>0.1% in five major cell types in the frontal cortex estimated by NEBULA with respect to the CPC of the gene. No covariates other than the intercept were included in the model.

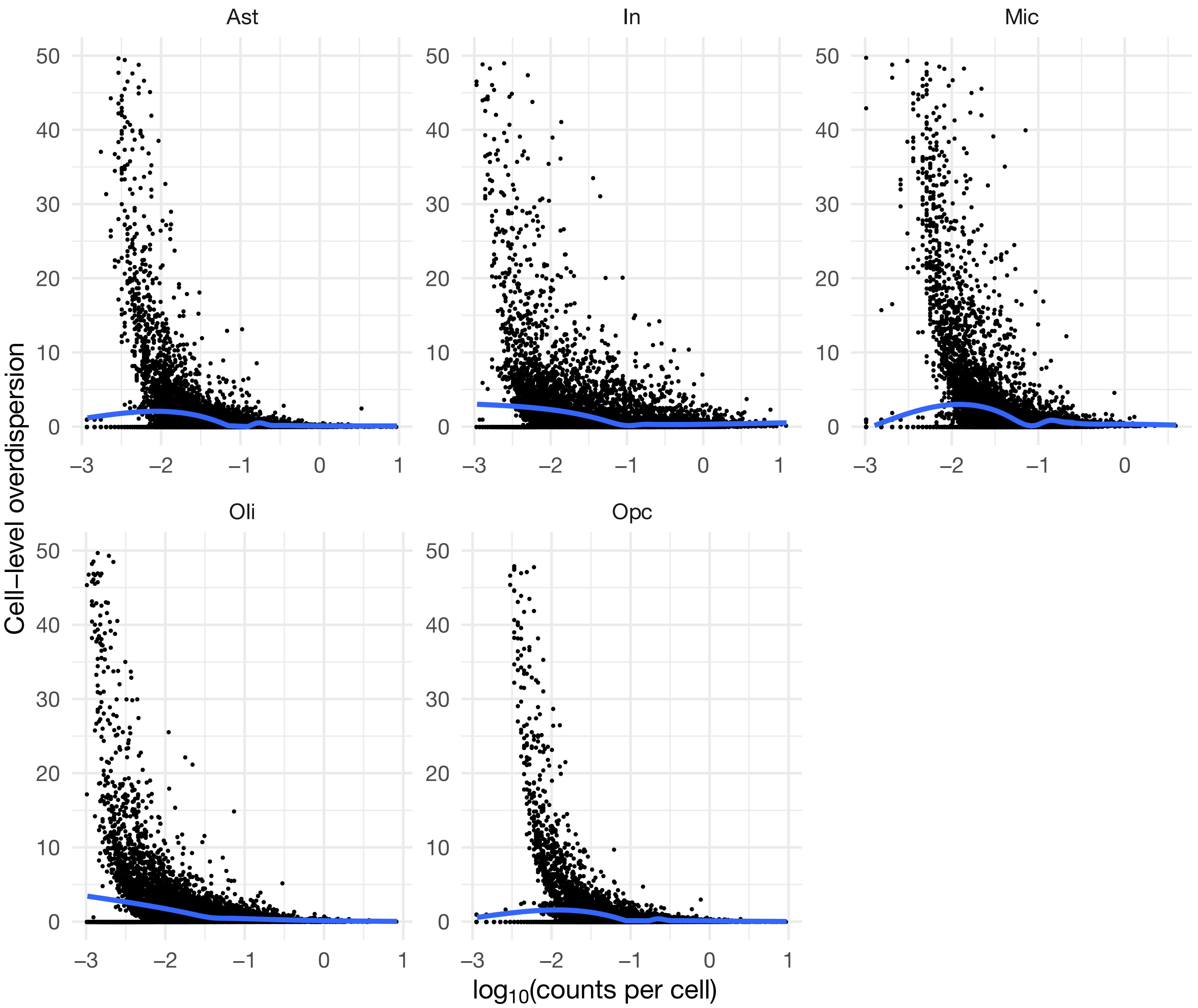
Ast
In
Mic
Oli
Opc
Cell−level overdispersion
log10(counts per cell)
50
40
30
20
10
0
−3
−2
−1
0
1

Supplementary Figure S10. The subject-level overdispersion of genes with CPC>0.1% in five major cell types in the frontal cortex estimated by NEBULA with respect to the CPC of the gene. No covariates other than the intercept were included in the model.

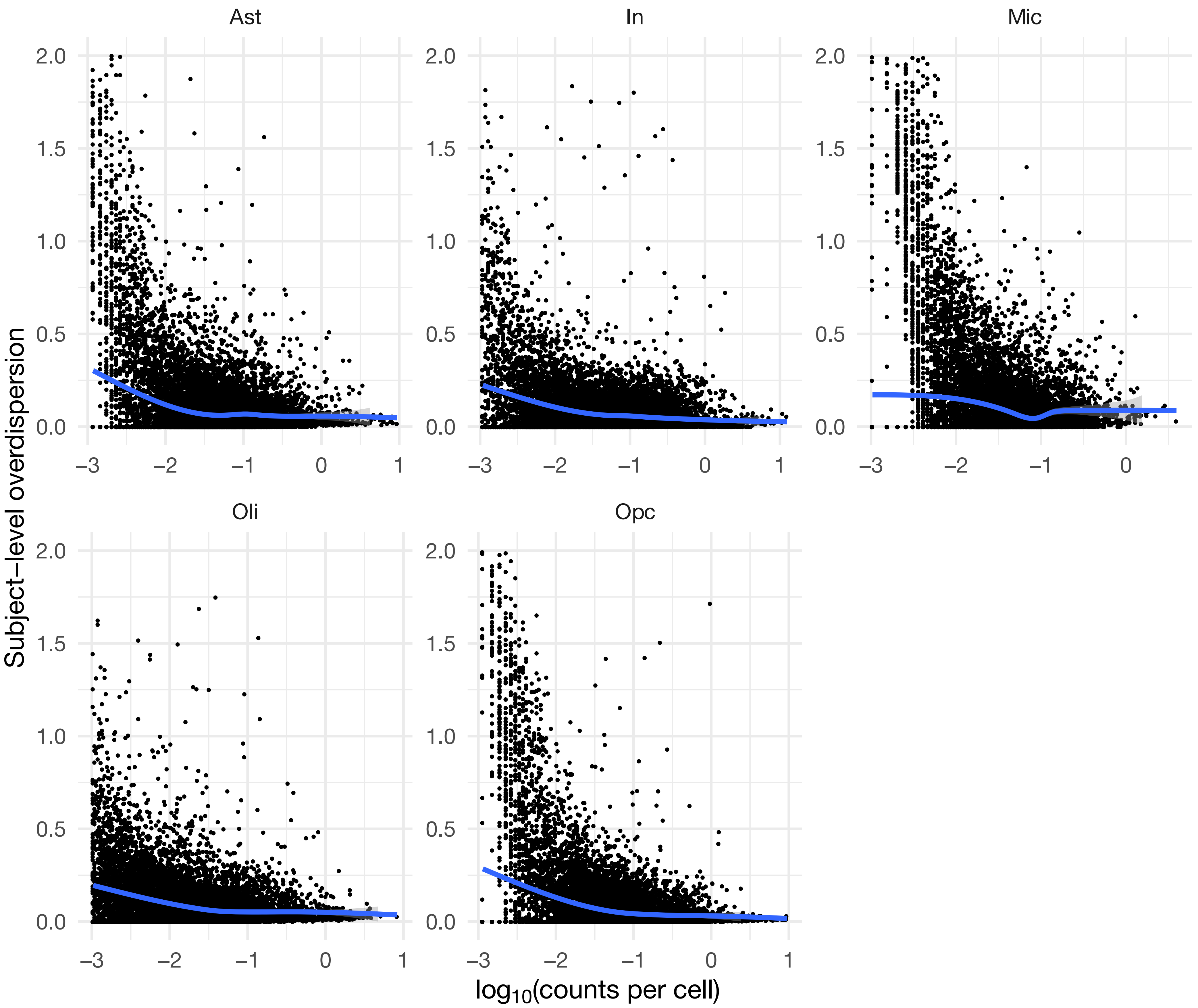
Ast
In
Mic
Oli
Opc
Subject−level overdispersion
log10(counts per cell)
2.0
1.5
1.0
0.5
0.0
−3
−2
−1
0
1

Supplementary Figure S11. The distribution of (A) the estimated cell-level overdispersion and (B) subject-level overdispersion in the excitatory neurons (Ex) and the 11 subpopulations in the excitatory neurons (Ex0-Ex12) annotated in the ROSMAP 48-subject snRNA-seq data set. All genes with CPC>0.1% in each of the cell populations were included.

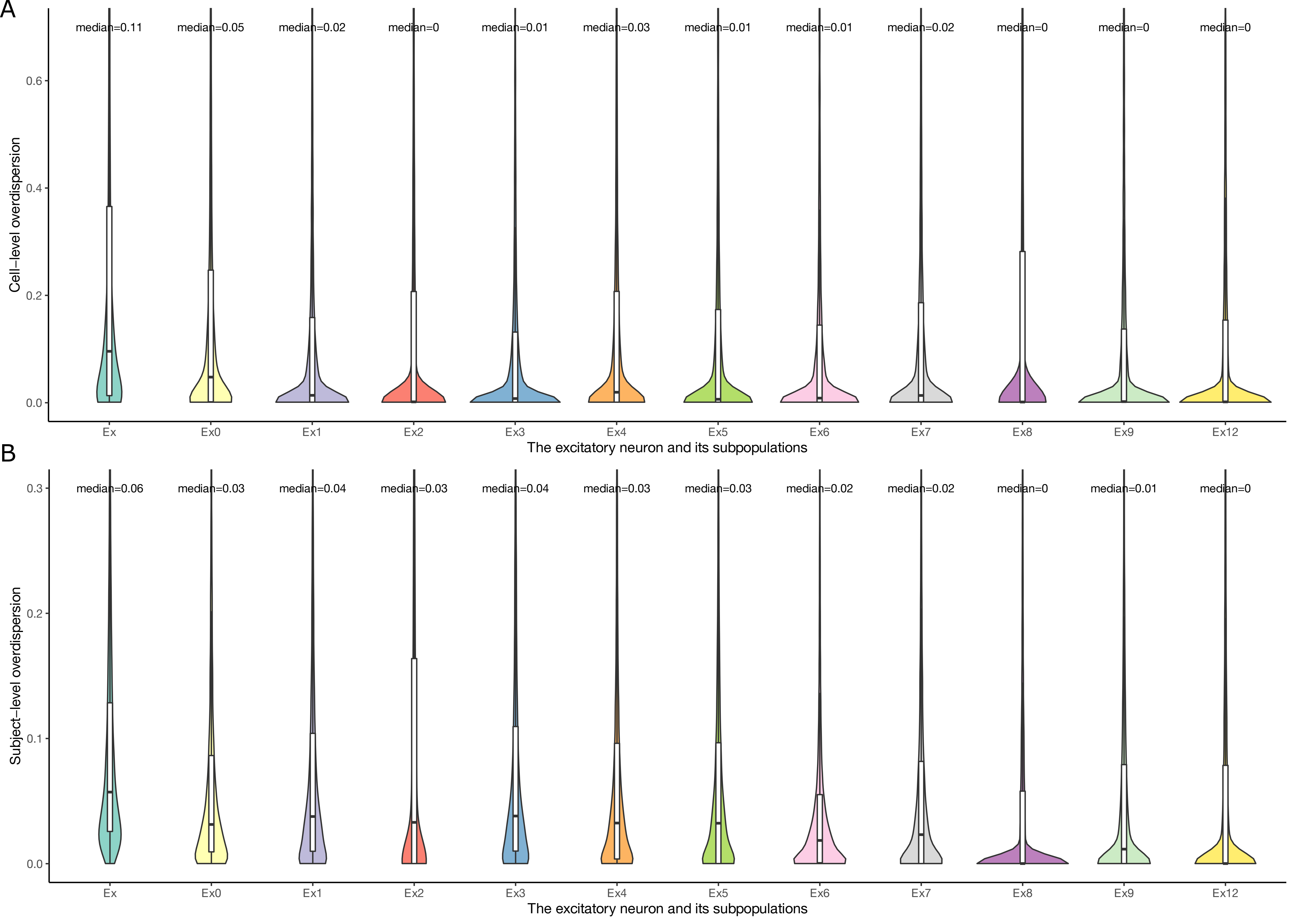

A
median=0.11
median=0.05
median=0.02
median=0
median=0.01
median=0.03
median=0.01
median=0.01
median=0.02
median=0
median=0
median=0
Cell−level overdispersion
0.0
0.2
0.4
0.6
Ex
Ex0
Ex1
Ex2
Ex3
Ex4
Ex5
Ex6
Ex7
Ex8
Ex9
Ex12
The excitatory neuron and its subpopulations
B
median=0.06
median=0.03
median=0.04
median=0.03
median=0.04
median=0.03
median=0.03
median=0.02
median=0.02
median=0
median=0.01
median=0
Subject−level overdispersion
0.0
0.1
0.2
0.3
Ex
Ex0
Ex1
Ex2
Ex3
Ex4
Ex5
Ex6
Ex7
Ex8
Ex9
Ex12
The excitatory neuron and its subpopulations

Supplementary Figure S12. The cell-level overdispersion estimated by NEBULA versus those estimated by *glmer.nb* with nAGQ=0 for 16,207 genes with CPC>0.1% in the excitatory neurons in the ROSMAP 48-subject snRNA-seq data set.

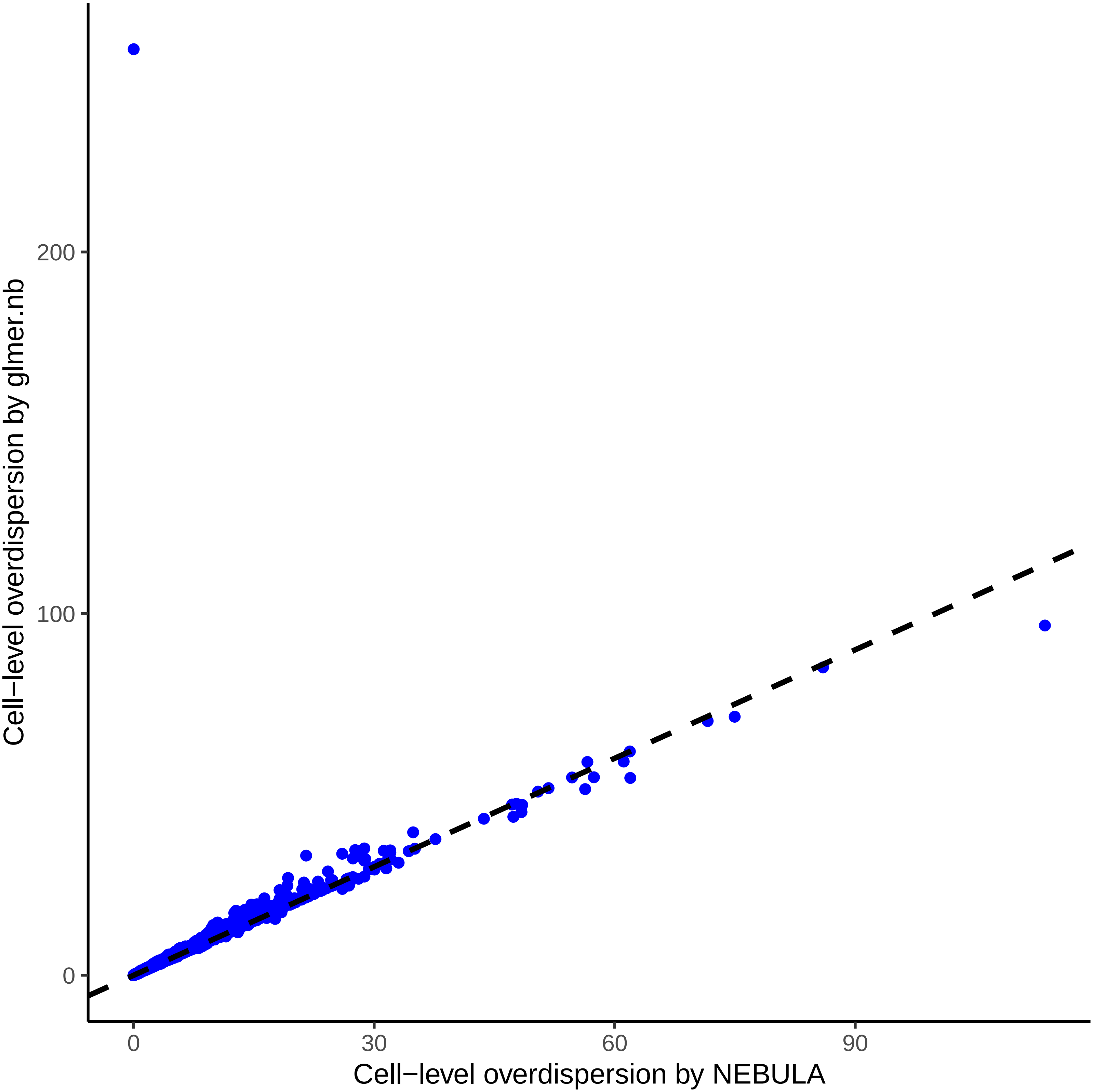
Cell−level overdispersion by glmer.nb
200
100
0
0
30
60
90
Cell−level overdispersion by NEBULA

Supplementary Figure S13. The subject-level overdispersion estimated by NEBULA versus those estimated by *glmer.nb* with nAGQ=0 for 16,207 genes with CPC>0.1% in the excitatory neurons in the ROSMAP 48-subject snRNA-seq data set.

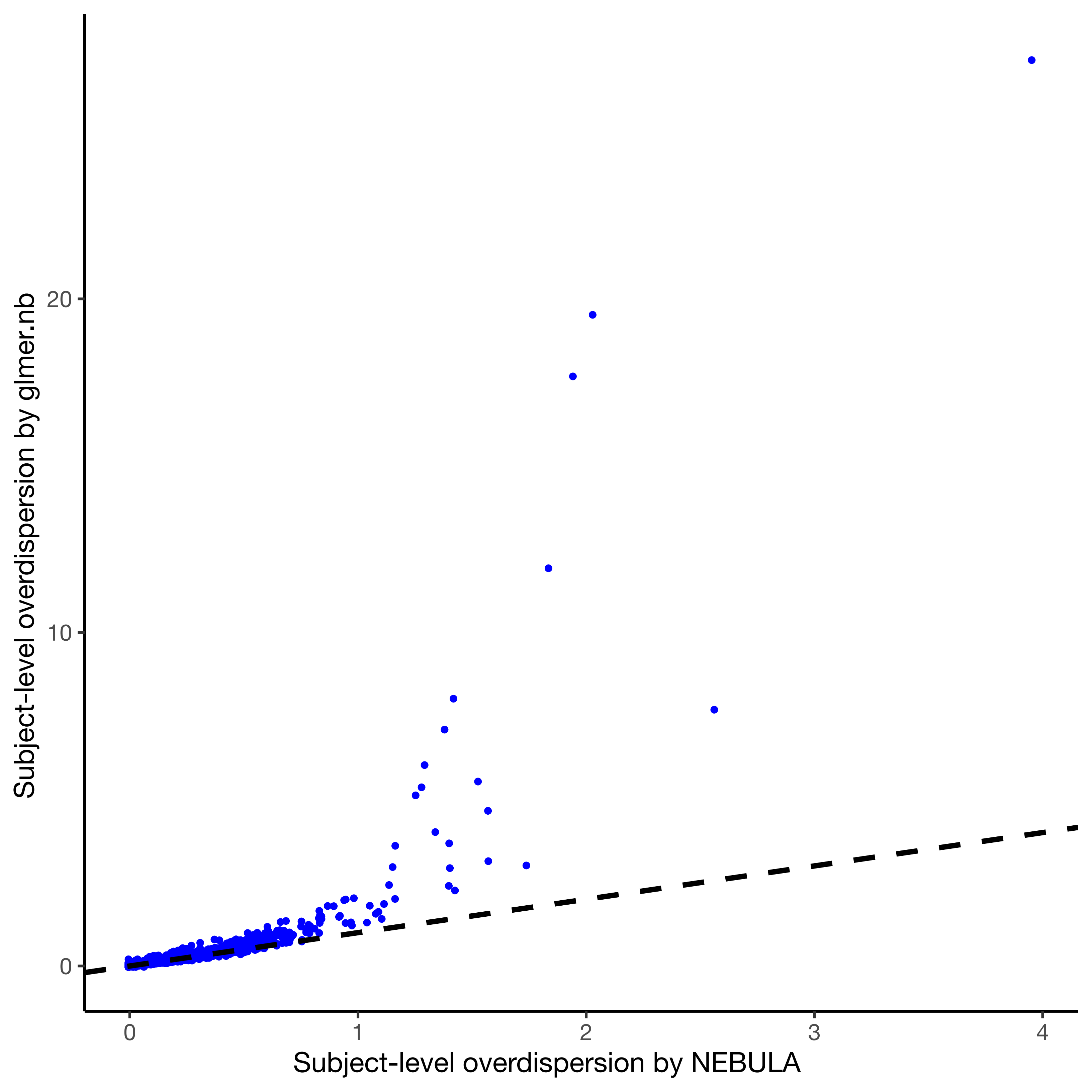

Subject-level overdispersion by glmer.nb
20
10
0
0
1
2
3
4
Subject-level overdispersion by NEBULA

Supplementary Figure S14. The log(FC) of a subject-level variable estimated by NEBULA versus those estimated by *glmer.nb* with nAGQ=0 for 16,207 genes with CPC>0.1% in the excitatory neurons in the ROSMAP 48-subject snRNA-seq data set.

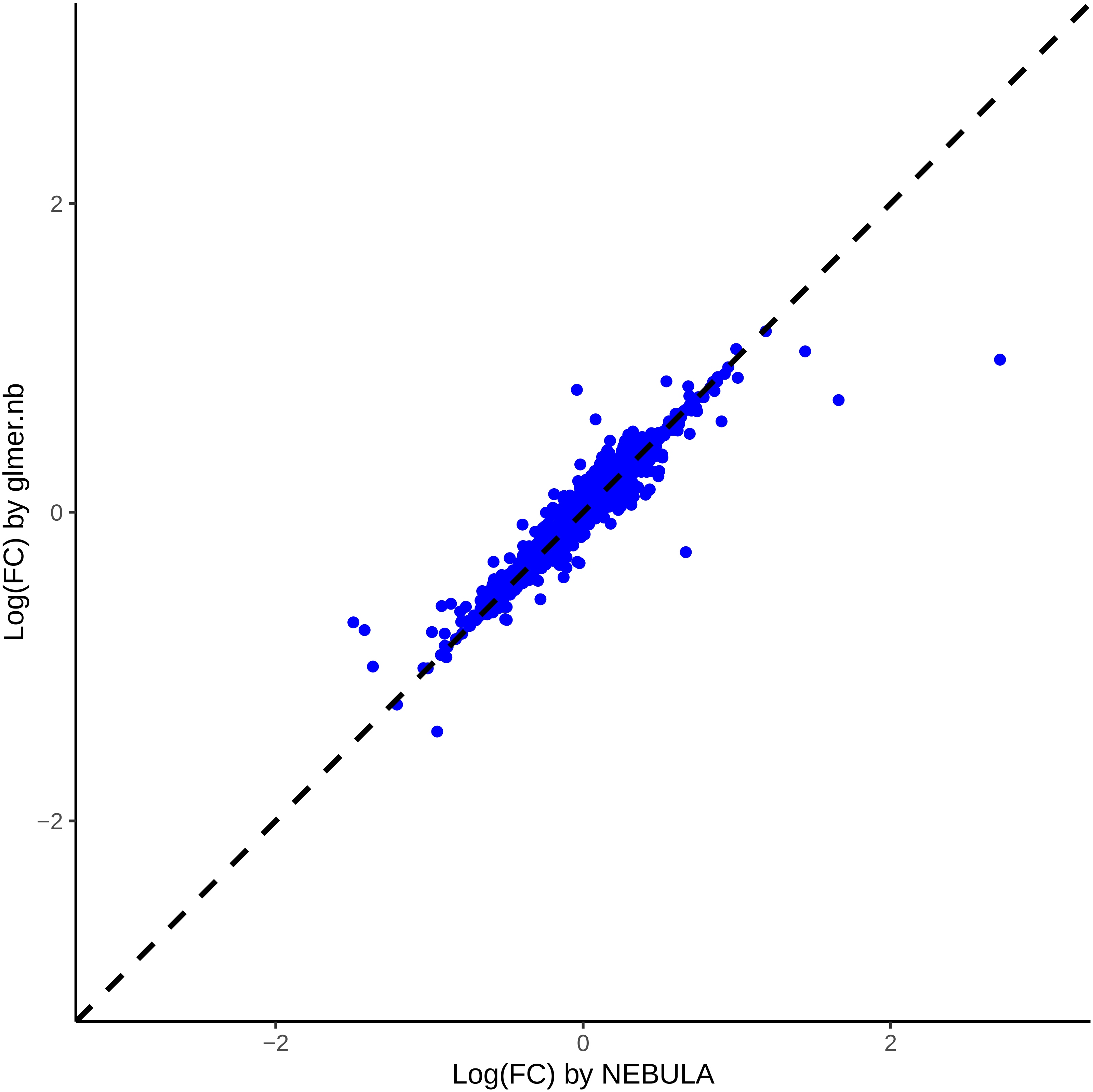

Log(FC) by glmer.nb
Log(FC) by NEBULA
2
0
−2
−2
0
2

Supplementary Figure S15. The cell frequency of the four subclusters in astrocytes across the 48 individuals. The 3386 astrocytes in the human frontal cortex are from the ROSMAP 48-subject snRNA-seq data.

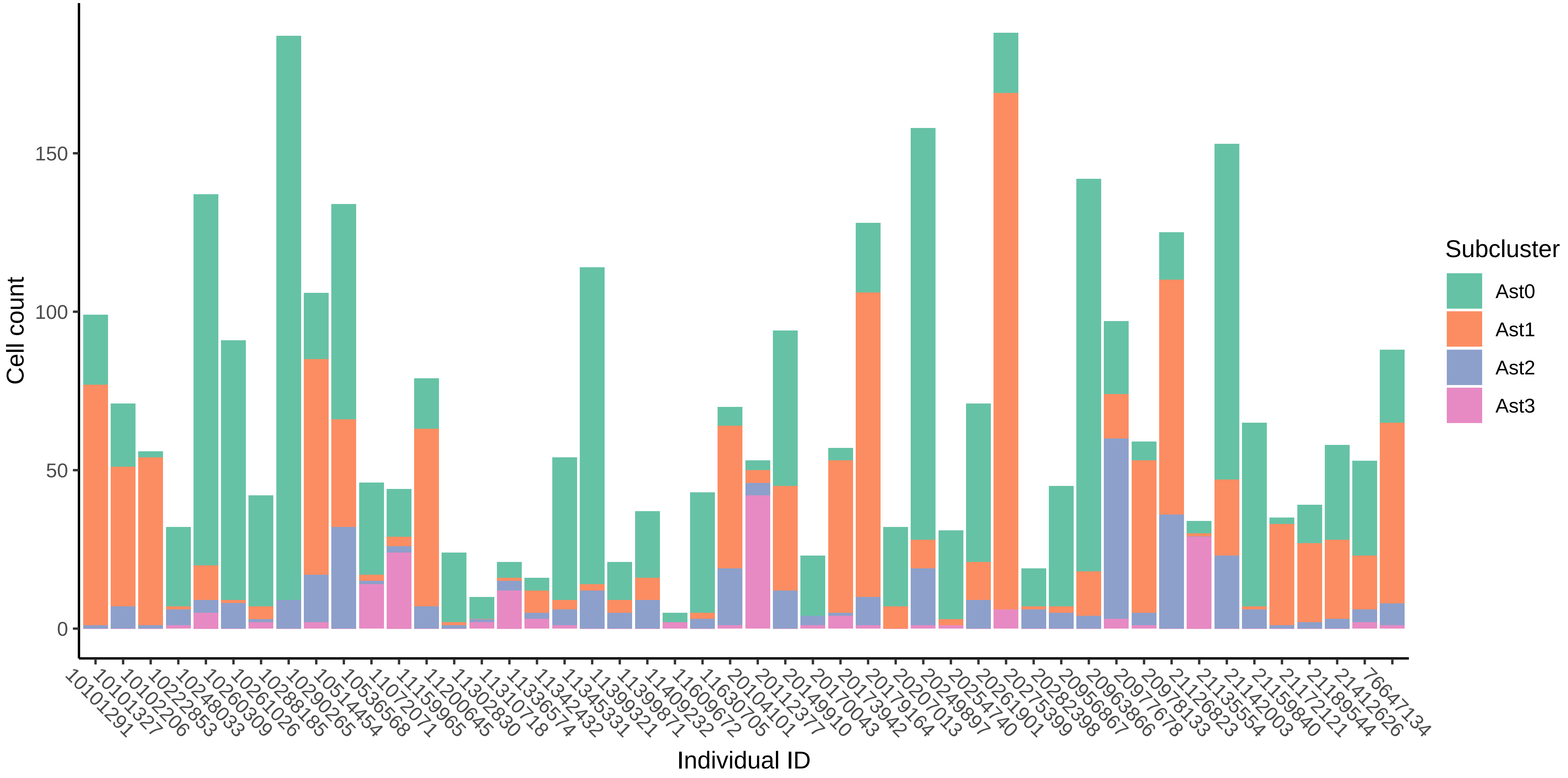

Cell count
150
100
50
0
10101291
10101327
10102206
10222853
10248033
10260309
10261026
10288185
10290265
10514454
10536568
11072071
11159965
11200645
11302830
11310718
11336574
11342432
11345331
11399321
11399871
11409232
11609672
11630705
20104101
20112377
20149910
20170043
20173942
20179164
20207013
20249897
20254740
20261901
20275399
20282398
20956867
20963866
20977678
20978133
21126823
21135554
21142003
21159840
21172121
21189544
21412626
76647134
Individual ID
Subcluster
Ast0
Ast1
Ast2
Ast3

Supplementary Figure S16. Scatter plots of the normalized expression for the calculation of the Pearson correlation in the co-expression analysis of *BCL3* with two genes (*CX3CR1* and *LINC02446*) in the memory CD4+ T cell population. The outliers are highlighted in red.

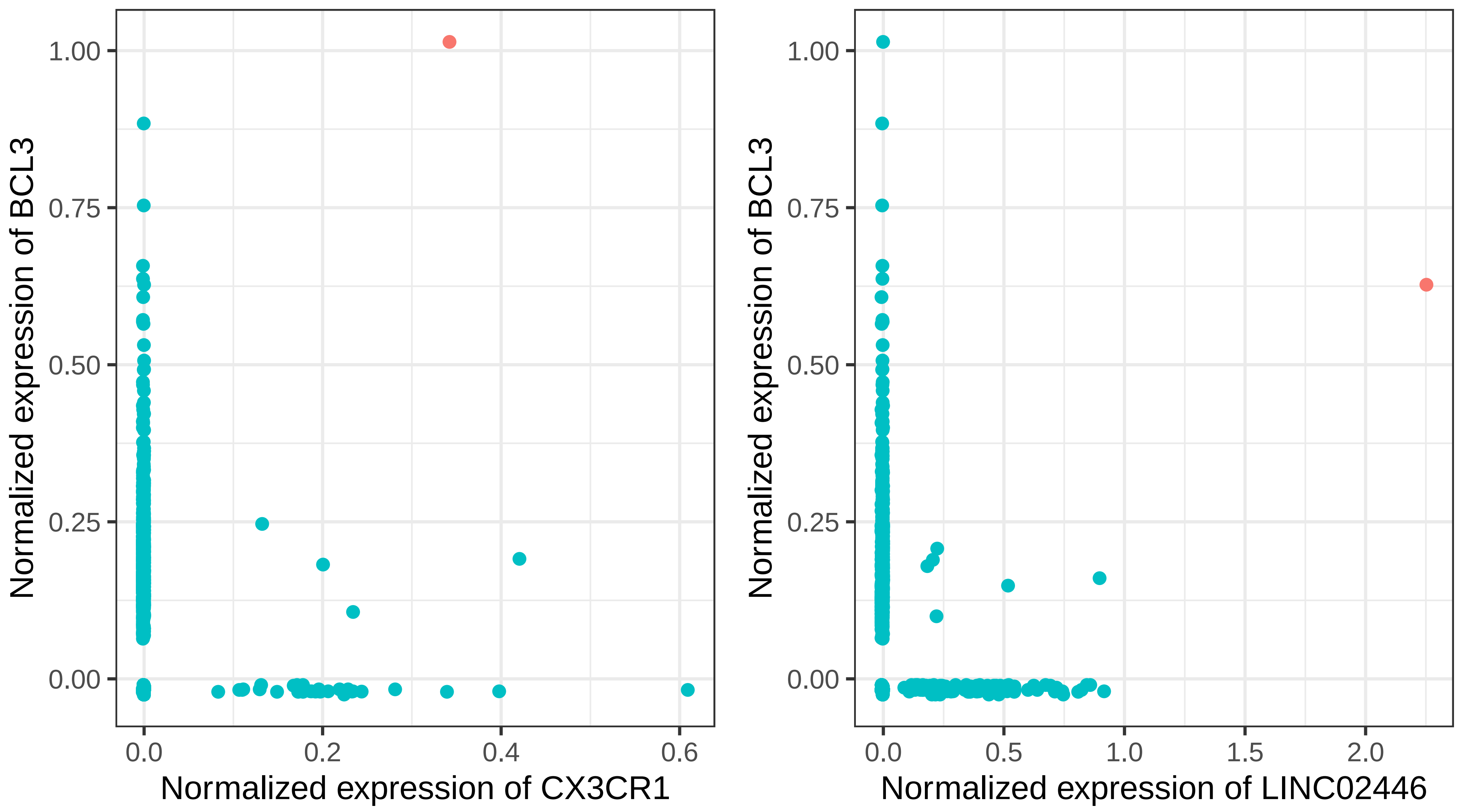

Normalized expression of BCL3
1.00
0.75
0.50
0.25
0.00
0.0
0.2
0.4
0.6
Normalized expression of CX3CR1
Normalized expression of BCL3
1.00
0.75
0.50
0.25
0.00
0.0
0.5
1.0
1.5
2.0
Normalized expression of LINC02446

Supplementary Figure S17. The comparison of the performance of estimating σ^2 between the first-order and the higher-order LA methods. The simulation results are based on the parameter setting with $\phi = 2$, CPS=100, and 30 subjects. The dashed horizontal lines are the true value of σ^2. The summary statistics were calculated from n=500 simulated replicates in each of the scenarios.

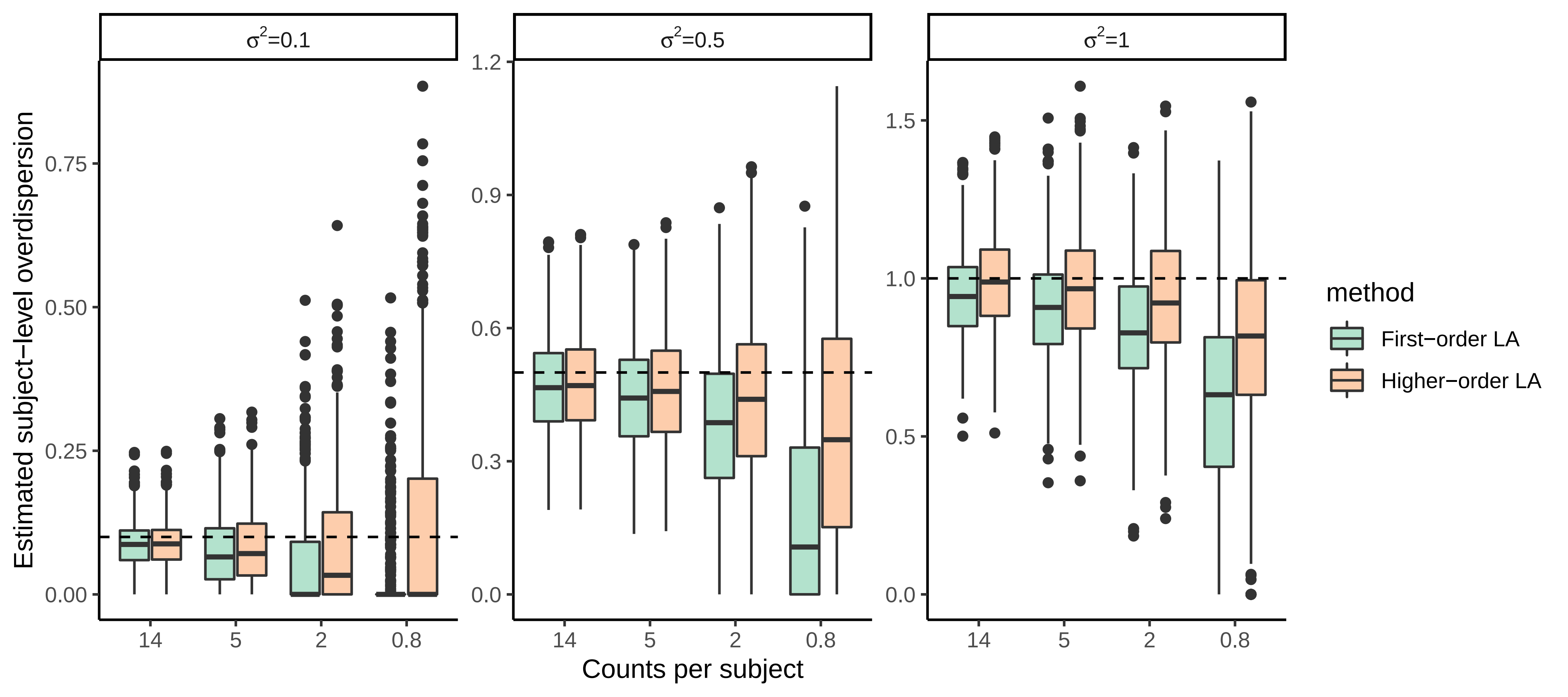

σ²=0.1
σ²=0.5
σ²=1
Estimated subject−level overdispersion
Counts per subject
0.00
0.25
0.50
0.75
0.0
0.3
0.6
0.9
1.2
0.0
0.5
1.0
1.5
14
5
2
0.8
method
First−order LA
Higher−order LA

Supplementary Figure S18. The comparison between the h-likelihoods of the NBGMM and NBLMM. The sample size in all h-likelihoods is 100. The plotted h-likelihood functions with respect to η_i are based on the parameter setting with $\phi = 1$, $\sigma^2 = 0.5$, and $\beta_0 = -5$. (A) An h-likelihood of the NBGMM in which there are no positive counts in the sample. (B) An h-likelihood of the NBGMM in which there are two positive counts in the sample. (C) An h-likelihood of the NBLMM in which there are no positive counts in the sample.

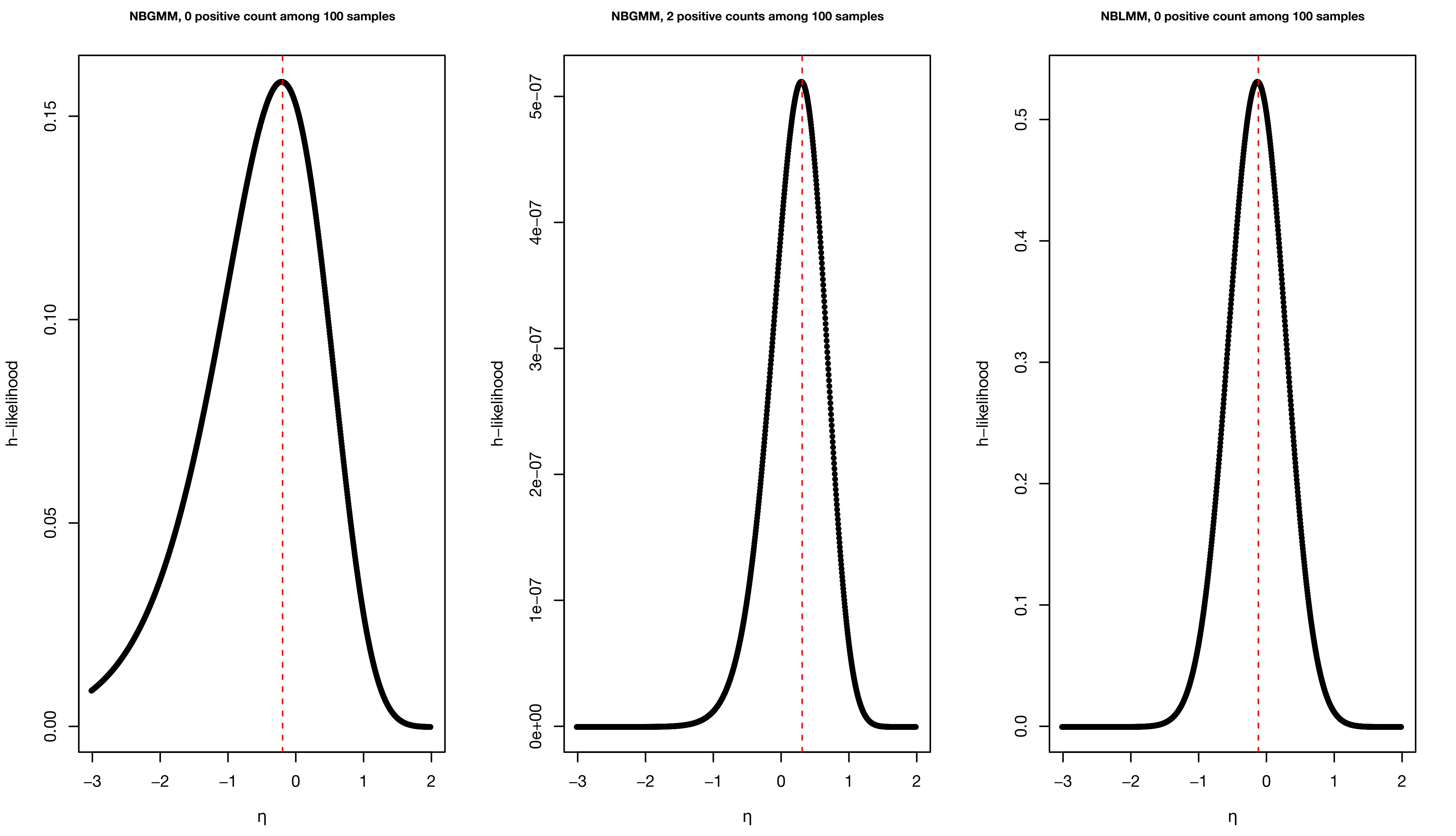

NBGMM, 0 positive count among 100 samples
NBGMM, 2 positive counts among 100 samples
NBLMM, 0 positive count among 100 samples
h−likelihood
η
0.00
0.05
0.10
0.15
0e+00
1e−07
2e−07
3e−07
4e−07
5e−07
0.0
0.1
0.2
0.3
0.4
0.5
−3
−2
−1
0
1
2
