# Supplementary material for: NEBULA is a fast negative binomial mixed model for differential or co-expression analysis of large-scale multi-subject single-cell data
Source: Commun Biol. 2021 May 26;4:629. doi: 10.1038/s42003-021-02146-6 (PMC8155058; doi:10.1038/s42003-021-02146-6)
Supplement: Supplementary file 2 — Description of Additional Supplementary Files [file 42003_2021_2146_MOESM2_ESM.pdf]

Description of Additional Supplementary Files

File name: Supplementary Data 1

Description: The proposed strategy for testing fixed-effects variables used in the analysis of the real data. This table summarizes the conditions under which an estimation algorithm (NEBULA-LN or NEBULA-HL) is used to estimate each of the over-dispersion parameters.

File name: Supplementary Data 2

Description: Results of the cell-level co-expression analysis of BCL3 and TCF7 in memory CD4+ T cell populations in the PBMC scRNA-seq data.

File name: Supplementary Data 3

Description: A list of top 1000 genes whose expression was correlated with the expression of APOE at the single-cell level in astrocytes and microglia in the human frontal cortex based on the p-values of the meta-analysis of the ROSMAP 48-subject and 32-subject snRNA-seq data sets. logFC_48(32): The log(fold change) of the association between the expression of the gene and APOE in the ROSMAP 48(32)-subject snRNA-seq data set. logFC>0 means a positive correlation, and logFC<0 means a negative correlation with the expression of APOE.

File name: Supplementary Data 4

Description: A list of top 200 genes whose expression was correlated with the expression of APOE stratified by the APOE variants at the single-cell level in astrocytes in the human frontal cortex from the ROSMAP 48-subject snRNA-seq data set. logFC: The log(fold change) of the association between the expression of the gene and APOE. logFC>0 means a positive correlation, and logFC<0 means a negative correlation with the expression of APOE. APOE2: APOE e2e3. APOE3: APOE e3e3. APOE4: APOE e3e4 and APOE e4e4.

File name: Supplementary Data 5

Description: A list of top 200 genes whose expression was correlated with the expression of APOE stratified by the APOE variants at the single-cell level in microglia in the human frontal cortex from the ROSMAP 48-subject snRNA-seq data set. logFC: The log(fold change) of the association between the expression of the gene and APOE. logFC>0 means a positive correlation, and logFC<0 means a negative correlation with the expression of APOE. APOE2: APOE e2e3. APOE3: APOE e3e3. APOE4: APOE e3e4 and APOE e4e4.

File name: Supplementary Data 6

Description: A list of the marker genes identified by NEBULA to annotate the cell clusters in the PBMC scRNA-seq data set. Proportion of cells with presence: the percentage of cells having a positive count of the gene in that cluster. log(FC): the estimated log(FC) of this gene by NEBULA.
