# Supplementary material for: NEBULA is a fast negative binomial mixed model for differential or co-expression analysis of large-scale multi-subject single-cell data
Source: Commun Biol. 2021 May 26;4:629. doi: 10.1038/s42003-021-02146-6 (PMC8155058; doi:10.1038/s42003-021-02146-6)
Supplement: Supplementary file 9 — Reporting Summary [file 42003_2021_2146_MOESM9_ESM.pdf]

nature research

Corresponding author(s): Liang He, Manolis Kellis, Alexander Kulminski

Last updated by author(s): 2021/04/13

Reporting Summary

Nature Research wishes to improve the reproducibility of the work that we publish. This form provides structure for consistency and transparency in reporting. For further information on Nature Research policies, see our Editorial Policies and the Editorial Policy Checklist.

Statistics

For all statistical analyses, confirm that the following items are present in the figure legend, table legend, main text, or Methods section.

n/a	Confirmed	
[ ]	[x]	The exact sample size (*n*) for each experimental group/condition, given as a discrete number and unit of measurement
[ ]	[x]	A statement on whether measurements were taken from distinct samples or whether the same sample was measured repeatedly
[ ]	[x]	The statistical test(s) used AND whether they are one- or two-sided<br>*Only common tests should be described solely by name; describe more complex techniques in the Methods section.*
[ ]	[x]	A description of all covariates tested
[ ]	[x]	A description of any assumptions or corrections, such as tests of normality and adjustment for multiple comparisons
[ ]	[x]	A full description of the statistical parameters including central tendency (e.g. means) or other basic estimates (e.g. regression coefficient) AND variation (e.g. standard deviation) or associated estimates of uncertainty (e.g. confidence intervals)
[ ]	[x]	For null hypothesis testing, the test statistic (e.g. *F*, *t*, *r*) with confidence intervals, effect sizes, degrees of freedom and *P* value noted<br>*Give P values as exact values whenever suitable.*
[ ]	[x]	For Bayesian analysis, information on the choice of priors and Markov chain Monte Carlo settings
[ ]	[x]	For hierarchical and complex designs, identification of the appropriate level for tests and full reporting of outcomes
[ ]	[x]	Estimates of effect sizes (e.g. Cohen's *d*, Pearson's *r*), indicating how they were calculated

Our web collection on statistics for biologists contains articles on many of the points above.

Software and code

Policy information about availability of computer code

Data collection	synapseclient v2.3.0 (https://pypi.org/project/synapseclient/)
Data analysis	NEBULA v1.1.7 (https://github.com/lhe17/nebula), Seurat v4.0.0 (https://cran.r-project.org/web/packages/Seurat/index.html), scCATCH v2.1 (https://github.com/ZJUFanLab/scCATCH), lme4 v1.1-26 (https://cran.r-project.org/web/packages/lme4/index.html), glmmTMB v1.0.2.1 (https://cran.r-project.org/web/packages/glmmTMB/index.html), MASS 7.3-53.1 (https://cran.r-project.org/web/packages/MASS/index.html), INLA v20.04.18 (https://www.r-inla.org/download-install)

For manuscripts utilizing custom algorithms or software that are central to the research but not yet described in published literature, software must be made available to editors and reviewers. We strongly encourage code deposition in a community repository (e.g. GitHub). See the Nature Research guidelines for submitting code & software for further information.

Data

Policy information about availability of data

All manuscripts must include a data availability statement. This statement should provide the following information, where applicable:

- Accession codes, unique identifiers, or web links for publicly available datasets
- A list of figures that have associated raw data
- A description of any restrictions on data availability

This manuscript was prepared using limited access datasets obtained through Synapse (https://www.synapse.org/#!Synapse:syn3219045, https://www.synapse.org/#!Synapse:syn21670836, https://www.synapse.org/#!Synapse:syn18485175) and dbGaP (accession numbers: phs002222.v1.p1 (MS PBMC), phs001703.v3.p1 (DICE)).

Field-specific reporting

Please select the one below that is the best fit for your research. If you are not sure, read the appropriate sections before making your selection.

☒ Life sciences ☐ Behavioural & social sciences ☐ Ecological, evolutionary & environmental sciences

For a reference copy of the document with all sections, see nature.com/documents/nr-reporting-summary-flat.pdf

Life sciences study design

All studies must disclose on these points even when the disclosure is negative.

Sample size	We selected by far the two of the largest snRNA-seq data sets in the frontal cortex for Alzheimer's disease and one of the largest scRNA-seq data sets in PBMC from a cohort of multiple sclerosis in terms of the number of cells.
Data exclusions	We use three single-cell data sets in our real data analysis including the ~70k PBMC scRNA-seq data set, the ~80k 48-subject snRNA-seq data in the frontal cortex, and the ~110k 32-subject snRNA-seq data in the frontal cortex. The original data sets are provided by different research groups using different preprocessing steps. Briefly, in the two snRNA-seq data sets, we used the data after QC and the cell-type annotation provided by the data generators, while for the scRNA-seq data set we performed QC, clustering, and cell-type annotation for the scRNA-seq data by ourselves as this information is not available from the original publication. As a part of the analysis of the two snRNA-seq data sets, we performed a further QC step to remove potential outlier cells in order to be more stringent.
Replication	We used multiple methods and data sets to demonstrate that our results are reproducible.
Randomization	Not available to this study.
Blinding	Not available to this study.

Reporting for specific materials, systems and methods

We require information from authors about some types of materials, experimental systems and methods used in many studies. Here, indicate whether each material, system or method listed is relevant to your study. If you are not sure if a list item applies to your research, read the appropriate section before selecting a response.

Materials & experimental systems

n/a	Involved in the study	
☒	☐	Antibodies
☒	☐	Eukaryotic cell lines
☒	☐	Palaeontology and archaeology
☒	☐	Animals and other organisms
☒	☐	Human research participants
☒	☐	Clinical data
☒	☐	Dual use research of concern

Methods

n/a	Involved in the study	
☒	☐	ChIP-seq
☒	☐	Flow cytometry
☒	☐	MRI-based neuroimaging
